# Supplementary material for: Mouse-Derived Isograft (MDI) In Vivo Tumor Models I. Spontaneous sMDI Models: Characterization and Cancer Therapeutic Approaches
Source: Cancers (Basel). 2019 Feb 19;11(2):244. doi: 10.3390/cancers11020244 (PMC6406567; doi:10.3390/cancers11020244)
Supplement: Supplementary file 1 [file cancers-11-00244-s001.pdf]

cancers

MDPI

Type of the Paper (Article- Supplementary Data)

Mouse-Derived Isograft (MDI) *in vivo* Tumor Models

I. Spontaneous sMDI Models: Characterization and Cancer Therapeutic Approaches

- Supplementary Data -

Peter Jantscheff [1a]*, Janette Beshay [1a], Thomas Lemarchand [2], Cynthia Obodozie [1], Christoph Schächtele [1], Holger Weber [1,*]

[1]*In Vivo* Pharmacology, Proqinase GmbH, Breisacher Str. 117, 79106 Freiburg, Germany, peter.jantscheff@t-online.de; Janette_Beshay@web.de; c.obodozie@proqinase.com; c.schaechtele@proqinase.com; h.weber@proqinase.com
[2]TPL Pathology Labs, Sasbacher Str. 10, 79111 Freiburg, Germany, lemarchand@tpl-path-labs.com
*Correspondence: peter.jantscheff@t-online.de; Tel: +49-7666-913-0396; h.weber@proqinase.com ; Tel.: +49-761-769996-17331733
[a]Peter Jantscheff and Janette Beshay contributed equally to this work.
Received: date; Accepted: date; Published: date

1. Supplementary Data

Suppl_data_sMDI_cMDI_models – Cancers.docx – containing:

1.1 Tab. S1 – sMDI_RNA-Seq *p 02*

Tab. S1a - sMDI_RNA-Seq: Expression of tyrosine kinase receptor (TKR) genes in selected sMDI JA-0009, and cMDI JA-2011 and JA-2042

Tab. S1b - sMDI_RNA-Seq: Expression of immune population marker genes (T-, B-, NK-cells, Mφ, myeloid cells) in selected sMDI JA-0009, and cMDI JA-2011 and JA-2042

Tab. S1c - sMDI_RNA-Seq: Expression of IFN-y signature genes in selected sMDI JA-0009, and cMDI JA-2011 and JA-2042

1.2 Fig. S1 – sMDI_RNA-Seq *p 07*

Fig. S1a i – iii – **sMDI_RNA-Seq:** FPKM-Values

Fig. S1b i – iii – **sMDI_RNA-Seq:** Internal Relative Expression

1.3 Doc. S1-sMDI: sMDI - Histological and Pathological Analysis *p 11*

The document summarizes detailed histopathological analysis of primary and derived tumors of various sMDI models inclusive respective large microphotographs

1.4 Doc. S2-sMDI *p 58*

Material, Methods & Results: Efficacy studies with anti-mPD-1 and anti-mCTLA-4 in seven syngeneic standard mouse tumor models

1.5 Fig. S2-sMDI *p 59*

Efficacy studies with anti-mPD-1 and anti-mCTLA-4 in seven syngeneic standard mouse tumor models

1.6 Doc. S3-sMDI *p 61*

Methods & Results: Establishment, efficacy study and flow cytometric characterization of mPD-1-resistant MC38-CEA relapsed subline

1.7 Fig. S3-sMDI *p 62*

Establishment, efficacy study and flow cytometric characterization of mPD-1-resistant MC38-CEA relapsed subline

1.1 *Tab. S1 – sMDI_RNA-Seq*

Tab. S1a - sMDI_RNA-Seq: Expression of tyrosine kinase receptor (TKR) genes in selected sMDI JA-0009, and cMDI JA-2011 and JA-2042

Gene	Gene description	Gene expression (FPKM values)					
		JA-0009		JA-2011		JA-2042	
		sample 1	sample 2	sample 1	sample 2	sample 1	sample 2
Kdr	kinase insert domain protein receptor, VEGF-R2	8.579	10.474	7.432	13.084	6.298	8.009
Fgfr1	fibroblast growth factor receptor 1	122.697	138.217	166.383	137.789	112.092	118.213
Fgfr2	fibroblast growth factor receptor 2	1.146	0.031	4.227	2.491	0.937	1.072
Pdgfrb	platelet derived growth factor receptor, beta	40.534	34.803	60.614	51.228	62.505	72.651
Met	met proto-oncogene	120.096	83.597	24.139	30.064	33.087	36.542
Ret	ret proto-oncogene	0.225	0.179	1.058	0.653	0.076	0.084
Flt3	FMS-like tyrosine kinase 3	0.378	0.276	0.240	0.292	1.259	1.437
Axl	AXL receptor tyrosine kinase	172.031	163.433	203.023	210.440	261.356	242.636
Kit	KIT proto-oncogene receptor tyrosine kinase	2.162	1.856	4.323	5.736	5.162	6.012
Egfr	epidermal growth factor receptor	7.592	7.015	9.604	11.132	13.700	15.918
Alk	anaplastic lymphoma kinase	0.087	0.166	0.010	0.021	0.073	0.112
Ephb4	Eph receptor B4	28.023	25.817	13.665	10.399	22.000	24.307
Erbb2	erb-b2 receptor tyrosine kinase 2	7.034	6.063	9.046	7.539	7.018	6.076
Erbb4	erb-b2 receptor tyrosine kinase 4	0.000	0.000	0.000	0.000	0.000	0.000
Igf1r	insulin-like growth factor I receptor	9.052	9.258	8.474	7.896	8.069	8.482
Mst1r	macrophage stimulating 1 receptor (c-	1.309	0.488	4.416	3.608	1.397	0.656
Tek	TEK receptor tyrosine kinase, TIE2	3.116	4.038	12.923	14.449	1.985	2.598
Flt4	FMS-like tyrosine kinase 4, VEGF-R3	2.852	3.971	3.777	5.235	2.801	4.222

Whole transcriptome shotgun sequencing-based (RNA-Seq) Expression pattern of tyrosine kinase receptor (TKR) gene family

RNA-isolation and RNA-Seq were performed by StarSeq, Mainz, Germany as whole transcriptome shotgun sequencing analysis from samples of sMDI JA-0009 and cMDI JA-2011 and JA-2042. RNA-Seq comparisons were performed based on respective FPKM (fragments per kilobase million) values. Since we could not yet determine the definite tissue of origin of the outgrowing MDI tumors, it was not possible to compare tumor gene expression with its respective -unfortunately unknown- normal tissue equivalent. Thus, we performed these experiments as a means of proof of principle, using the example of three gene families which are related to tumor malignancy or anti-tumoral immune response in three different MDIs.

Table S1a summarizes duplicate FPKM values of eighteen genes of tyrosine kinase receptor (TKR) gene family separately determined from single RNA-Seq experiment raw data from two distinct, frozen stored tumor samples of one single passage of JA-0009, JA-2011 or JA-2042 MDI each.

Duplicate FPKM values do reflect an objective gene expression pattern within the individual MDI models. Up to 1,000-fold different expression patterns comparing FPKM values of individual TKR genes were observed. For example, anaplastic lymphoma kinase, Alk (mean FPKM: 0.126), showed very weak (less than 1,000-fold lower) expression, compared with AXL receptor tyrosine kinase, Axl (mean FPKM: 167.732), whereas erb-b2 receptor tyrosine kinase 4, Erbb4, was not expressed at all in sMDI JA-0009 model. However, since normal tissue equivalents of MDIs are unknown, one only could speculate about putative malignancy-dependent variances.

Tab. S1b– sMDI_RNA-Seq - Expression of immune population marker genes (T-, B-, NK-cells, MΦ, myeloid cells) in selected sMDI JA-0009, and cMDI JA-2011 and JA-2042

Gene	Gene description	Gene expression (FPKM values)					
		JA-0009		JA-2011		JA-2042	
		sample 1	sample 2	sample 1	sample 2	sample 1	sample 2
Ptprc	protein tyrosine phosphatase, receptor type, C, CD45	37.323	30.111	16.357	15.704	30.151	28.046
Cd19	CD19 antigen	0.017	0.086	0.025	0.000	0.245	0.111
Cd3d	CD3 antigen, delta polypeptide	0.793	0.873	0.447	0.267	3.658	3.015
Cd4	CD4 antigen	1.952	0.759	1.971	0.965	33.392	30.082
Cd8b1	CD8 antigen, beta chain 1	0.608	0.404	0.875	0.346	4.468	4.068
Foxp3	forkhead box P3	0.124	0.117	0.309	0.123	0.685	0.587
Cd44	CD44 antigen	217.964*	154.191*	80.282	88.770	113.863	115.516
Il2ra	interleukin 2 receptor, alpha chain, CD25	0.508	0.449	0.248	0.112	0.734	0.689

CD11b	integrin alpha M	59.742	47.742	33.733	44.577	22.264	21.607
Adgre1	adhesion G protein-coupled receptor E1, F4/80	35.325	30.841	25.396	23.390	75.824	67.878
Mrc1	mannose receptor, C type 1, CD206	40.398	32.636	70.140	91.794	95.140	82.647
Itgax	integrin alpha X, CD11c	7.283	3.457	3.435	3.349	7.650	7.708
Ncr1	natural cytotoxicity triggering receptor 1, NKp46	4.617	3.767	0.354	0.286	2.626	1.900
Itga2	integrin alpha 2, CD49b	5.220	3.428	1.137	1.731	1.189	1.887
Ly6C	lymphocyte antigen 6 complex, locus C1	303.057	337.000	41.151	51.858	41.983	36.347

Whole transcriptome shotgun sequencing-based (RNA-Seq) expression pattern of Immune Population Marker (IPM) gene family

Table S1b summarizes duplicate FPKM (fragments per kilobase million) values of fifteen genes of immune population marker (IPM) gene family separately determined from single RNA-Seq experiment raw data from two distinct, frozen stored tumor samples of a single passage of JA-0009, JA-2011 or JA-2042 MDI each.

Duplicate FPKM values do reflect an objective gene expression pattern within the individual MDI models (JA-0009, JA-2011, or JA-2042). Up to 1,000-fold different expression pattern comparing FPKM values of individual IPM genes were observed. For example, CD19 antigen, Cd19 (mean FPKM: 0.052), showed very weak (less than 1,000-fold lower) expression, compared with CD44 antigen, Cd44 (mean FPKM: 186.078), or lymphocyte antigen 6 complex, locus C1, Ly6C (mean FPKM: 320.029) in sMDI JA-0009 model. However, since normal tissue equivalents of MDIs are unknown, one only could speculate about putative malignancy-dependent variances.

Tab. S1c – sMDI_RNA-Seq - Expression of IFN-γ Signature genes in selected sMDI JA-0009, and cMDI JA-2011 and JA-2042

Gene	Gene description	Gene expression (FPKM values)					
		JA-0009		JA-2011		JA-2042	
		sample 1	sample 2	sample 1	sample 2	sample 1	sample 2
Il2rg	interleukin 2 receptor subunit gamma	42.246	39.482	19.460	20.503	62.764	49.006
Cxcr6	chemokine (C-X-C motif) receptor 6	1.257	0.773	1.433	0.318	9.097	6.613
Cd3d	CD3 antigen, delta polypeptide	0.793	0.873	0.447	0.267	3.658	3.015
Cd2	CD2 antigen	1.575	0.898	0.597	0.706	4.033	2.505
Itgal	integrin alpha L	6.752	4.789	3.783	3.901	12.127	11.312

Tagap	T cell activation Rho GTPase activating protein	4.636	5.536	4.918	3.987	7.021	7.502
Ciita	class II transactivator	0.161	0.136	0.659	0.513	14.236	12.366
Ptprc	protein tyrosine phosphatase, receptor	37.323	30.111	16.357	15.704	30.151	28.046
Cxcl9	chemokine (C-X-C motif) ligand 9	4.238	2.831	1.393	0.923	45.016	24.324
Ccl5	C-C motif chemokine ligand 5	41.223	36.996	7.127	7.141	79.711	65.278
Nkg7	natural killer cell group 7	7.640	7.409	2.421	1.104	21.513	20.296
Gzma	granzyme A	24.492	15.757	1.510	1.445	2.996	3.072
Prf1	perforin 1 (pore forming protein)	10.770	6.110	0.600	0.709	2.331	2.125
Ccr5	chemokine (C-C motif) receptor 5	63.335	62.056	15.800	17.280	30.596	28.809
Cd3e	CD3 antigen, epsilon polypeptide	0.818	0.675	1.223	0.438	7.494	6.838
Gzmk	granzyme K	0.940	0.437	0.290	0.000	0.587	1.035
Ifng	interferon gamma	0.039	0.099	0.112	0.000	1.273	0.848
Gzmb	granzyme B	26.809	12.568	0.783	0.792	1.399	0.905
Pdcd1	programmed cell death 1	1.520	1.512	0.676	0.380	6.870	5.895
Slamf6	SLAM family member 6	2.433	2.073	1.518	0.929	3.694	3.940
Cxcl13	chemokine (C-X-C motif) ligand 13	26.792	6.986	0.176	0.566	2.667	0.784
Cxcl10	chemokine (C-X-C motif) ligand 10	78.838	79.382	8.332	6.334	19.421	10.466
Ido1	indoleamine 2,3-dioxygenase 1	0.771	1.136	0.128	0.000	0.033	0.000
Lag3	lymphocyte-activation gene 3	3.108	2.039	2.392	1.374	8.097	6.592
Stat1	signal transducer and activator of transcription 1	44.663	38.088	11.567	10.864	31.248	24.513
Cxcl11	chemokine (C-X-C motif) ligand 11	14.251	11.497	0.686	0.563	2.390	0.793
Il12b	interleukin 12b	0.067	0.042	0.072	0.026	1.628	1.269
Il12a	interleukin 12a	0.218	0.092	0.052	0.056	0.119	0.000
Irf1	interferon regulatory factor 1	53.329	49.470	66.608	44.893	108.433	98.369
Tbx21	T-box 21	0.344	0.327	0.118	0.177	1.057	0.906
Ctla4	cytotoxic T-lymphocyte-associated protein 4	0.491	0.209	0.161	0.103	0.879	0.632
Cd274	CD274 antigen, PD-L1	6.746	4.850	3.991	4.090	4.693	2.803

Whole transcriptome shotgun sequencing-based (RNA-Seq) expression pattern of IFN-y signature (IFNGS) gene family

Table S1c summarizes duplicate FPKM (fragments per kilobase million) values of thirty-two genes of IFN-γ signature (IFNGS) gene family separately determined from single RNA-Seq experiment raw data from two distinct, frozen stored tumor samples of a single passage of JA-0009, JA-2011 or JA-2042 MDI each.

Duplicate FPKM values do reflect an objective gene expression pattern within the individual MDI models (JA-0009, JA-2011, or JA-2042). Up to 1,000-fold different expression pattern comparing FPKM values of individual genes could be observed. For example, interleukin 12b, Il12b (mean FPKM: 0.054), showed very weak (less than 1,000-fold lower) expression, compared with chemokine (C-X-C motif) ligand 10, Cxcl10 (mean FPKM: 79.110) in sMDI JA-0009 model. However, since normal tissue equivalents of MDIs are unknown, one only could speculate about putative malignancy-dependent variances.

1.2 *Fig. S1 – sMDI_RNA-Seq*

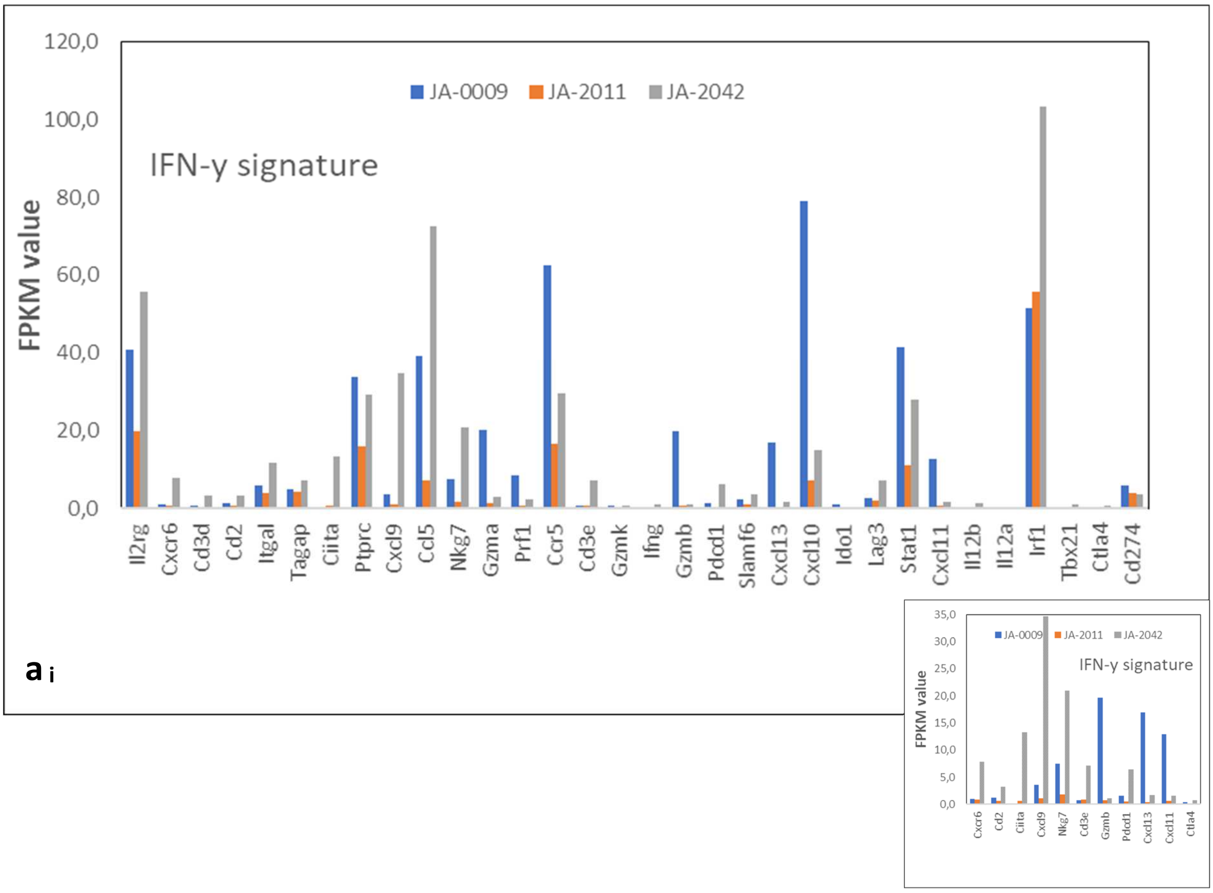

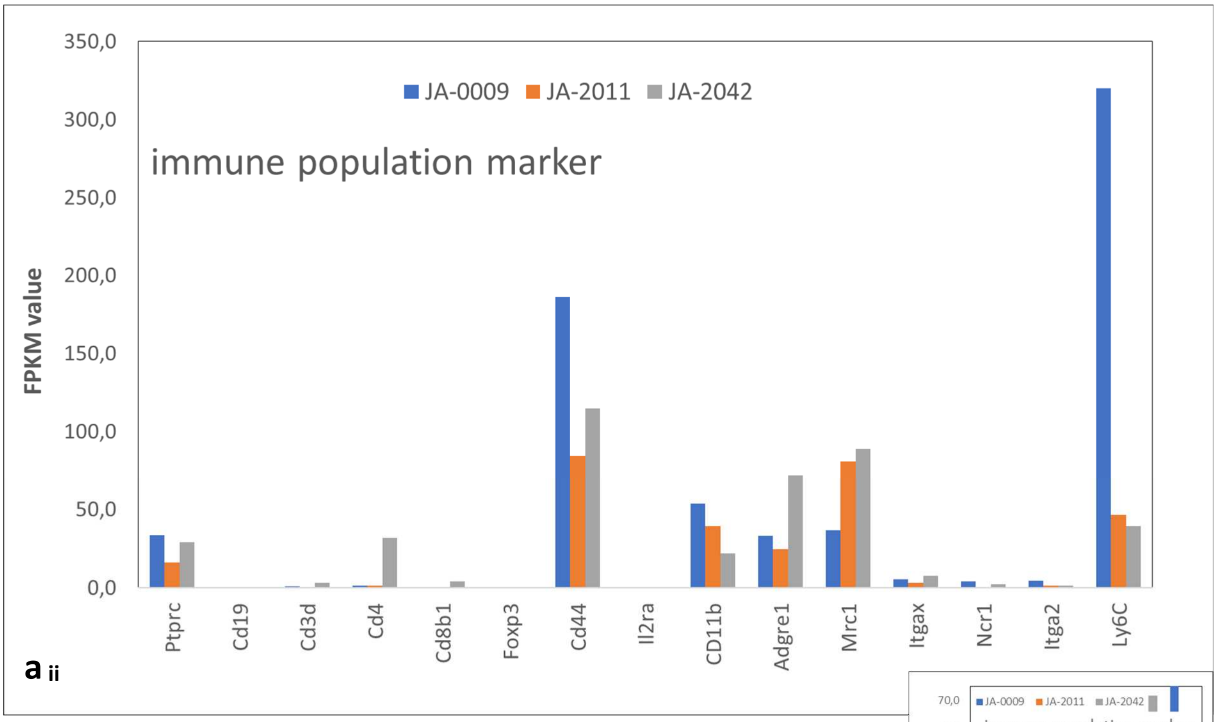

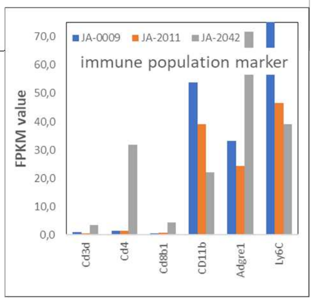

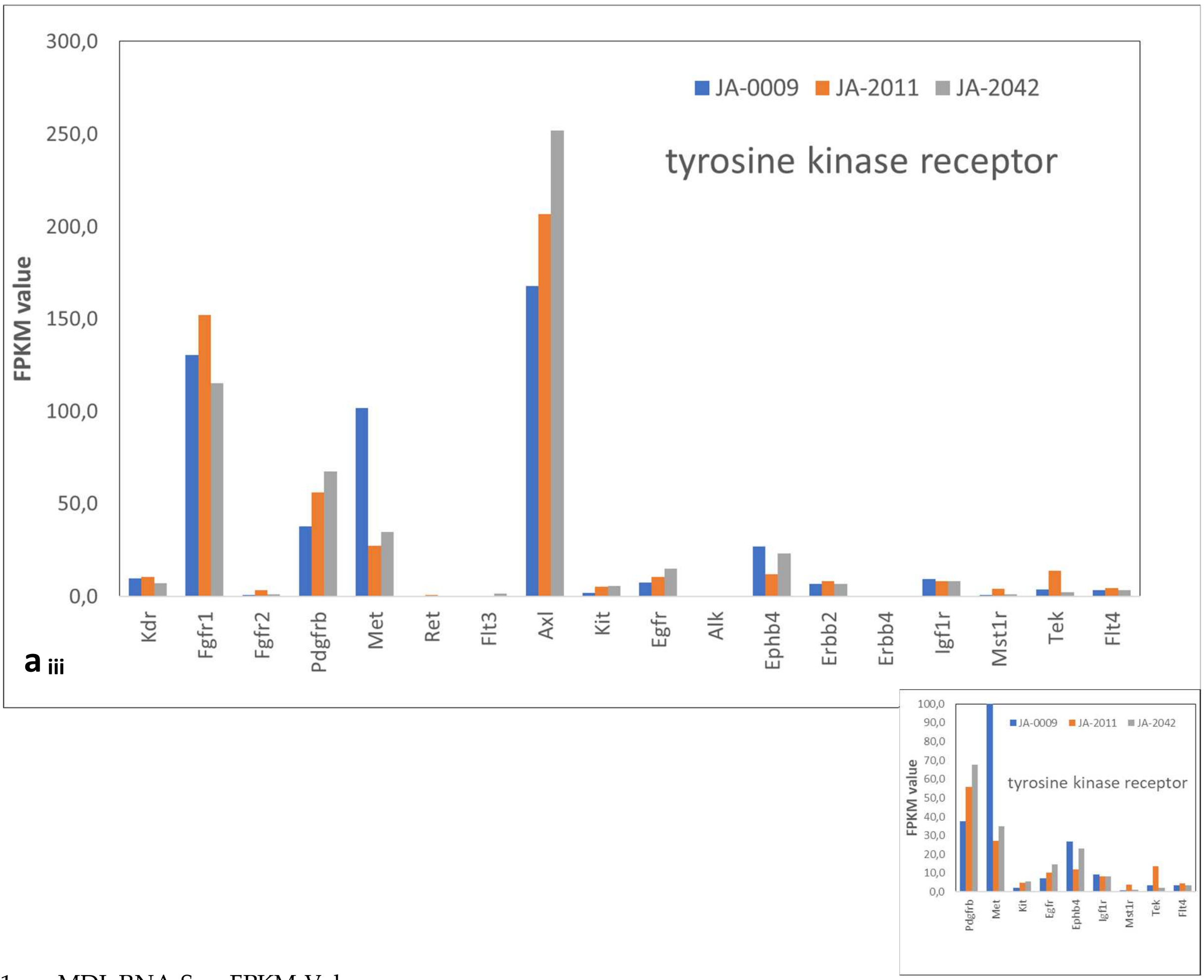

Fig. S1a – sMDI_RNA-Seq: FPKM-Values

Graphical presentation of RNA-seq determined FPKM-Values of various genes of three gene families in three MDI (one sMDI, and two cMDI) models. The observed expression pattern for most tested IFNGS (i), IPM (ii), and TKR (iii) genes was similar in all three MDI models, with high gene expression (FPKM values > 100) of some, low or moderate (FPKM values between 1 – 50) of most other, and not detectable expression (FPKM value = 0) of some further genes. Since the FPKM (fragments per kilobase million) values were not determined in simultaneous experiments a direct comparison of gene expression between the individual MDI models was not possible. Thus, we compared internal relative gene expression (Figs. S1b) in the various models. Small inserts show gene expression of selected gene with other y-axis scaling.

Fig. S1b – sMDI_RNA-Seq: Internal Relative Expression

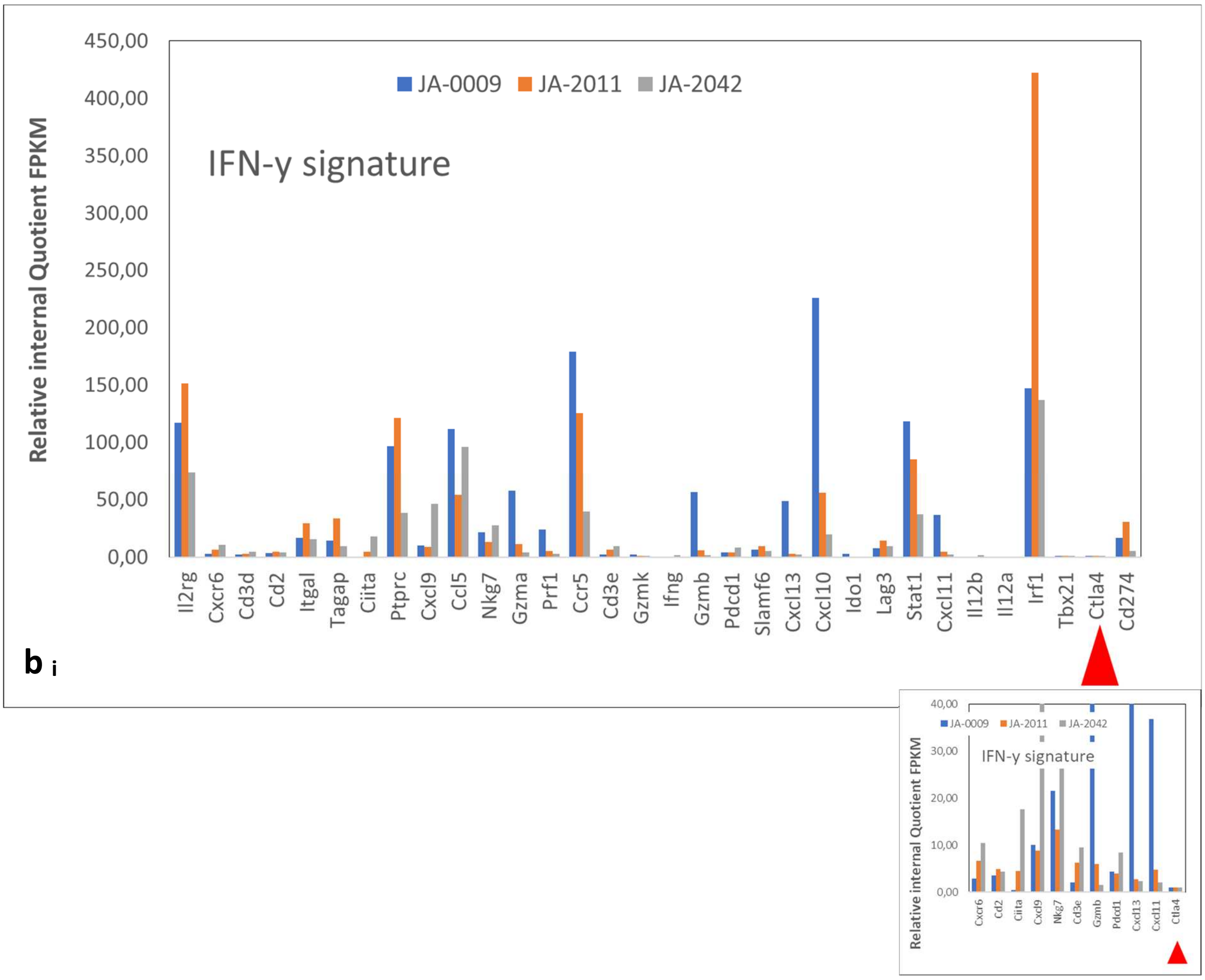

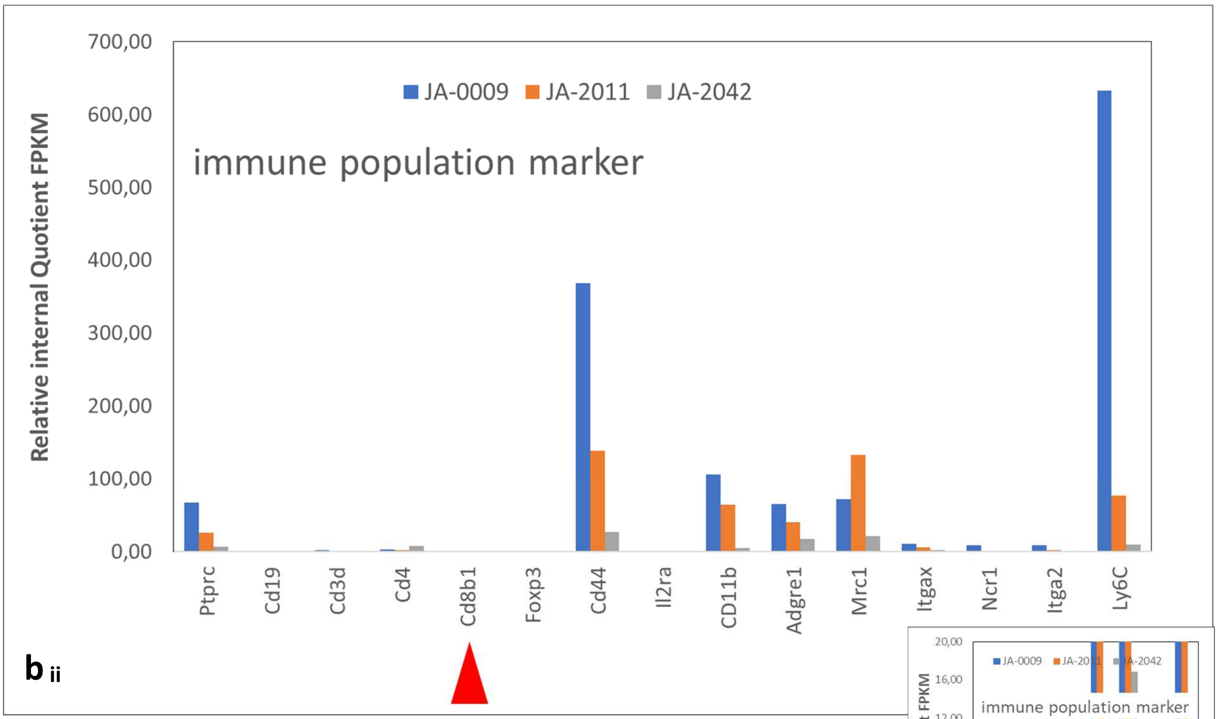

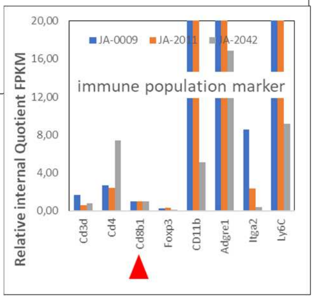

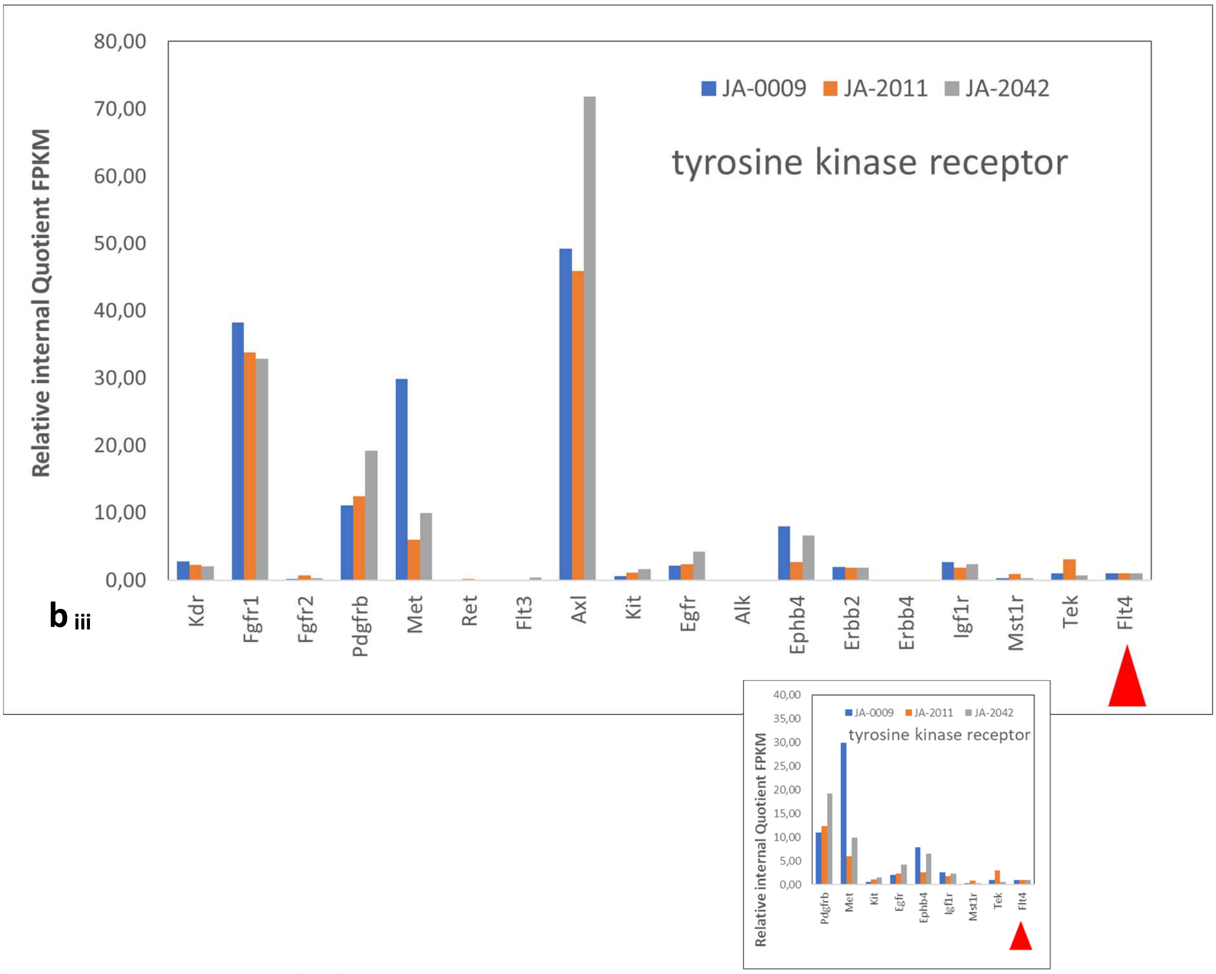

Fig. S1b – sMDI_RNA-Seq: Internal Relative Expression

Graphical presentation of Internal Relative Expression (IRE) of RNA-seq determined FPKM-Values of various genes of three gene families in the three (one sMDI, and two cMDI) MDI models. The IRE gives one an impression on the strength and variabilities of single gene expression within one gene family of the individual MDI models. IRE was calculated as quotient comparing individual gene expression (FPKM) within individual MDI models each, related to the expression of an internal, low FPKM-value reference gene (red triangle). Data are shown as n-fold of expression of respective reference gene. For each gene family one common reference gene, Ctla4 (IFN y signature), Cd8b1 (immune population marker), and Flt4 (tyrosine kinase receptor) was selected within the MDI models. Relative tumor specific gene expression (IRE) in the individual MDI tumors, JA-0009, JA-2011, and JA-2042 shows that in some cases genes, i.e. Fgfr1 (TKR), display a very similar relative expression, whereas in other cases, i.e. Irf1 (IFNGS), Ly6C (IPM), or Met and Axl (TKR), respectively, relative gene expression in the various models differs strikingly. Small inserts show gene expression of selected gene with other y-axis scaling.

1.3 *Doc. S1 - sMDI: sMDI - Histological and Pathological Analysis*

The document summarizes detailed histopathological analysis of primary and derived tumors of various sMDI models inclusive respective large microphotographs

Doc. S1-sMDI

Pathological investigations on index and recipient mice with the present sMDI tumors:

Table of Contents

JA-0011 sMDI: Histiocytic Sarcoma (HS) / Histiocyte-associated Lymphoma (HAL) 14

JA-0013 sMDI: Malignant Lymphoma (ML) 22

JA-0018 sMDI: Malignant Lymphoma (ML) 27

JA-0034 sMDI: Malignant Lymphoma (ML) 29

JA-0021 sMDI: Malignant Lymphoma (ML) 31

JA-0009 sMDI: Adenocarcinoma, anaplastic, invasive 35

JA-0017 sMDI: Adenocarcinoma, complex type 42

JA-0023 sMDI: Adenocarcinoma, mammary gland 46

JA-0032 sMDI: Adenocarcinoma, Lung 56

Literature cited: 58

List of Figures

Figure 1: Macroscopic aspects of the JA-0011 neoplasm index case 14

Figure 2: Index case 011-14, DBA/2N ♀, histiocytic tumor microscopic aspect in liver I 15

Figure 3: Index case 011-14, DBA/2N ♀, histiocytic tumor microscopic aspect in liver II 16

Figure 4: Index case 0011-14; aspects the histiocytic sarcoma in the kidney 17

Figure 5: Index case 011-14, DBA/2N ♀, histiocytic tumor aspect in oviduct. 18

Figure 6: Case 011-14/1298-16, DBA/2N ♀, tumor microscopic aspect in spleen 19

Figure 7: Case 011-14/0052-17, DBA/2N ♀, histiocytic tumor: microscopic aspect in liver 20

Figure 8: Lineage derived tumor 0052-17: aspect of tumor lineage in the spleen 21

Figure 9: Macroscopic aspects of index case of tumor line JA-0013 22

Figure 10: Index case of JA-0013: high magnification view of the neoplastic tissue 23

Figure 11: Lineage case of JA-0013: 0026-17 high magnification view of the neoplastic tissue ... 24

Figure 12: Lineage case of JA-0013: 1562-16 high magnification view of the neoplastic tissue ... 25

Figure 13: Lineage case of JA-0013: 0027-17 medium magnification view of the neoplastic tissue. 26

Figure 14: Macroscopic aspect of JA-0018 (case 18-14) ... 27
Figure 15: Index neoplasm of sMDI JA-0018 (15-14) noted in the lungs of a ♀ CBA/J mouse ... 27
Figure 16: Low, medium and high magnification of 0113-17 malignant lymphoma (ML) derived from index case 0018-14 tumor, subcutis, adipose and mammary ... 28
Figure 17: Macroscopic aspect of **JA-0034** ... 29
Figure 18: Low and high magnification of the malignant lymphoma (ML) index 0034-14 ... 30
Figure 19: Medium magnification of the daughter malignant lymphoma case 1426-17 ... 30
Figure 20: Spontaneous neoplasm study (study #10445): normal skin tissue with a mammary teat, female mouse 0021/14 ... 31
Figure 21: Early malignant lymphoma localization in a lymph node – Index 0021-14 ... 32
Figure 22: Spontaneous neoplasms (study #10445): malignant lymphoma, female SCID *bg/bg* mouse 1571/16 ... 33
Figure 23: Macroscopic aspects of the index tumor **009-14** (index case for lineage JA-0009) ... 35
Figure 24: Medium high magnification of the tumor index case 009-14, with glandular and anaplastic/ spindle shaped differentiation ... 36
Figure 25: High magnification of the tumor index case 009-14, with clear evidence of epidermoid differentiation, suggesting an epidermoid carcinoma ... 37
Figure 26: A moderate high magnification view of 009-14 showing spindle shaped cell differentiation ... 38
Figure 27: Low magnification view of the index neoplasia 009-14 ... 39
Figure 28: Low magnification aspect of the transmitted tumor 1286/16 from index case 09-14 . 40
Figure 29: High magnification aspect of the transmitted tumor 1286/16 ... 41
Figure 30 : Gross aspects of the index neoplasm 0017-14 ... 42
Figure 31: Image ID: 780_#10445_tu01_0017-14_0017-14_HE_10x_1-AdK--mixed tumor-index-case, HE stain, original magnification: 100x ... 43
Figure 32: Image ID: 780_#10445_tu_0017-14_1234-16_HE_20x_2-AdK-wKeratinization-mixed tumor-complex-myoepiith-epiith2lmesenchT-wC, HE stain, original magnification: 200x ... 44
Figure 33: Image ID: 780_#10445_tu01_0096-17_0017-14_HE_20x_2-AdK--mixed tumor- trab-K, HE stain, original magnification: 200x. ... 45
Figure 34: Low (left) and mid (right) magnification views of the index tumor from the kidney.... 47
Figure 35: High magnification view of the index renal tumor ... 47
Figure 36: Index tumor of the subcutaneous tumor: microscopic morphological aspects ... 48
Figure 37: Lineage tumor aspect in subcutaneous tissue: example of **0005-17** ... 49
Figure 38: Case 0005-17: High magnification reveals the tubulopapillary structure with necrotic cells in lumens and supportive stroma ... 50
Figure 39: Lineage tumor aspect in kidney: example of **0001-17** ... 51
Figure 40: Lineage tumor aspect in subcutaneous tissue: example of **1620-16** ... 52
Figure 41: Spontaneous neoplasms (study #10445): papillary cystic adenocarcinoma, female mouse 0547/17, hematoxylin and eosin stain, subcutis ... 53

Figure 42: Spontaneous neoplasms (study #10445): papillary cystic adenocarcinoma, female mouse 0544/17, skin 54
Figure 43: Spontaneous neoplasms (study #10445): papillary adenocarcinoma, lung, male mouse 0032/14 56
Figure 44: Spontaneous neoplasms (study #10445): adenocarcinoma, solid, clear cells, subcutis, male mouse 0055/17 57
Figure 45: Low magnification of tumor aspect overview and high view detail (t**humbnails**) 58

JA-0011 sMDI: Histiocytic Sarcoma (HS) / Histiocyte-associated Lymphoma (HAL)

Index tumor **JA-0011** (Fig. 2) was classified as a histiocytic sarcoma (HS) or histiocyte-associated lymphoma (HAL), as localized in several tissues (liver, gut, kidney, spleen – see Figure 1 to Figure 5), with both histiocytic and lymphocytic components. Re-transplanted subcutaneous tumors, **0339-17, 4007-16, or 0057-17** also invaded spleens e.g. **1298-16** (Figure 6) and/or livers e.g. **0052-17** (Figure 7) of recipient mice, with either lymphoblastic (e.g. **1298-16**) or histiocytic predominant differentiation . These features are topographical and histological characteristics of these hemolymphopoietic neoplasms.

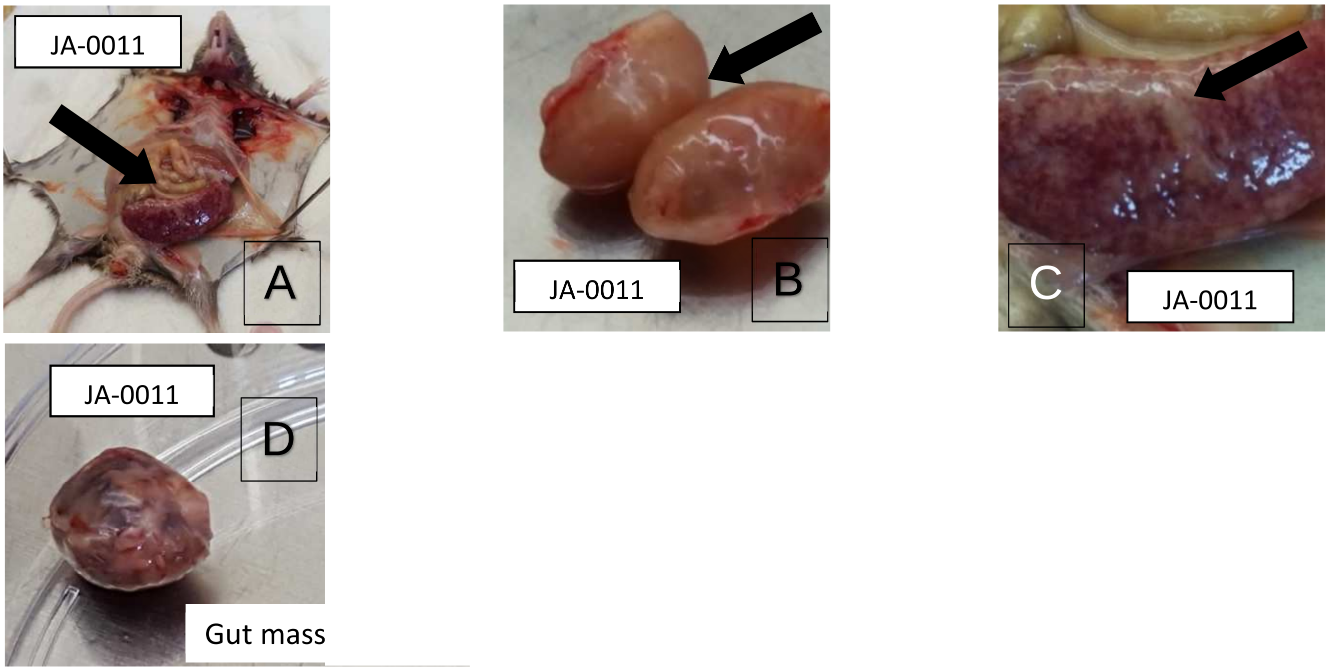

Figure 1: Macroscopic aspects of the **JA-0011** neoplasm index case

A: the spleen is markedly enlarged (**arrow**) and the liver pale yellow and enlarged. **B**: Kidneys are also paler than normal and enlarged; one kidney has segmental more important pale discoloration (arrow) **C:** Close up view of the spleen (see A), showing the white mottling **(arrow**) discoloration. **D:** mass noted in the abdominal cavity, of obvious tumoural origin, but without identifiable tissue of origin.

Diagnosis: Neoplasm of Hemolymphopoietic origin: most compatible with: histiocytic sarcoma (HS) or histiocyte-associated lymphoma (HAL)

Main features: multicentric tumor composed of sheets and bundles of histiocytic cells in various tissues, including, but not limited to spleen, liver, kidney, oviducts, with:

- presence of syncitial multinucleate cells (Hallmark of the neoplasm)

- significant infiltration of neoplastic cells by polymorphonuclear leukocytes (neutrophils, eosinophils), often immature (band granulocytes) erythropoietic cells and lymphoid cells

- presence of inflammation

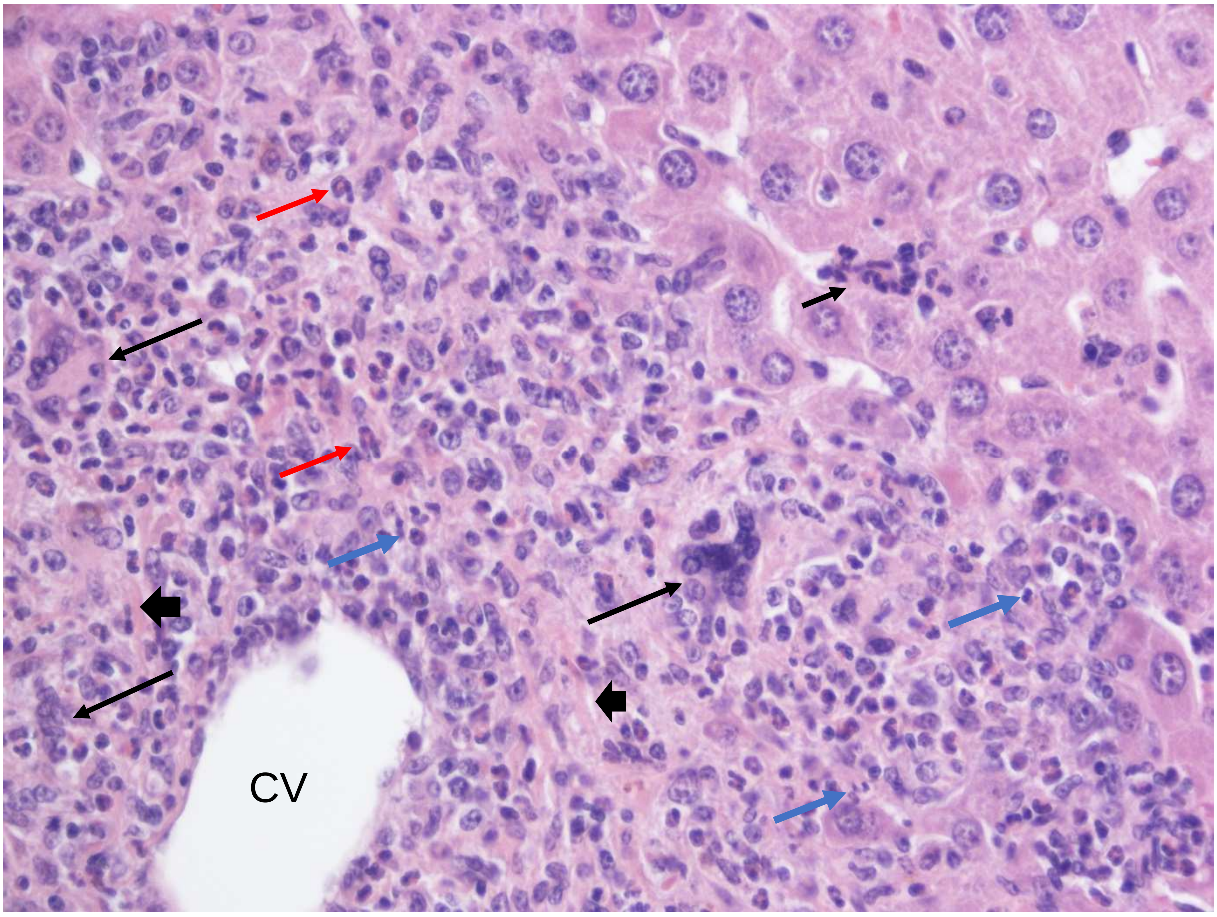

Figure 2: Index case 011-14, DBA/2N ♀, histiocytic tumor microscopic aspect in liver I

The malignant proliferation is characterized by sheets and bundles of pale macrophage-like histiocytic cells, which progressively compress, invade and replace the hepatic parenchyma and by the presence of many multinucleate giant cells (**long black arrows**). There is also relatively significant associated inflammation and/or recruited mixed leukocytes, a majority of which is characterized by histiocytes/macrophages infiltrates cytologically within normal limits, a few of which in a necrotic/degenerated form (small black arrow in the upper left quadrant area). Accompanying this histiocytic and macrophagic proliferation, are significant infiltrates of polymorphonuclear (PMN) leukocytes, associating both neutrophils (**blue arrows**) and some eosinophils (**red arrows**). On this particular high magnification view centered on a central vein (**CV**), the neoplastic proliferation (cell type) is not particularly obvious. The neoplastic process is chronic (probably relatively indolent/slow growing neoplasm), as judged by the associated florid fibrosis accompanying the predominantly granulomatous-like inflammation (see arrowhead ◀).

Index case: mouse ID number 011-14, strain DBA/2N ♀, HE stain, original magnification: 400x.

Morphological diagnosis: Histiocytic Sarcoma/ Histiocyte-associated lymphoma.

Abbreviation: HS/HAL

Image ID: 780_#10445_liver_0011-14_HE_40x_1_syncytial-giant-cell_inflammation

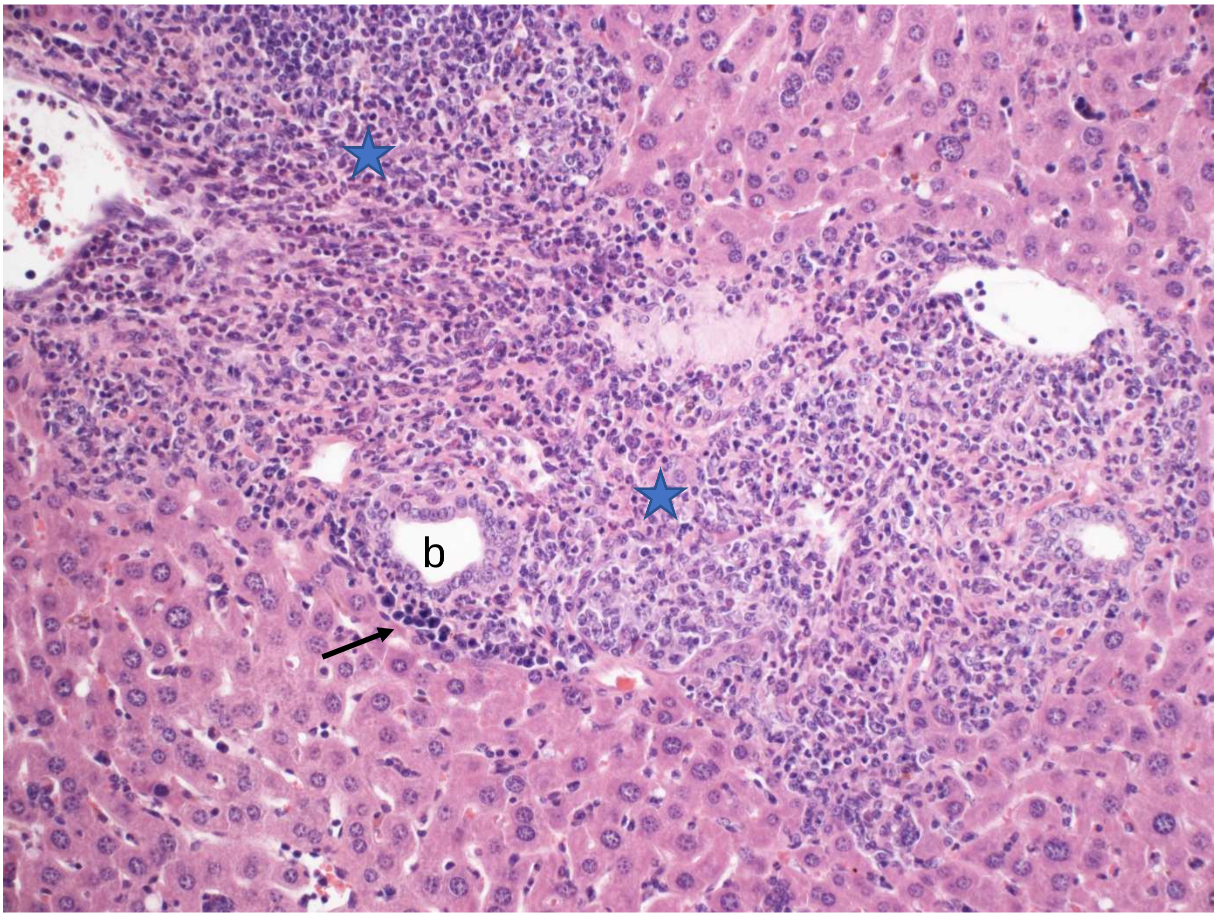

Figure 3: Index case 011-14, DBA/2N ♀, histiocytic tumor microscopic aspect in liver II

Index case: mouse ID number 011-14, strain DBA/2N ♀ same case as in Figure 2; HE stain, original magnification 200x.

The portal areas of the liver have diffuse marked mononuclear and histiocytic diffuse infiltration of the connective tissue (blue stars), with a significant population of large blastic cells and cells most compatible morphologically with small lymphocytes. The mixed inflammatory cell infiltration is similar in intensity and type to Figure 2. There is also associated erythropoiesis (extramedullary hematopoiesis – **black arrow**). b = small bile duct.

Morphological diagnosis: Histiocytic Sarcoma/ Histiocyte-associated lymphoma.

Abbreviation: HS/HAL

Image ID: 780_#10445_liver_0011-14_Liver_HE_20x_1_HS_pport-area.

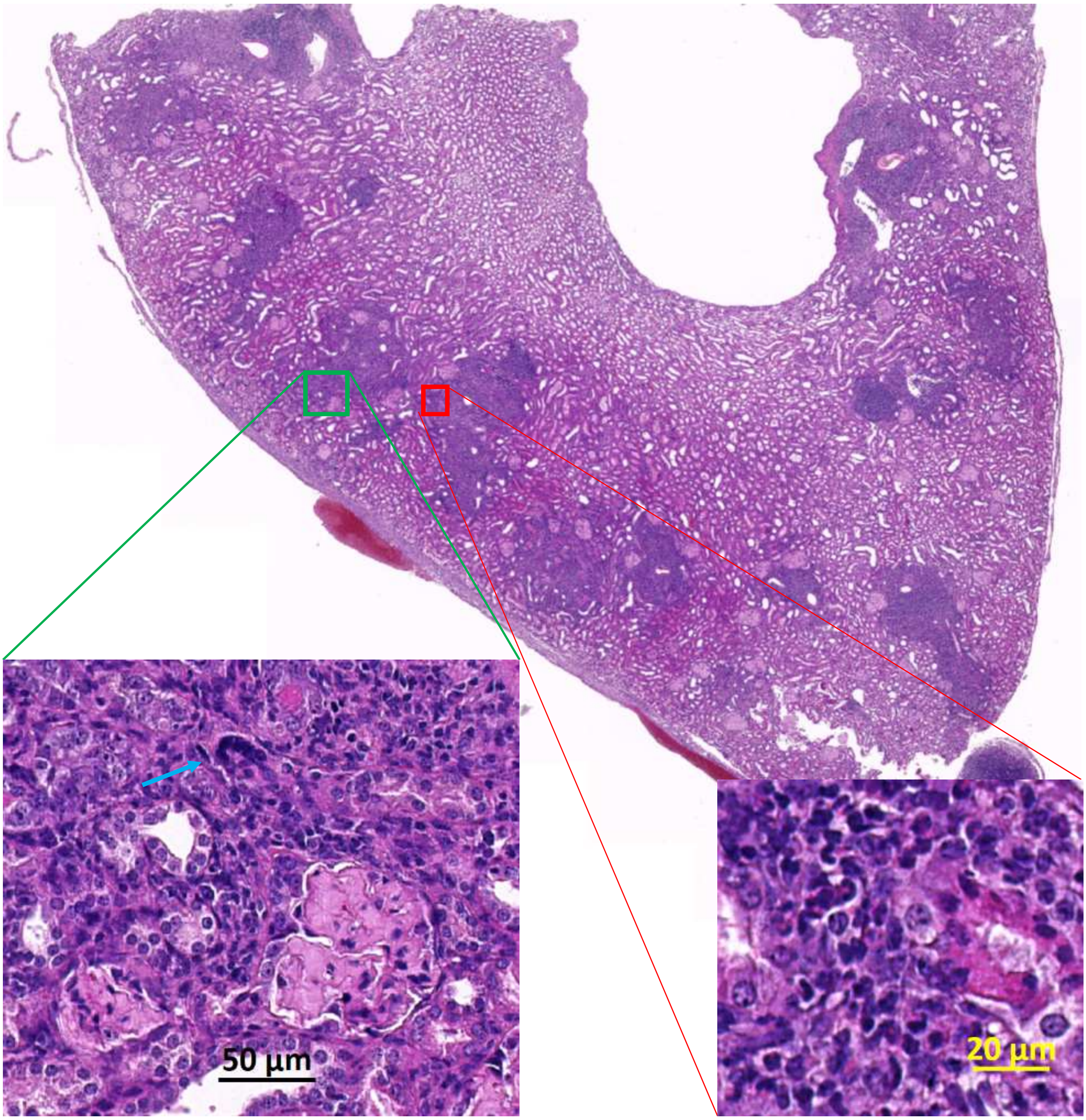

Figure 4: Index case 0011-14; aspects the histiocytic sarcoma in the kidney

Similar foci as in the liver (indicating secondary localizations) are scattered in the cortex of the kidney cortew, with glomerular hyalinosis (left green inset) and features of infiltrating cells similar to the liver: presence of multi-nucleate giant cells (blue arrow), and various proportions of histiocytes, granulocytes and small lymphocytes (right small red inset) with evidence of associate tubular degeneration and regeneration.

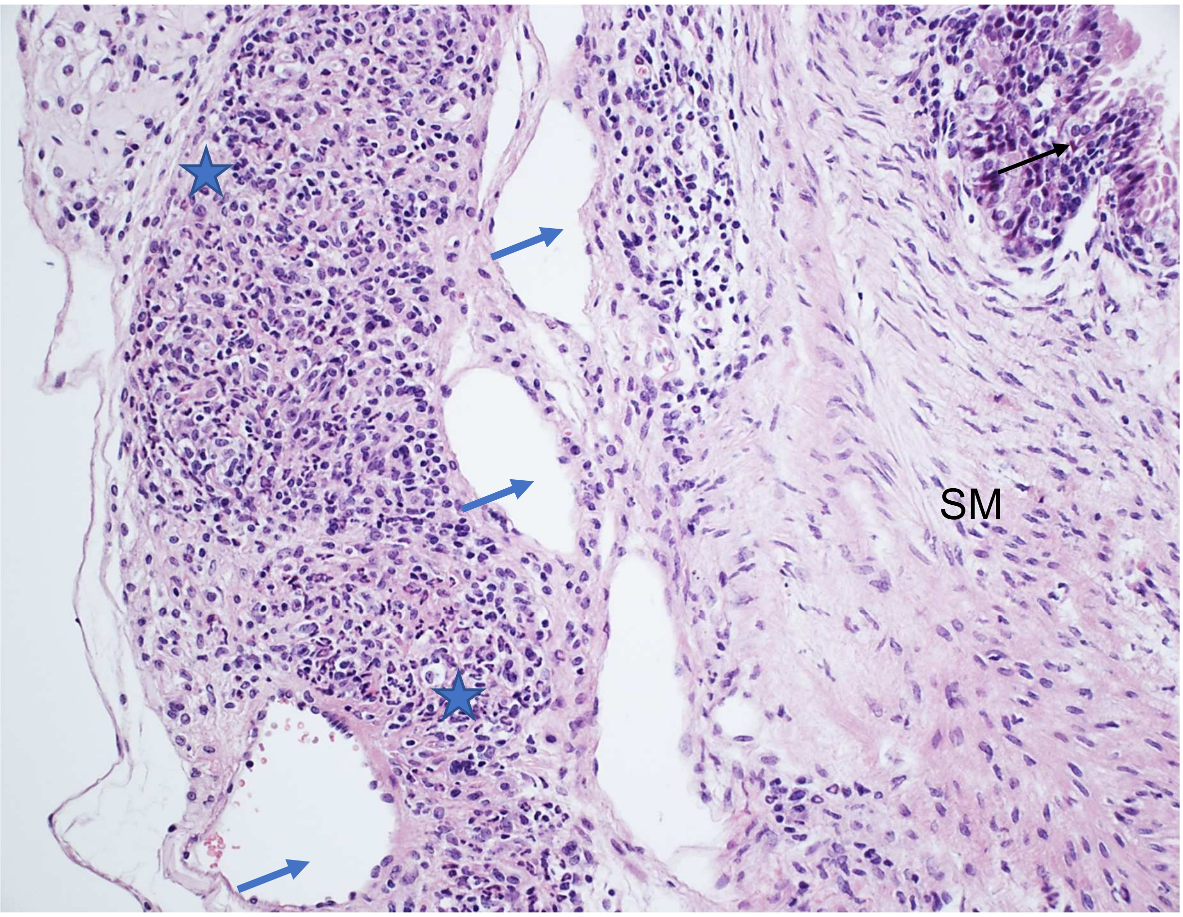

Figure 5: Index case 011-14, DBA/2N ♀, histiocytic tumor aspect in oviduct.

Index case: mouse ID number 011-14, strain DBA/2N ♀, HE stain, original magnification: 400x

Cavitary organ composed of a smooth muscle wall and ciliated pseudostratified epithelium (**black arrow**): Female reproductive tract, most compatible with oviduct.

A proliferative infiltrate similar to liver (see Figure 2 and Figure 3) and kidney is noted (blue stars), markedly thickening the serosal surface of a cavitary organ most compatible with oviduct adjacent to ovary. This proliferative tissue induces marked dilatation of blood and lymph vessels (**blue arrows**). SM = smooth muscle layer of the oviduct.

Consequently, it may be surmised that the intestinal mass was most probably a heavily invaded ovary (see Figure 1 panel D). Ovary is a frequent primary or secondary localization of histiocytic sarcoma (see Reference 1)

Morphological diagnosis: Histiocytic Sarcoma/ Histiocyte-associated lymphoma.

Abbreviation: HS/HAL

Image ID: 780_#10445_ORG_0011-14_Darm_HE_40x_1_HS

Isografts – example 1: 1298-16

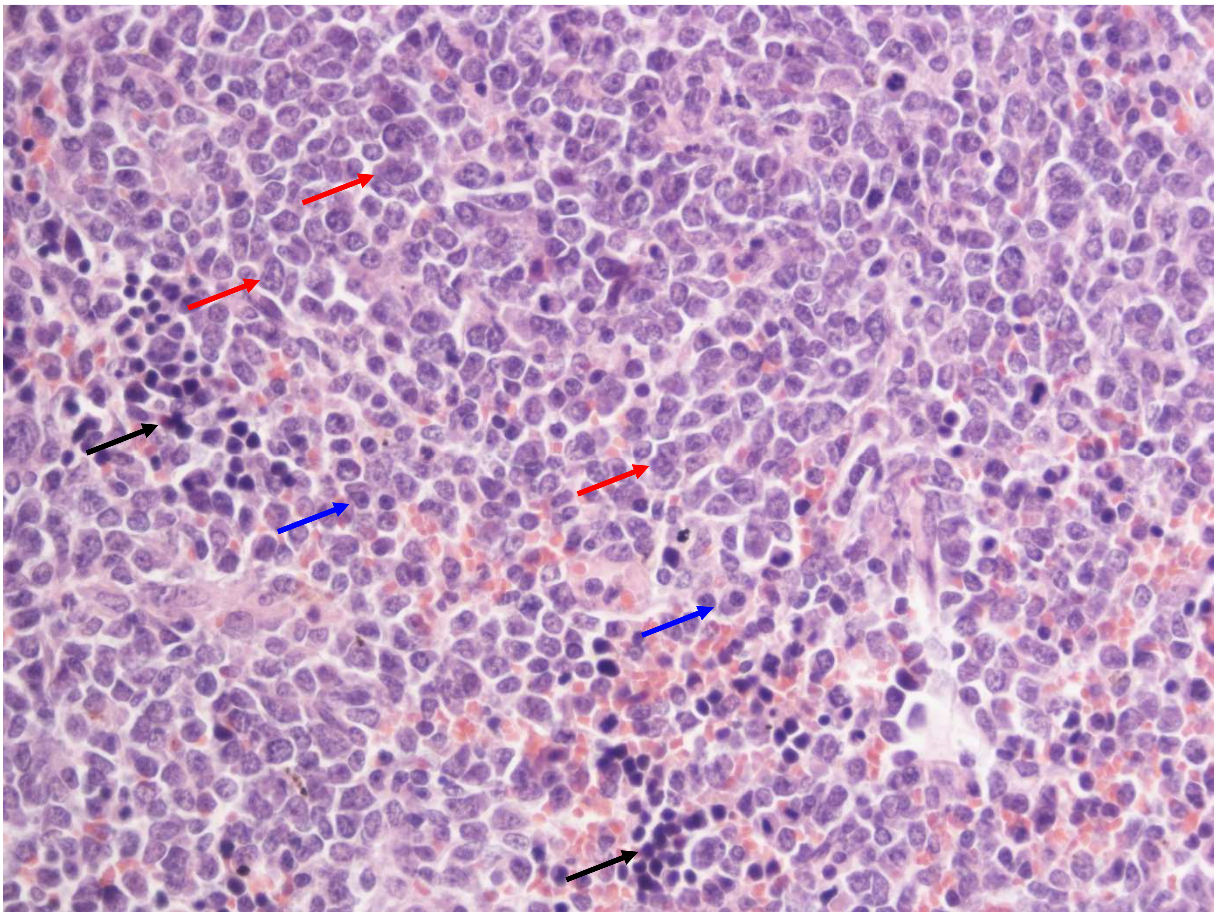

Figure 6: Case 011-14/1298-16, DBA/2N ♀, tumor microscopic aspect in spleen

Mouse ID #1298-16 **spleen tissue**; from index case: mouse ID number 011-14, strain DBA/2N ♀. HE stain, original magnification: 400x.

Diffusely (almost entirely) erasing the structure of the white and red pulp of the spleen are diffuse relatively homogenous sheets (see **thumbnails** below) of immature large “blastic” mononuclear cells most compatible morphologically with immature lymphoblasts (**red arrows**). Some of the differentiated cells have morphology suggesting plasma cells (blue arrows). There is also extramedullary erythropoiesis (**black arrows**). In this case, early infiltration in the liver features similar cells as in the spleen, with few inflammatory cells (data not shown).

This demonstrate the lymphoma potential of this particular cell lineage.

This phenotypic variability would warrant further molecular pathology characterization by immunohistochemistry and in situ hybridization when re-used in future studies

Morphological diagnosis: Malignant lymphoma.

Image ID: 780_#10445_mz_0011-14_1298-16_HE_40x_1_lymphoma

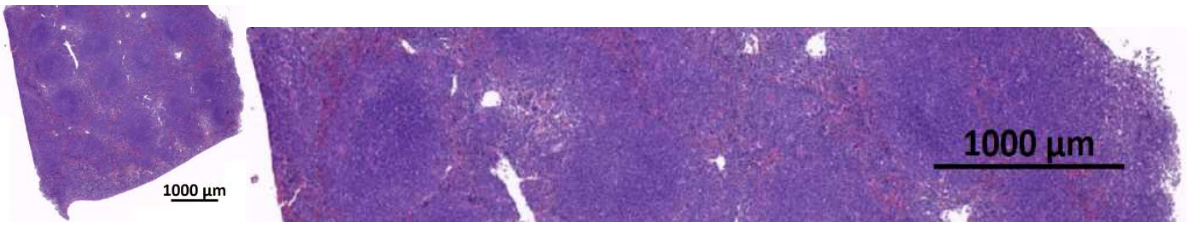

Isografts – example 2: 0052-17

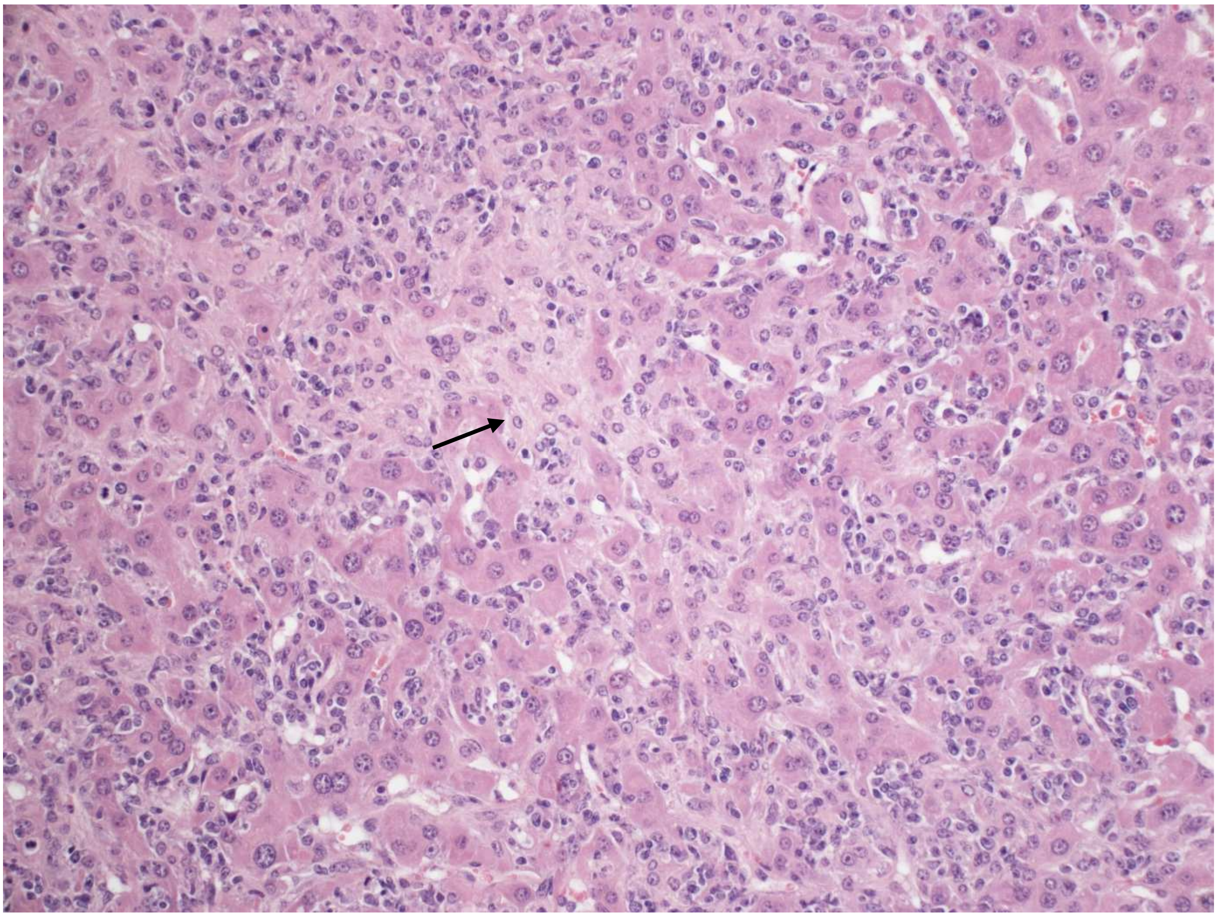

Figure 7: Case 011-14/0052-17, DBA/2N ♀, histiocytic tumor: microscopic aspect in liver

Mouse ID #0052-17; from index case: mouse ID number 011-14, strain DBA/2N ♀. HE stain, original magnification: 200x.

Notice the almost pure central proliferation of histiocytic cells widely thickening the sinusoids (**black arrow**). Histiocytes have homogenous pale nuclei and amorphous pale pink abundant cytoplasm with indistinct boundaries and low/absent anaplastic or pleomorphic features, making them as typical histiocytes/macrophages. Furthermore, these cells closely resemble typical hypertrophic Kupffer cells, replacing the normal looking fenestrated endothelial covering of sinusoids. Other cells at the vicinity are of various types, including small, mainly medium-sized and few large lymphocytes.

This phenotypic variability of this lineage JA-0011 would warrant further molecular pathology characterization by immunohistochemistry and in situ hybridization when re-used in future studies

Morphological diagnosis: Histiocytic Sarcoma/ Histiocyte-associated lymphoma.

Abbreviation: HS/HAL

Source file: Image ID: 780_#10445_leb_0011-14_0052-17_HE_20x_1_HS

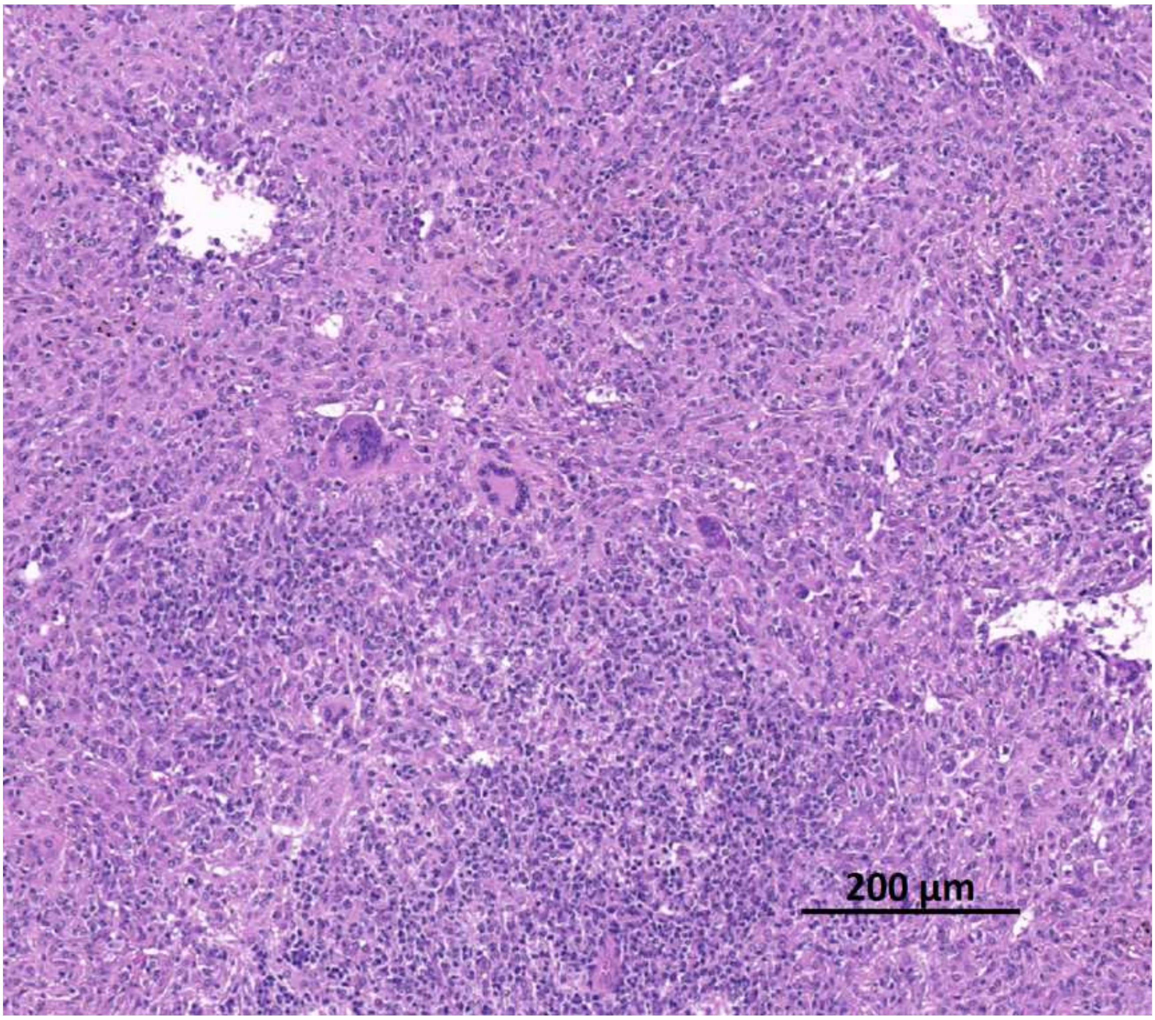

Figure 8: Lineage derived tumor 0052-17: aspect of tumor lineage in the spleen

The spleen is grossly enlarged (**see thumbnail below**) and the red pulp is diffusely invaded with sheets of histiocytic and cells with occasional to numerous giant multinucleate cells. The white pulp is also invaded and partially erased by the neoplastic process. Contrast with case in Figure 6, where a malignant lymphoma is present in the spleen of case 1298-16.

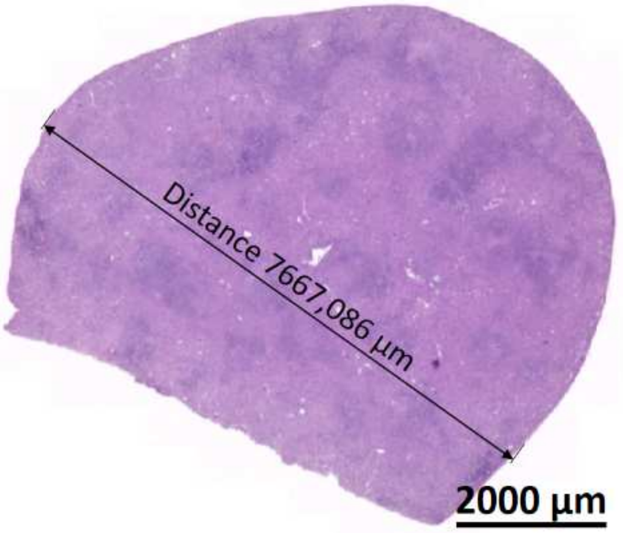

JA-0013 sMDI: Malignant Lymphoma (ML)

The phenotype of tumor **JA-0013/** as exemplified by specimens **0013-14** (index case) and mice **1562-16**, **0026-17**, **0027-17** and **0028-17** (Fig. 2) was most consistent with malignant lymphoma, which was probably of B-cell lineage, as judged by the presence of infiltration of blastic cells with immunoblastic or plasmacytic differentiation (Figure 10) plasma and Mott cells (plasma cells containing Russell bodies) and the presence of large histiocytoid cells (see Figure 10). It had a remarkable phenotypic stability (see Figure 11 and Figure 12). One daughter re-transplanted tumor studied microscopically, **0027-17**, had an intriguing finding, non-neoplastic giant multinucleate giant cells, leading to suspect a possible complication with an opportunistic organism (see Figure 13).

Gross aspects were also typical of malignant lymphoma (see Figure 9)

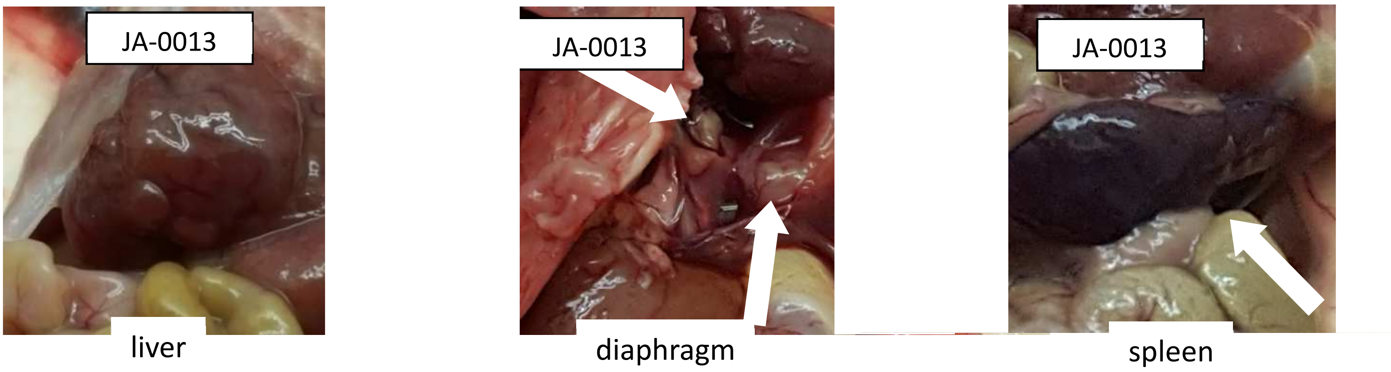

Figure 9: Macroscopic aspects of index case of tumor line **JA-0013**

There is markedly enlarged liver, 2 masses in pulmonary hilus in the thorax (behind the diaphragm) and a markedly enlarged spleen

Primary differential diagnosis: Lymphoma, lymphoblastic (with histiocytoid large cells) - most likely lineage origin: B cells

Immunohistochemistry would be useful for precise lineage definition.

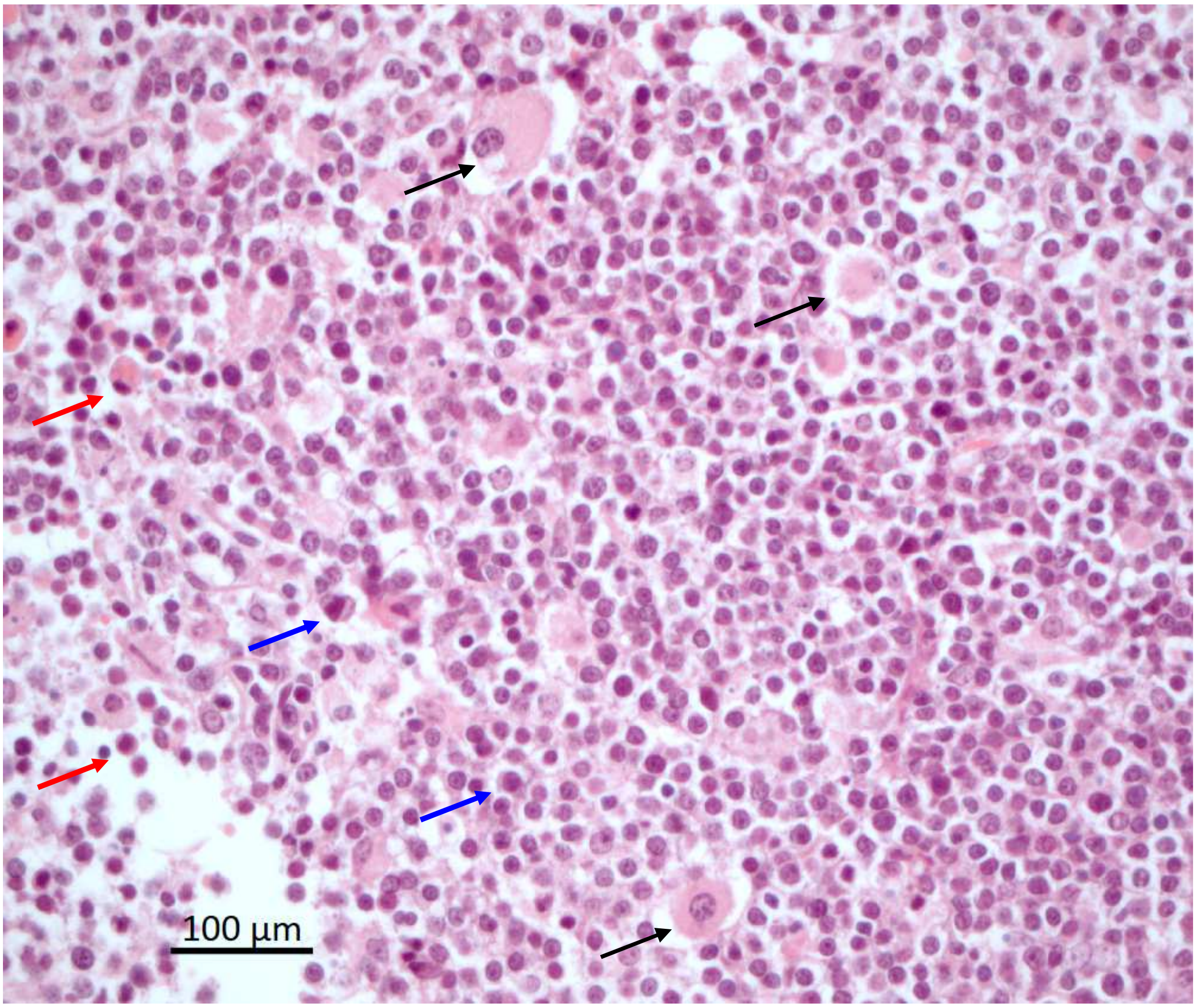

Figure 10: Index case of JA-0013: high magnification view of the neoplastic tissue

This specimen from pulmonary hilus/diaphragmatic masses exhibits sheets of malignant mid-sized blastic lymphocytes with presence of large, 50-70 µm diameter atypical cells with a pale glassy eosinophilic cytoplasm (**black arrows**). The sheets are irregularly infiltrated with smaller cells with a stem cell morphology, which could be of neoplastic origin or myeloid in origin (related to a response to anemia) and with plasmacytic cells, either neoplastic like (blue arrows) suggesting plasmacytic differentiation, or typical plasma cells with Russel bodies (Mott cells – see red arrows). Tumor infiltrating lymphocytes and other normal host inflammatory cells cannot be appraised without special molecular pathology techniques based on HE-stained sections and morphology alone.

Hematoxylin eosin stain, 400 x original magnification.

Morphological diagnosis: Malignant lymphoma.

Abbreviation: ML

Source file: 2019-01-24 19_40_09-TPL780-#10445-dia-0013-14_0013-14_1_400x_2-index-lymphoma.czi - ZEN 2.3 lite

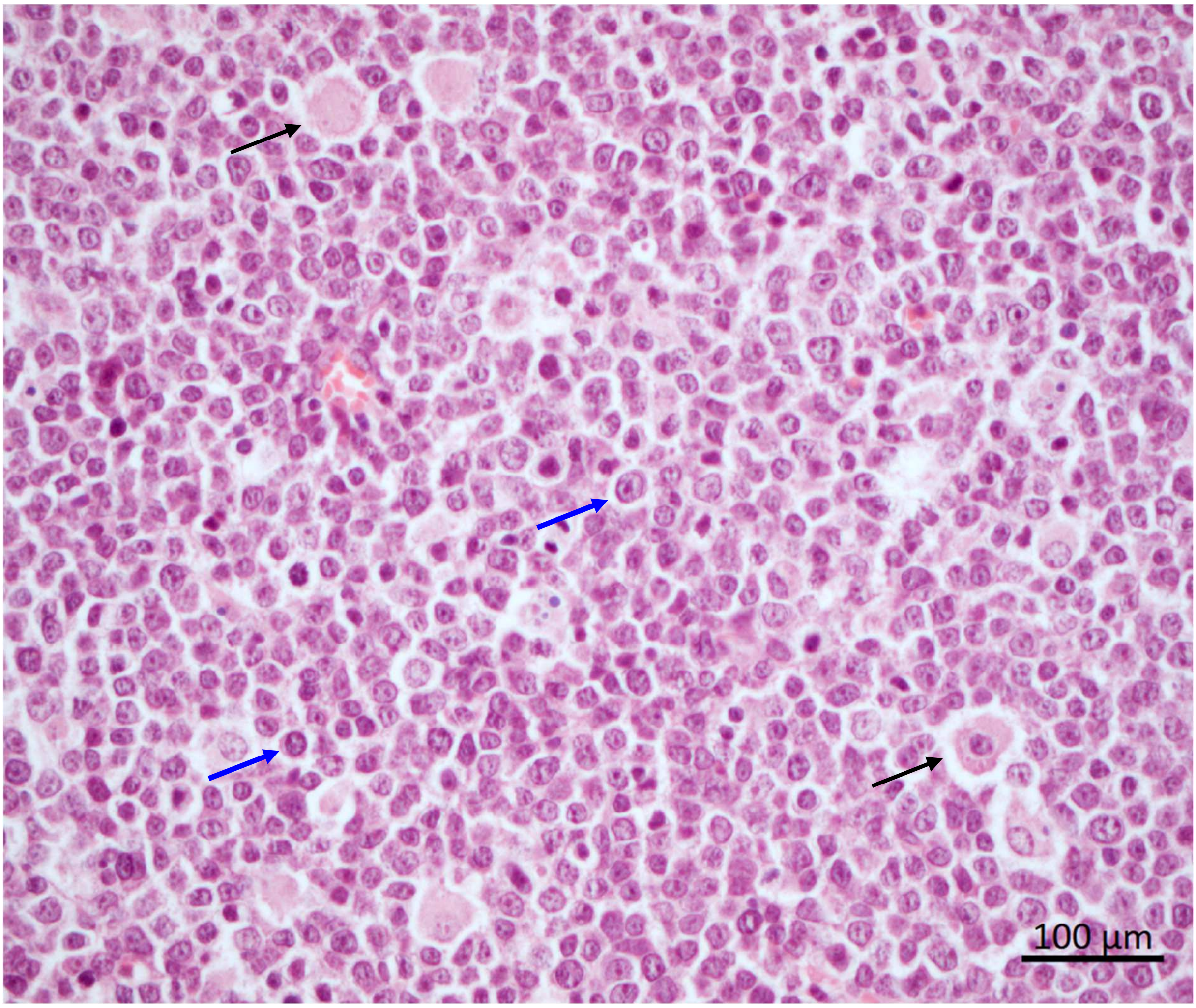

Figure 11: Lineage case of JA-0013: 0026-17 high magnification view of the neoplastic tissue

This tumor is derived from index case: 0013-14

When comparing to the index case, there is remarkable phenotypic stability in the tissue of this particular mouse, with the conservation of the large histiocytoid cells (**black arrows**) and the plasmacytic differentiation, with scant eccentric cytoplasm (blue arrows). All these simple morphologic features are most typical of B-cell differentiation and are indicating phenotypic stability, at least morphologically. B cell or T cell may be verified by immunohistochemistry.

Morphological diagnosis: Malignant lymphoma.

Abbreviation: ML

Cancer lineage: lymphocytes

Source file: 019-01-24 19_40_09-TPL780-#10445-dia-0013-14_0013-14_1_400x_2-index-lymphoma.czi - ZEN 2.3 lite.png

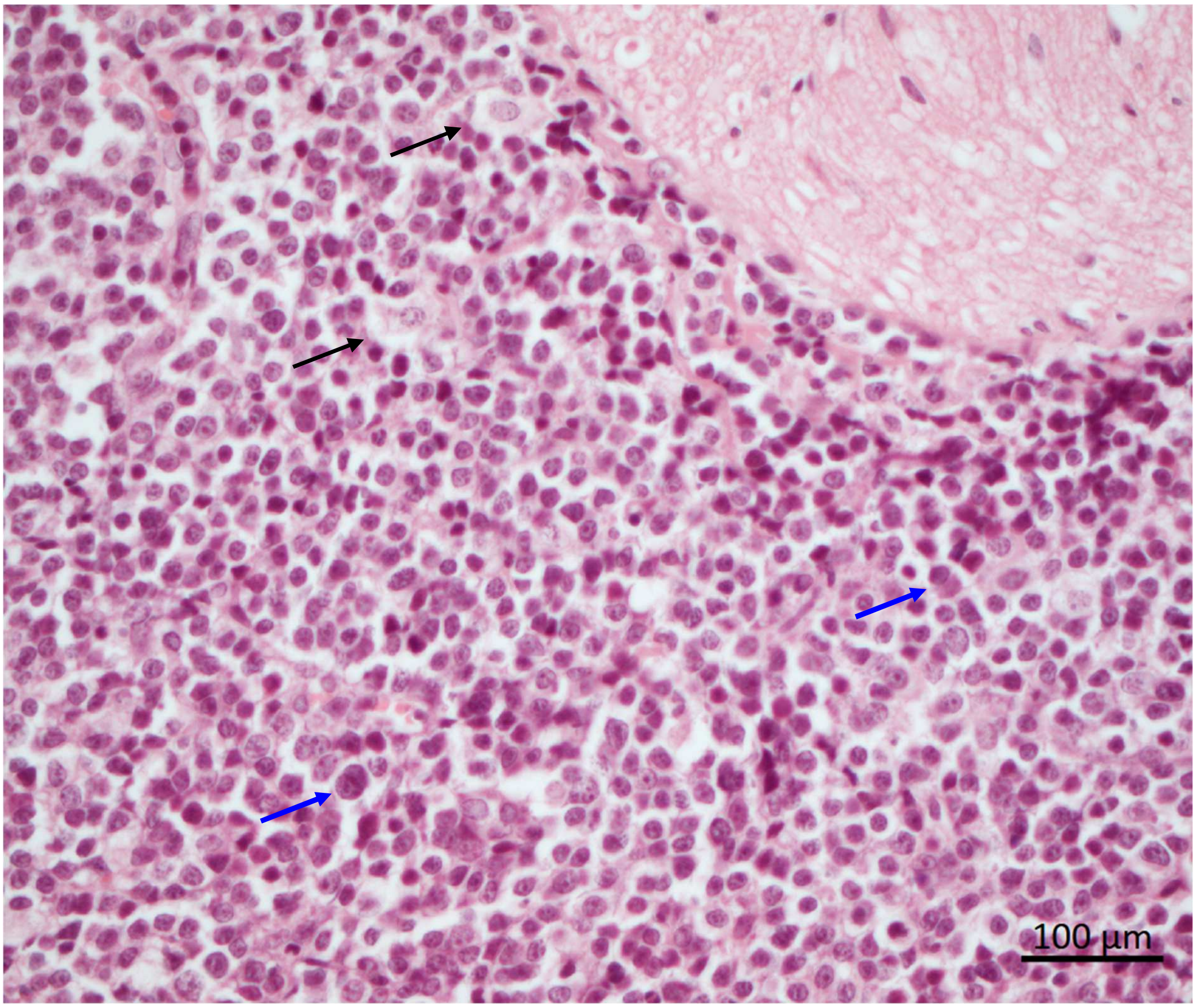

Figure 12: Lineage case of JA-0013: 1562-16 high magnification view of the neoplastic tissue

This tumor is derived from index case: 0013-14

When comparing to the index case, there is remarkable phenotypic stability in the tissue of this particular mouse, with the conservation of the large histiocytoid cells (**black arrows**) and the plasmacytic differentiation, with scant eccentric cytoplasm (blue arrows). All these simple morphologic features are most typical of B-cell differentiation and are indicating phenotypic stability, at least morphologically.

Morphological diagnosis: Malignant lymphoma.

Abbreviation: ML

Cancer lineage: lymphocytes

Source file: 019-01-24 20_31_32-TPL780-#10445-dia-0013-14_1562-16_1_400x_2-lymphoma-with large-histiocytic-like-.png

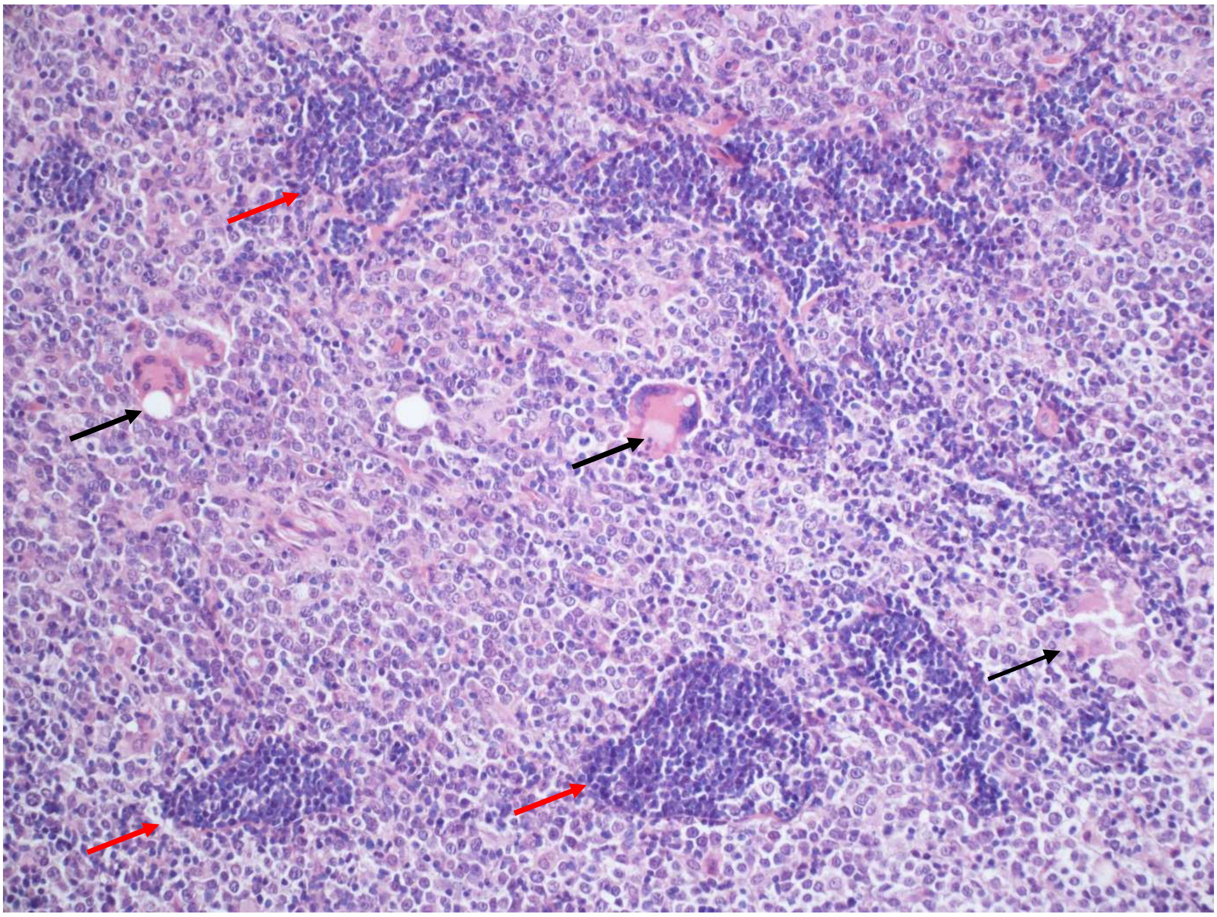

Figure 13: Lineage case of JA-0013: 0027-17 medium magnification view of the neoplastic tissue

Mouse ID #0027-17; from index case: mouse ID number 013-14, ♀ strain C57BL/6 albino.

HE stain, original magnification: 200x.

Tissue of origin: very likely lymph node (presence of lymphatic vessels – not present in the field above) and lymphatic sinuses filled with small lymphocytes (see red arrows – architecture partially preserved)

There is partial erasing of lymphoid architecture by homogenous sheets of round cells most compatible with proliferating (blastic) lymphocytes and scattered rare multinucleate giant cells (MGCs) that have large empty vacuoles or vacuoles containing pale amorphous pale gray material (**arrows**) and are not considered to be neoplastic.

This finding of non-neoplastic giant multinucleate giant cells is intriguing, and leads to suspect a possible complication with an opportunistic organism.

B cell markers would be useful to characterize the proliferation.

Morphological diagnosis: Malignant lymphoma.

Abbreviation: ML

Image ID: 780_#10445_tu_0013-14_**0027-17**_1_HE_20x_1-Lymphoma-with-non-neoMGCs

JA-0018 sMDI: Malignant Lymphoma (ML)

CBA/J ♀ derived tumor **JA-0018/ 0113-17**

Figure 14: Macroscopic aspect of **JA-0018** (case 18-14)

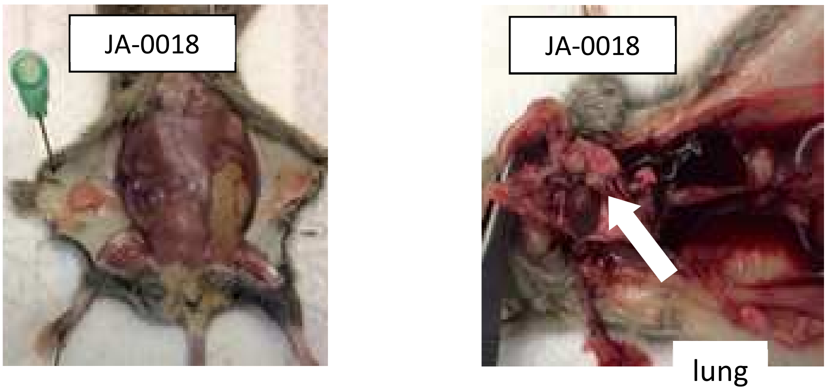

Malignant lymphoma with small lymphocytes – original localization studied: lung

Figure 15: Index neoplasm of sMDI JA-0018 (15-14) noted in the lungs of a ♀ CBA/J mouse

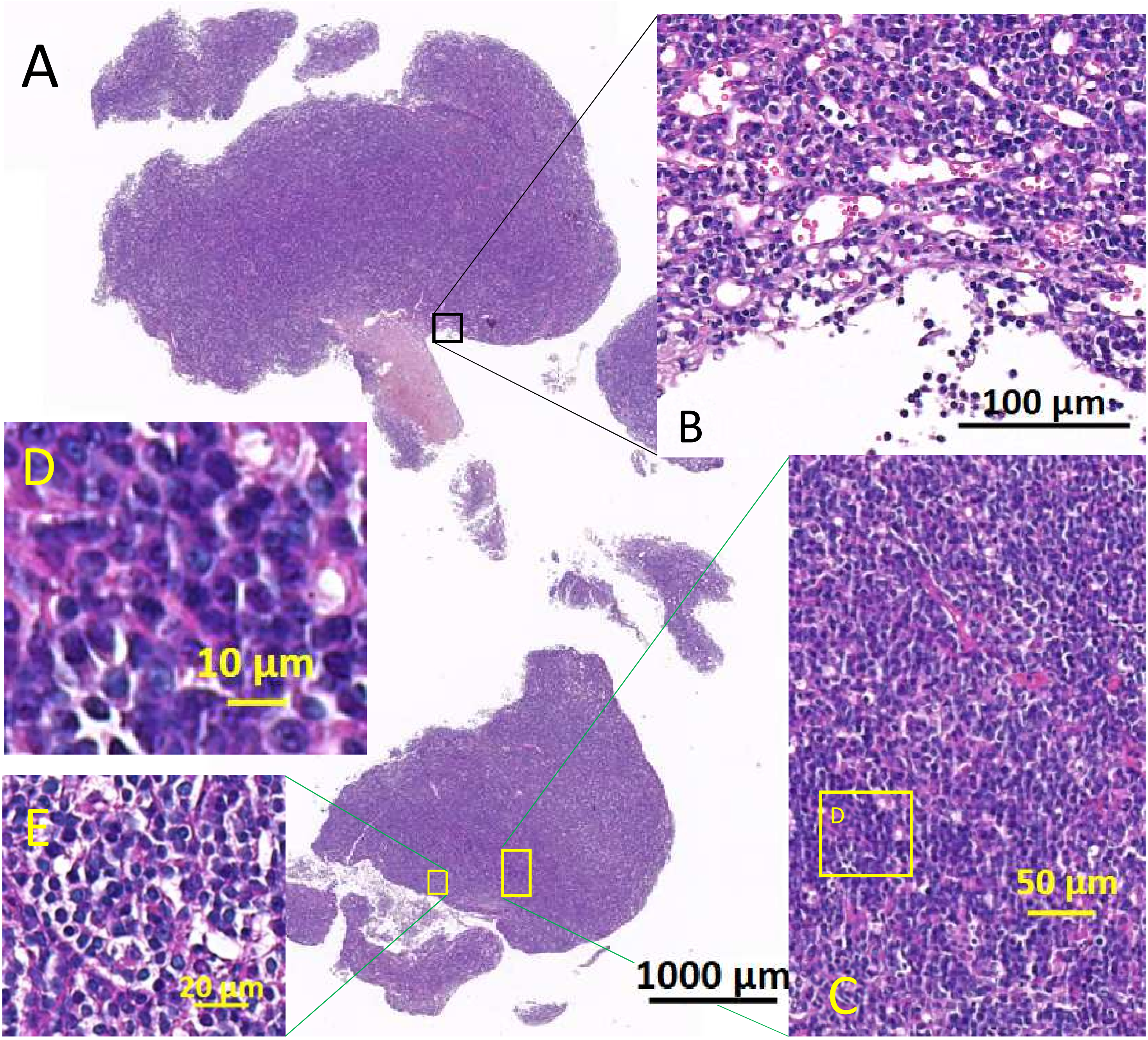

Legend of Figure 15 in **previous page**:

A: Low magnification fragments of the pulmonary neoplasm, exhibiting homogenous deeply basophilic sheets of neoplastic tissue that almost completely the pre-existing morphology of the lung and pleura.

B: This view adjacent to the pleura and mesothelial lining (**black arrow**) shows non-coalescing infiltration with small lymphocytes, with partial preservation of architecture.

C, D, E: medium (C) and high (D, E) magnifications of the neoplasm with small to medium-sized 5-8 µm lymphocytes with pseudo cord (packet) formation (white arrows) in preexisting pulmonary structures, notably pre-existing alveoli (E), mimicking a neuro-endocrine tumor.

Source file: TPL811_17_HE_11975_18_14_10_4_17_Lu

Figure 16: Low, medium and high magnification of 0113-17 malignant lymphoma (ML) derived from index case 0018-14 tumor, subcutis, adipose and mammary

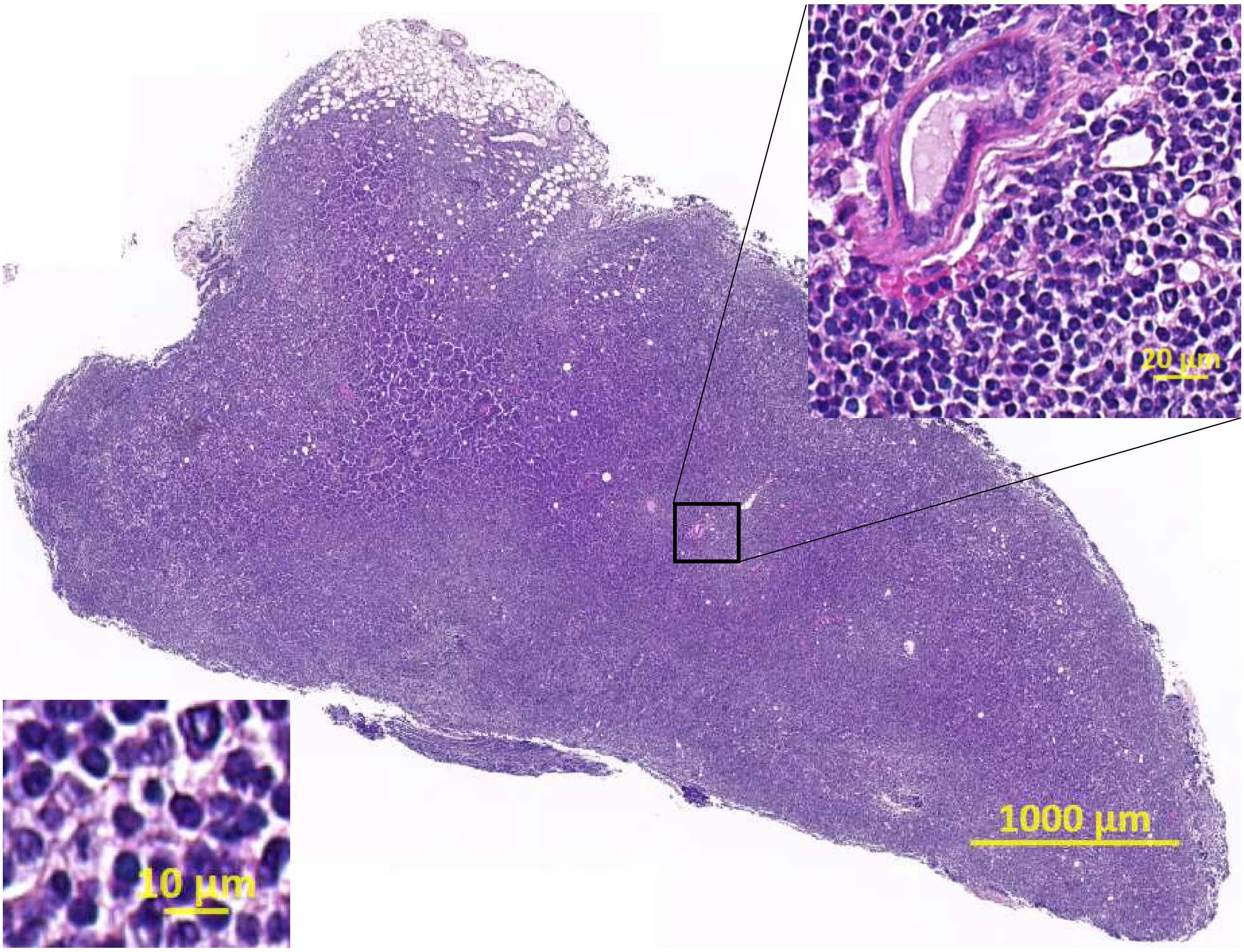

CBA/J ♀: The proliferation is composed of homogenous sheets of small (~ 5 µm in dimeter) to mid-sized (~ 5-8 µm) well differentiated lymphocytes, which are diffusely invading into the subcutis, isolating small ducts and ductules of the mammary gland (see exocrine gland profile of upper right inset). Immunophenotyping would be desirable if this sMDI would be further used as a tumor model.

Source file: TPL811_17_HE_10445_Tu_0113_17.czi

JA-0034 sMDI: Malignant Lymphoma (ML)

In index case **JA-0034**/**0034-14 index case** with **1426-17 0036-17** and **0042-17** as examples of subsequent tumors are all phenotypically stable lymphoma. These lymphoma cells also are characterized by small cell morphology

no *in situ* Foto

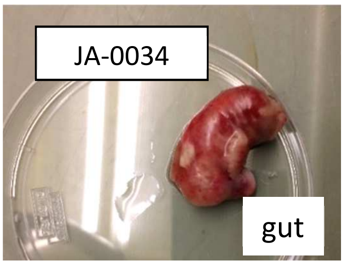

Figure 17: Macroscopic aspect of **JA-0034**

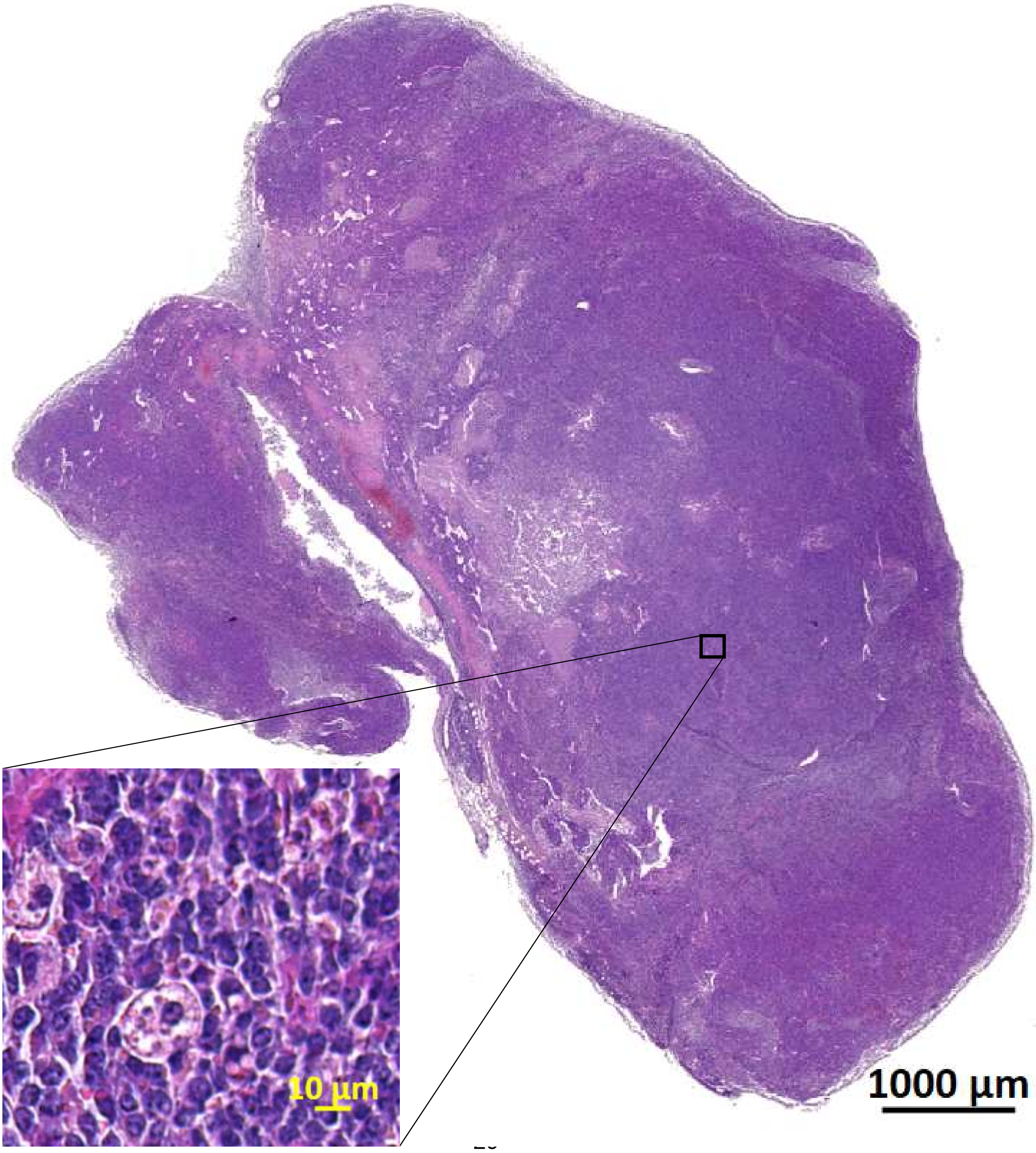

Figure 18: Low and high magnification of the malignant lymphoma (ML) index 0034-14

In previous page: The proliferation is composed of homogenous sheets of small (~ 5 µm in dimeter) to mid-sized (~ 7 µm) well differentiated lymphocytes, which are diffusely invading into the subcutis or a pre-existing lymph node. **Inset:** cells are 7-8 µm and there are also evidence of macrophages with phagocytized apoptotic debris ("starry sky" aspect)..

Morphological diagnosis: Malignant lymphoma, small cells, intestine

Source files:

2019-01-24 12_09_36-#10445darm_34-14_34-14.czi - ZEN 2.3 lite.png

2019-01-24 12_12_07-#10445darm_34-14_34-14.czi - ZEN 2.3 lite.png

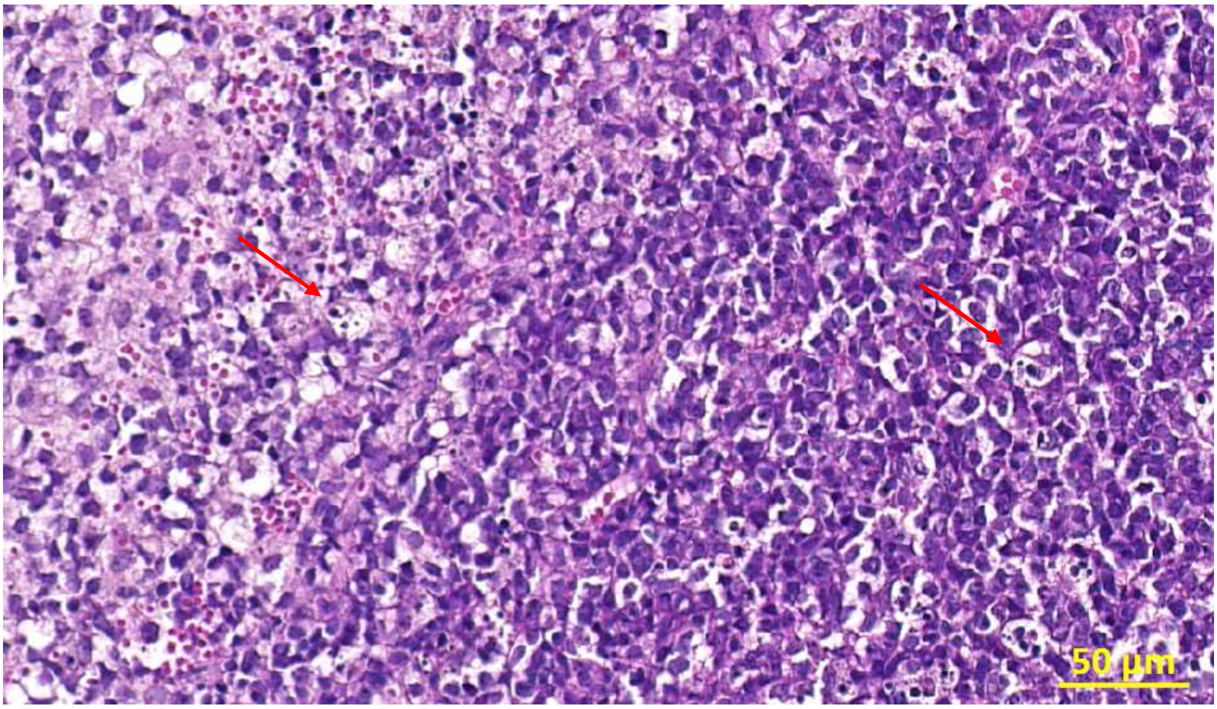

Figure 19: Medium magnification of the daughter malignant lymphoma case 1426-17

This specimen is in the subcutis adipose tissue, with diffuse invasion. The lymphoma cells are small/medium sized, measuring 7-8 µm and there is also as in index case macrophages with phagocytized apoptotic debris ("starry sky" aspect) – see red arrows.

The morphological similarity with the index case (see Figure 18) is striking.

Morphological diagnosis: Malignant lymphoma, small cells, intestine

Source file:

TPL Study Phase Number 780/17: 2019-01-25 12_33_53-#10445tu(da)_34-14_1426-17.czi - ZEN 2.3 lite.png

JA-0021 sMDI: Malignant Lymphoma (ML)

In the case of tumor **JA-0021** (Fig. 2), starting from various suspicious tissues, an enlarged lymph node from female C3H/HeJ mouse was re-transplanted. Although weakly growing in primary recipient SCID/bg mouse **1205-16.**

no *in situ* Foto

Figure 20: Spontaneous neoplasm study (study #10445): normal skin tissue with a mammary teat, female mouse 0021/14

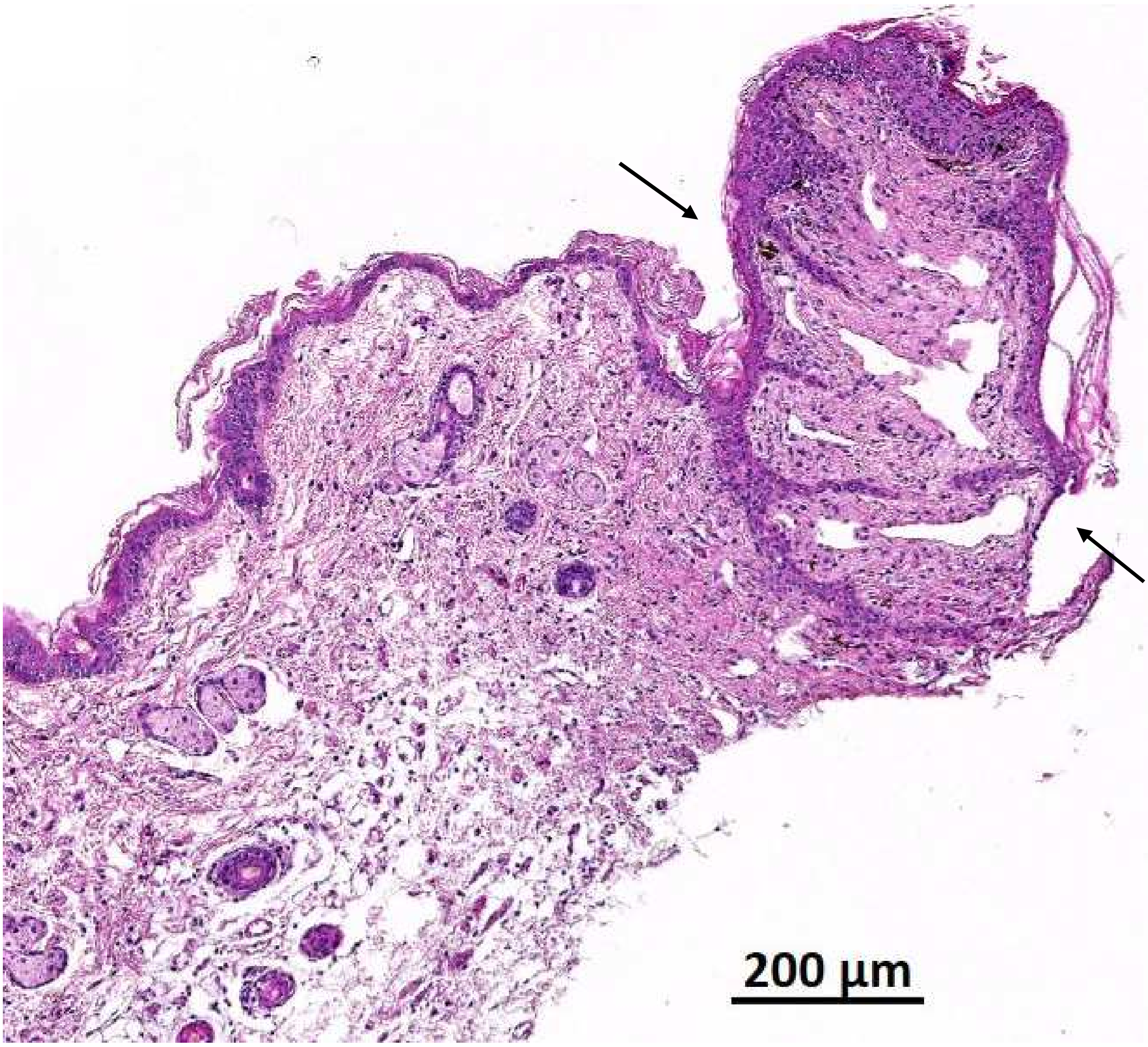

Source: 2017-10-25 15_11_37-TPL811_17_HE_10445_1_mamma_021_14_recut-Rotate-03 - ZEN 2.3 lite

Index case: mouse ID number 0021-14, strain C3H/HeJ, sex: ♀.
These are all normal tissues: there is no evidence of tumors, even after several recuts.
The source neoplasm was not located in this sample.
Morphological diagnosis: Within normal limits (normal skin and teat – see **black arrows**).
Abbreviation: NA (not applicable); ID = identification

Figure 21: Early malignant lymphoma localization in a lymph node – Index 0021-14

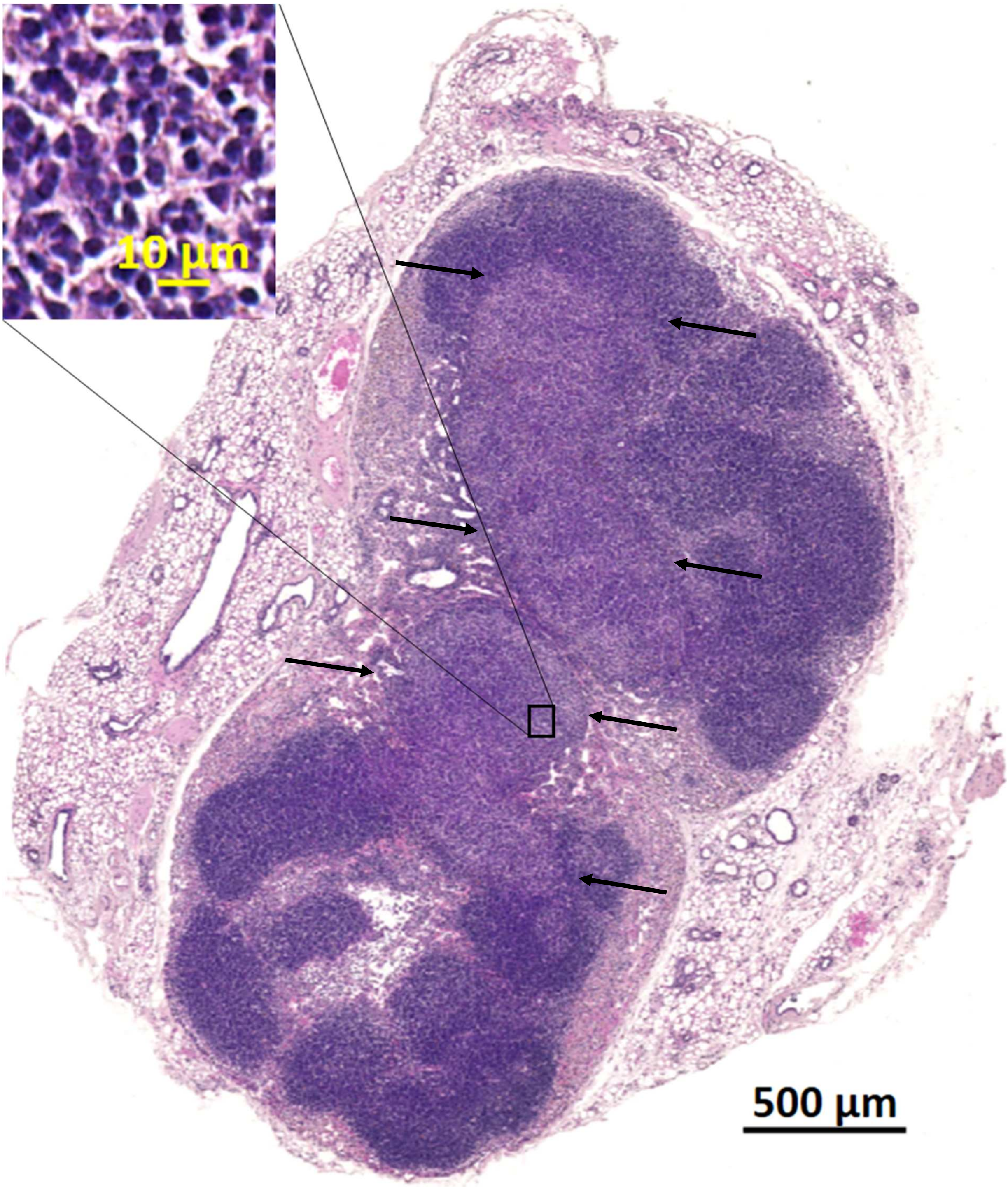

The para-cortical region of this sub-cutaneous lymph node has an early invasion with homogenous sheets of small lymphocytes (see **black arrows** to bracket the foci). Although an atypical lymphocytic immune response cannot be strictly rule out on an isolated case, the context suggests these early sheets of cells to represent malignant lymphoma. The surrounding tissue is mammary gland tissue in adipose tissue of subcutis (panniculus). **Inset:** The lymphocytes are small and homogenous in morphology, measuring 5-7 µm in diameter.

Source file: C:\Users\TLEMARCHAND\Documents\ProQinase-Paper-Project\neue-bilds\2019-01-25 11_09_23-TPL811_HE_10445-1_21-14.czi - ZEN 2.3 lite.png

Morphological diagnosis: Malignant lymphoma, panniculus/sub-cutis, early invasion

Figure 22: Spontaneous neoplasms (study #10445): malignant lymphoma, female SCID *bg/bg* mouse 1571/16

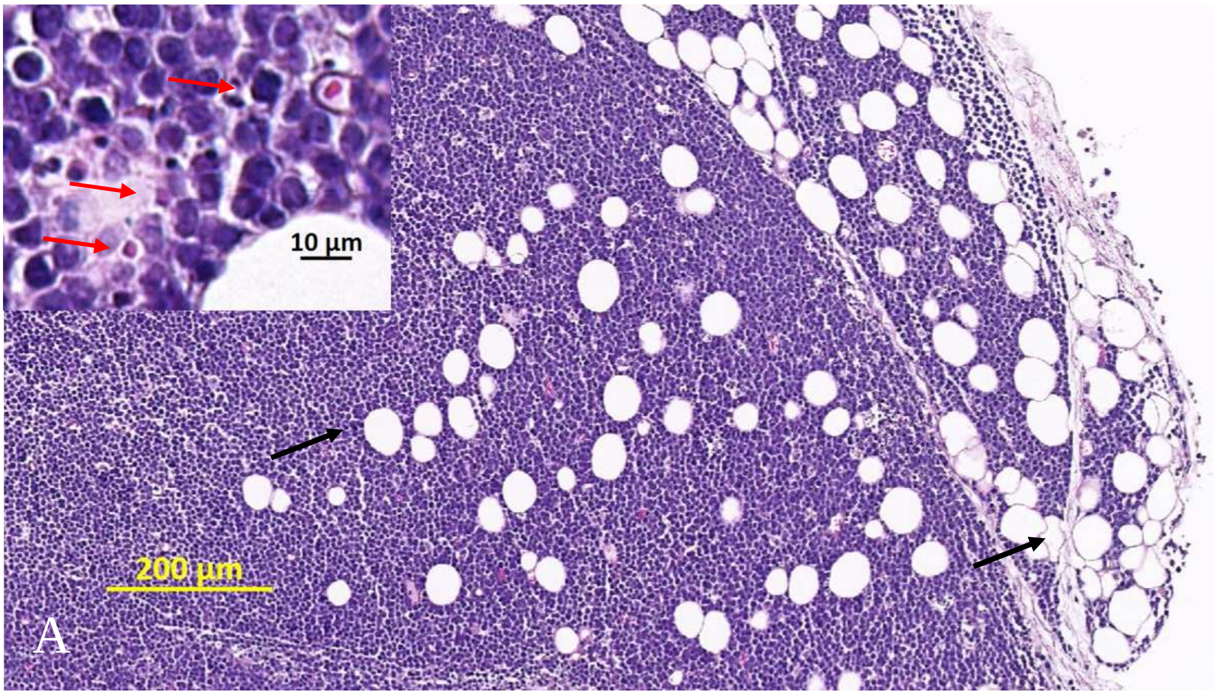

Source: 2017-10-25 14_51_40-TPL811_17_HE_10445_Tu_1571_16.czi - ZEN 2.3 lite

2018-01-22 18_25_10-TPL811_17_HE_10445_Tu_1571_16.czi - ZEN 2.3 lite

Index case: mouse ID number 0021-14, strain C3H/HeJ, sex: ♀.

Figure 22 **A legend:**

There is partial to complete erasing of the normal lymphoid architecture of a lymphoid organ by homogenous sheets of small (5 to 7 µm) round cells (see inset for details), most compatible with blastic lymphocytes, which invade the adjacent tissues, here most probably the adipose tissue of the subcutis and/or the skin (**black arrows** and blue arrow below in **B** showing mammary gland tissue). There is evidence of mitoses and apoptosis (inset of **A**: red arrows). Further characterization of the lymphoma would be an advantage.

Morphological diagnosis: Malignant lymphoma, panniculus/sub-cutis, ♀ SCID *bg/bg*.

Figure 22 **B: Low magnification of tumor aspect overview (thumbnail):**

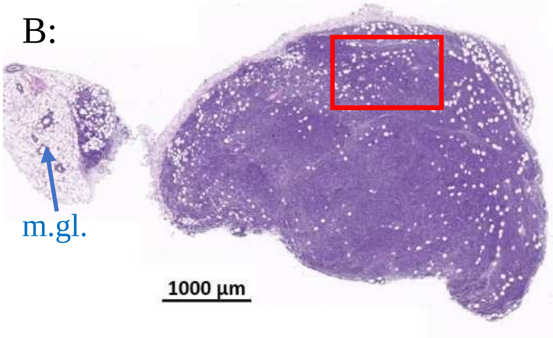

Source photo file (B): 2018-01-22 18_20_25-TPL811_17_HE_10445_Tu_1571_16.czi - ZEN 2.3 lite

The red rectangle indicates the part of the neoplasm, which is depicted above

Blue arrow, m.gl. = mammary gland

Comments on Tumor lineage JA-0011 sMDI:

This case is a borderline one because 2 situations might have arisen:

1. The tumor lineage might have had indeed originated from cells present in this early invasion by small lymphocyte neoplastic cells noted in Figure 21 above

2. this tumor might have spontaneously arisen from the immuno-suppressed beige mouse 1571/16 (Figure 22), and might not be derived from the index case 021-14.

IHC could be useful to further characterize the tumors and confirm lineage.

JA-0009 sMDI: Adenocarcinoma, anaplastic, invasive

Tissue of origin of tumor **JA-0009/ 1286-16**

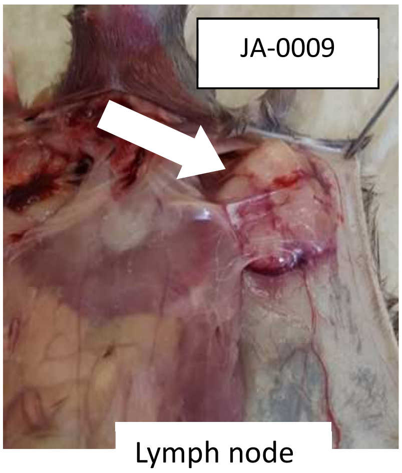

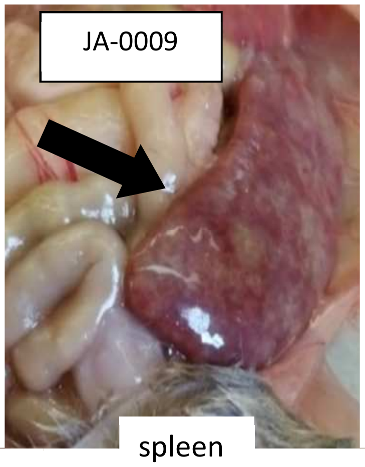

Figure 23: Macroscopic aspects of the index tumor **009-14** (index case for lineage JA-009)

The index animal had a subcutaneous mass in the lymph node region and a markedly enlarged spleen, suggesting a malignant lymphoma (see Figure 23). Histology showed the "lymph node" tumor to be a transmissible neoplasia of epithelial glandular origin (Figure 27 and Figure 24) located in the skin/subcutis with possible anatomical localization in/adjacent to a lymph node or other organ (origin could be apocrine sweat gland, mammary gland etc.). It was an aggressive neoplasm with abundant epidermoid (Figure 25 – actually predominant feature of the daughter transmitted neoplasm 1286/16 see Figure 28 and Figure 29) and spindle cell differentiation (Figure 26). As the spleen was not sampled for microscopic evaluation, the presence of metastases of the epithelial neoplasm (carcinoma) could not be verified, and therefore a concomitant neoplasm of the hemolymphopoietic lineage (leukemia or lymphoma) cannot be strictly ruled out in this particular case.

Histological classification: Skin, subcutis, epithelial neoplasia, glandular (tubular), epidermoid and spindle shape cell differentiation

Diagnosis: Adenocarcinoma, solid, invasive, NOS

Main features: Solid (trabecular), invasive, anaplastic

Mitotic index: 3,25 mitoses per high power fields (phpf) for index case 009-14 and 3.125 mitoses phpf for the derived tumor 1286/16.

Necrosis: Yes (coagulative, disintegrative, with neutrophils), SCN (pyknosis, karyorrhexis), compatible with presence of apoptosis - Overall necrosis grade : minimal

Main differential diagnosis: epidermoid carcinoma, carcinosarcoma (malignant glandular mixed cell tumour), anaplastic carcinoma

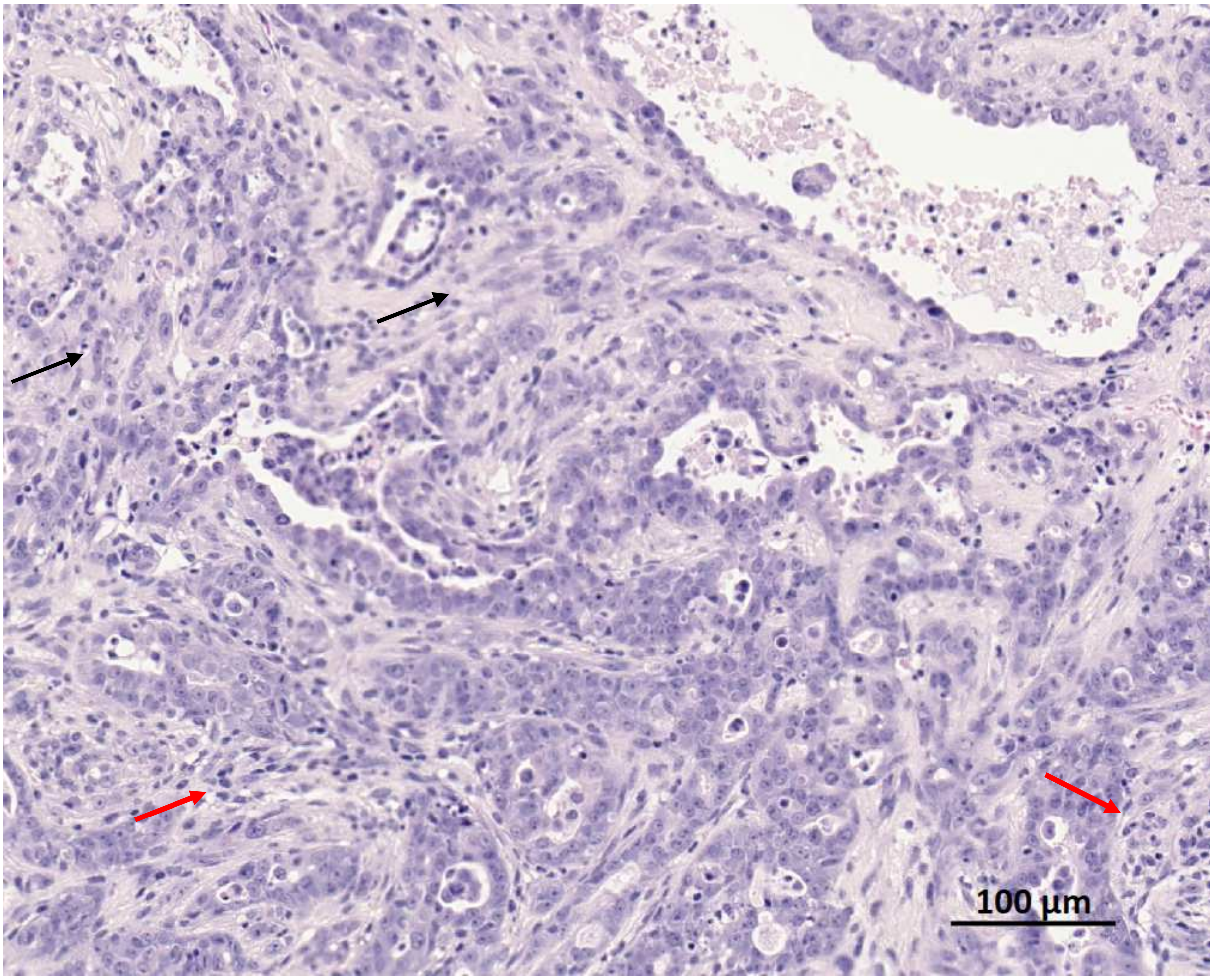

Figure 24: Medium high magnification of the tumor index case 009-14, with glandular and anaplastic/ spindle shaped differentiation

Medium high magnification of the tumor index 009-14, showing evidence of glandular differentiation, with areas of anaplastic/spindle shaped differentiation (**black arrows**). Host inflammatory cells are moderately numerous (red arrows).

Source file: 2019-01-23 16_25_06-TPL_601_16_009_14_Tu.czi - ZEN 2.3 lite.png

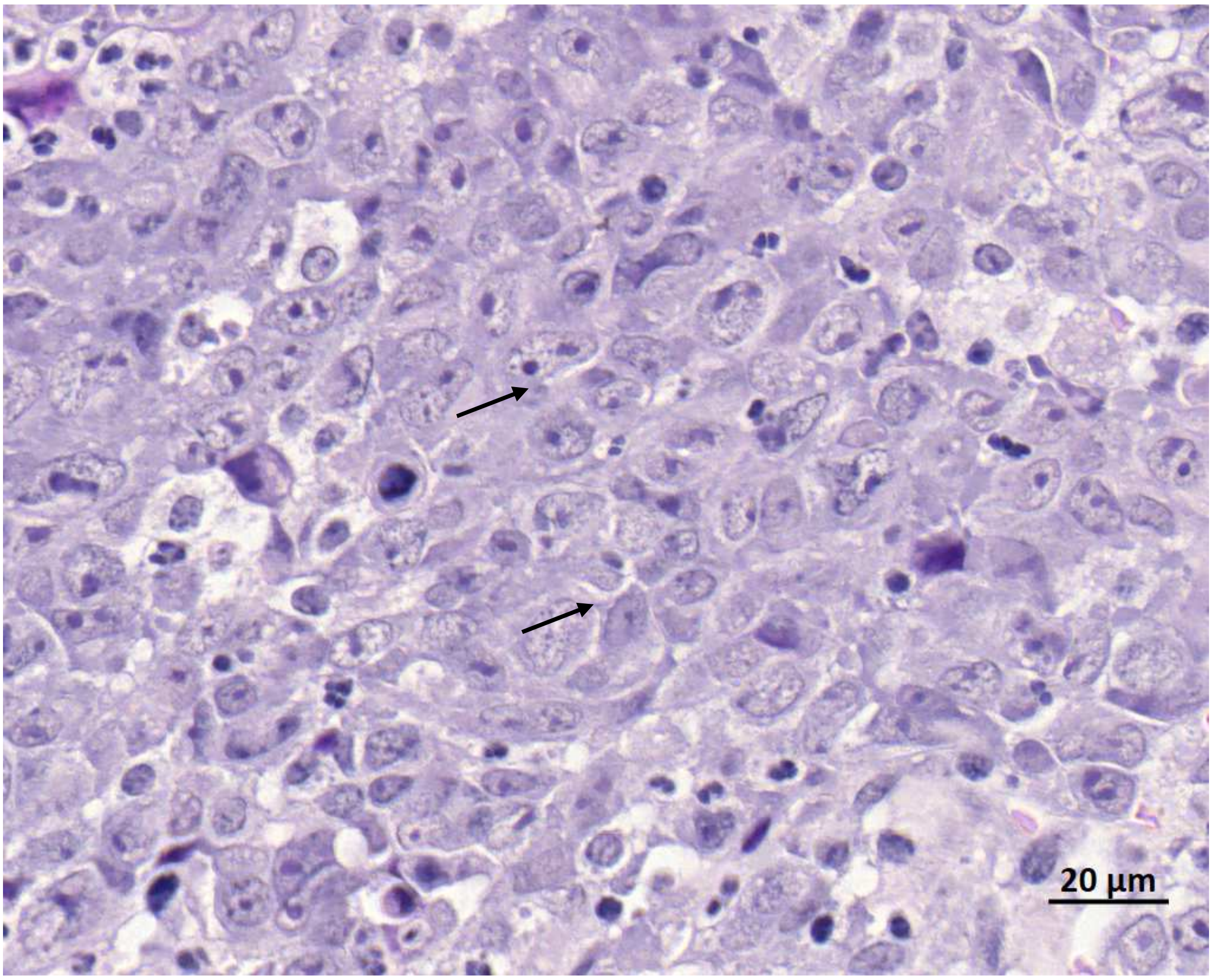

Figure 25: High magnification of the tumor index case 009-14, with clear evidence of epidermoid differentiation, suggesting an epidermoid carcinoma

The intercellular bridges are diffusely very distinct in this thin section of the tumor (**black arrows**).

Source file: 2019-01-24 21_34_29-TPL_601_16_009_14_Tu.czi - ZEN 2.3 lite.png

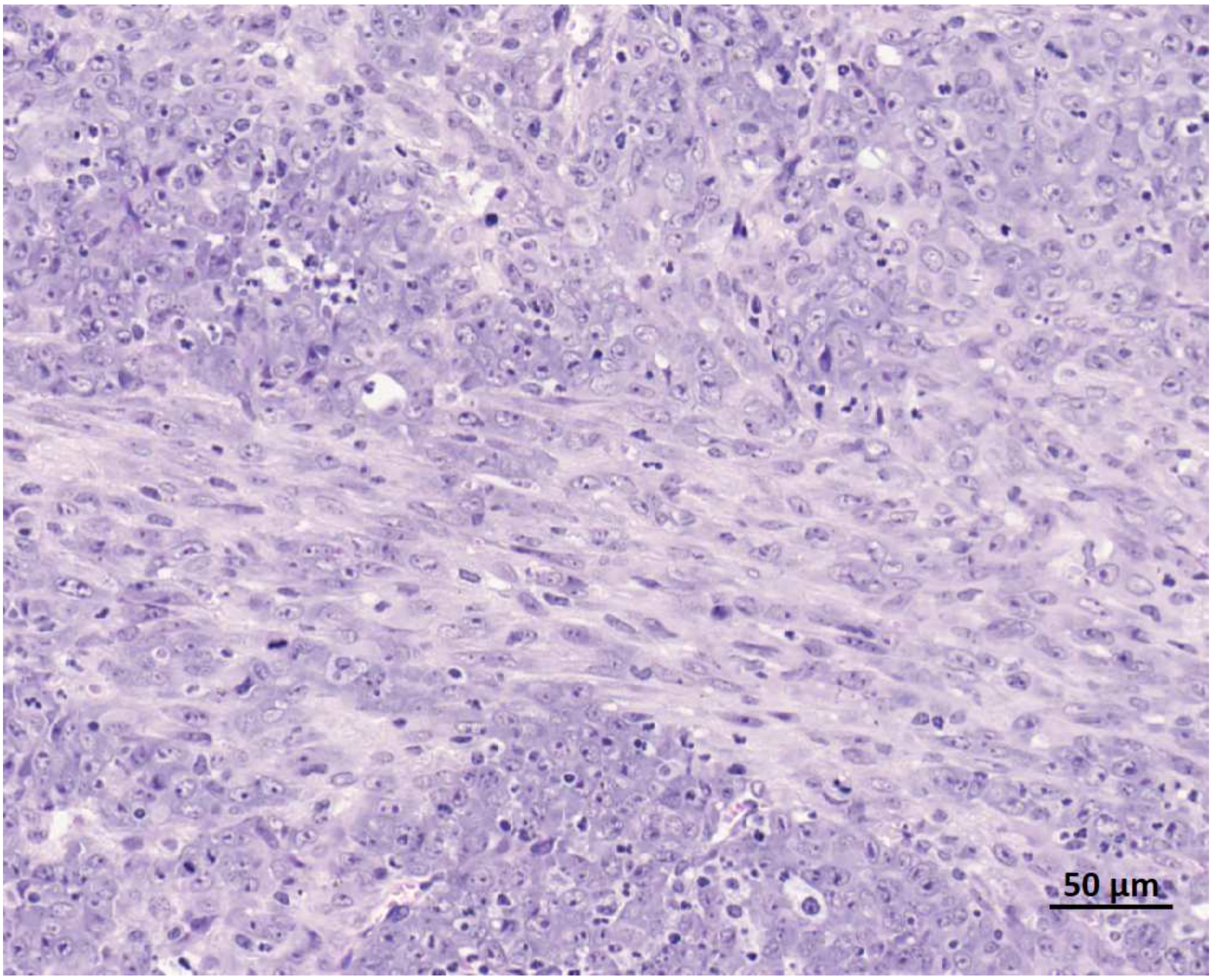

Figure 26: A moderate high magnification view of 009-14 showing spindle shaped cell differentiation

The epithelial to mesenchymal transition and the epidermoid differentiation, with a clear anaplastic (isolated neoplastic cells) and spindle-shaped neoplastic cells (suggesting a malignant mixed cell tumor of glandular origin or carcinosarcoma) suggest the neoplasm to be of salivary or apocrine sweat gland or also possibly of mammary gland origin.

Source file: 2019-01-24 22_11_08-TPL_601_16_009_14_Tu.czi - ZEN 2.3 lite.png

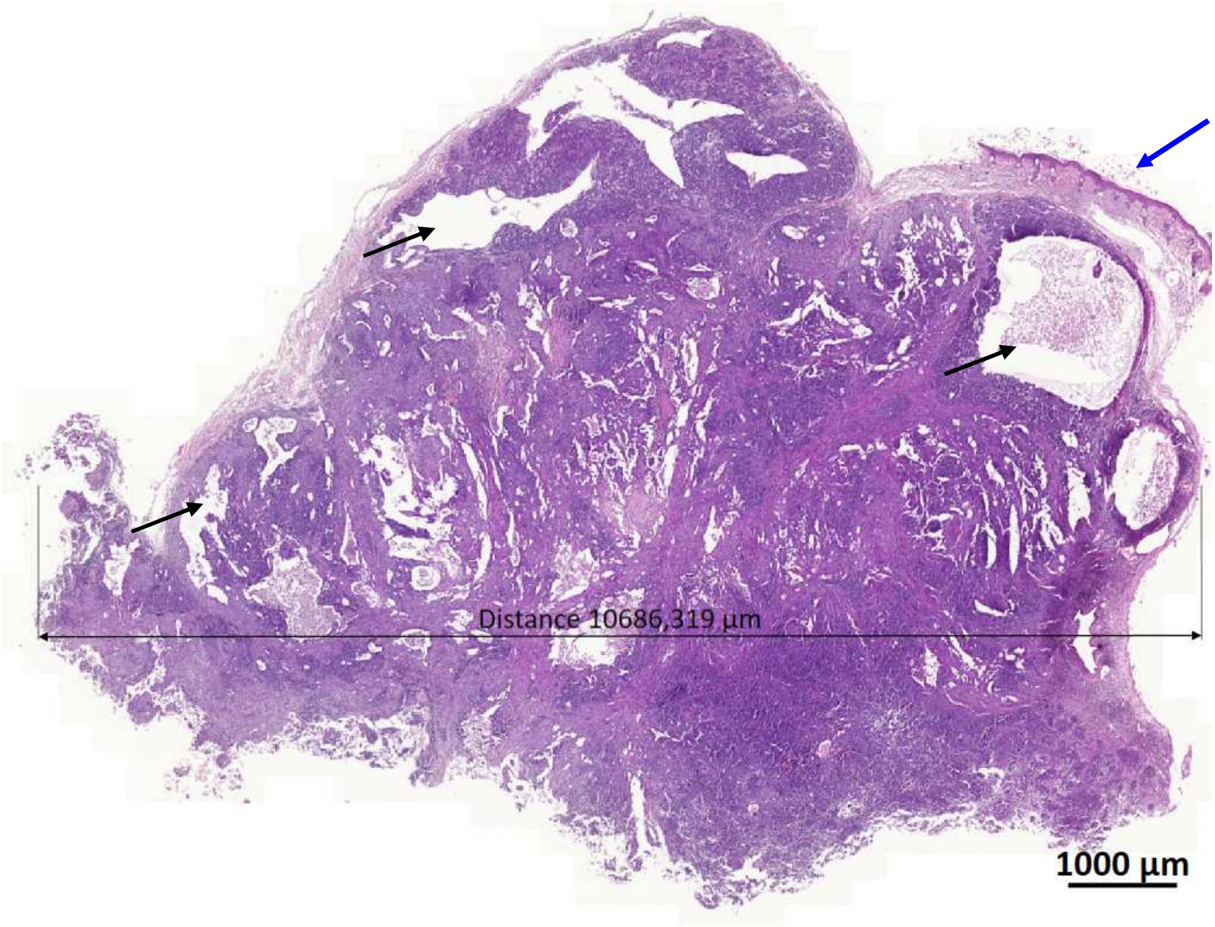

Figure 27: Low magnification view of the index neoplasia 009-14

There is presence of cysts with papillary proliferations and many tubular profiles, with abundant evidence of secretion materials and necrotic debris, demonstrating the glandular origin of this aggressive neoplasm (**black arrows**). The tumor is clearly cutaneous/sub-cutaneous (blue arrow).

Source file: 2019-01-24 22_20_14-009_14.czi - ZEN 2.3 lite.png

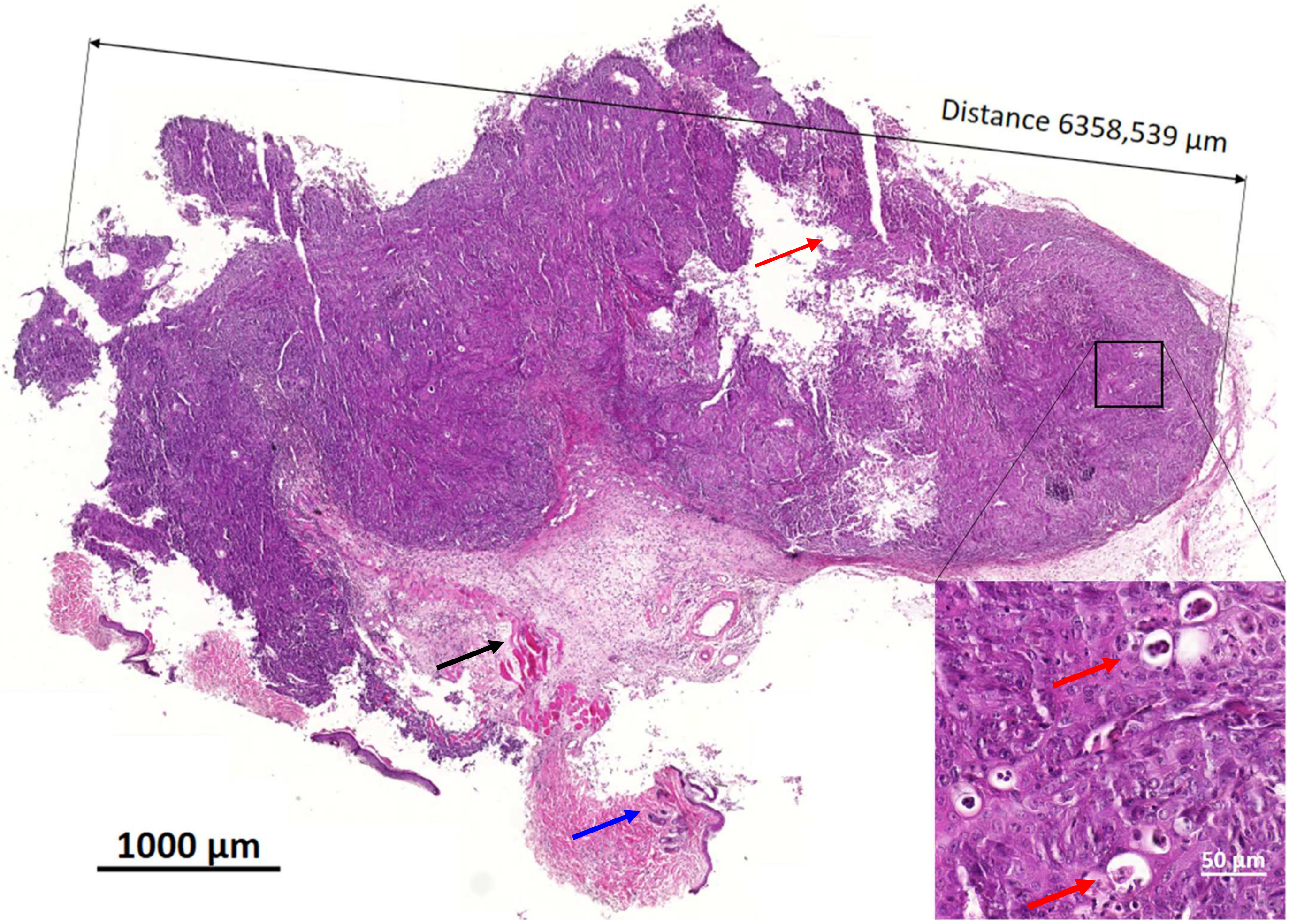

Figure 28: Low magnification aspect of the transmitted tumor 1286/16 from index case 09-14

Low magnification aspect of the transmitted tumor 1286/16: essentially solid aspect with still limited evidence of mucinous and/or glandular differentiation (inset: **red arrows**). The solid epidermoid carcinoma component is now predominant, almost exclusive, with more eosinophilia associated with keratinization. The tumor is also developed in the subcutis adjacent to cutaneous muscle (**black arrow**) and right under the skin (blue arrow).

Source files:

2019-01-24 23_10_30-1286_16.czi - ZEN 2.3 lite.png

2019-01-24 22_57_44-1286_16.czi - ZEN 2.3 lite.png

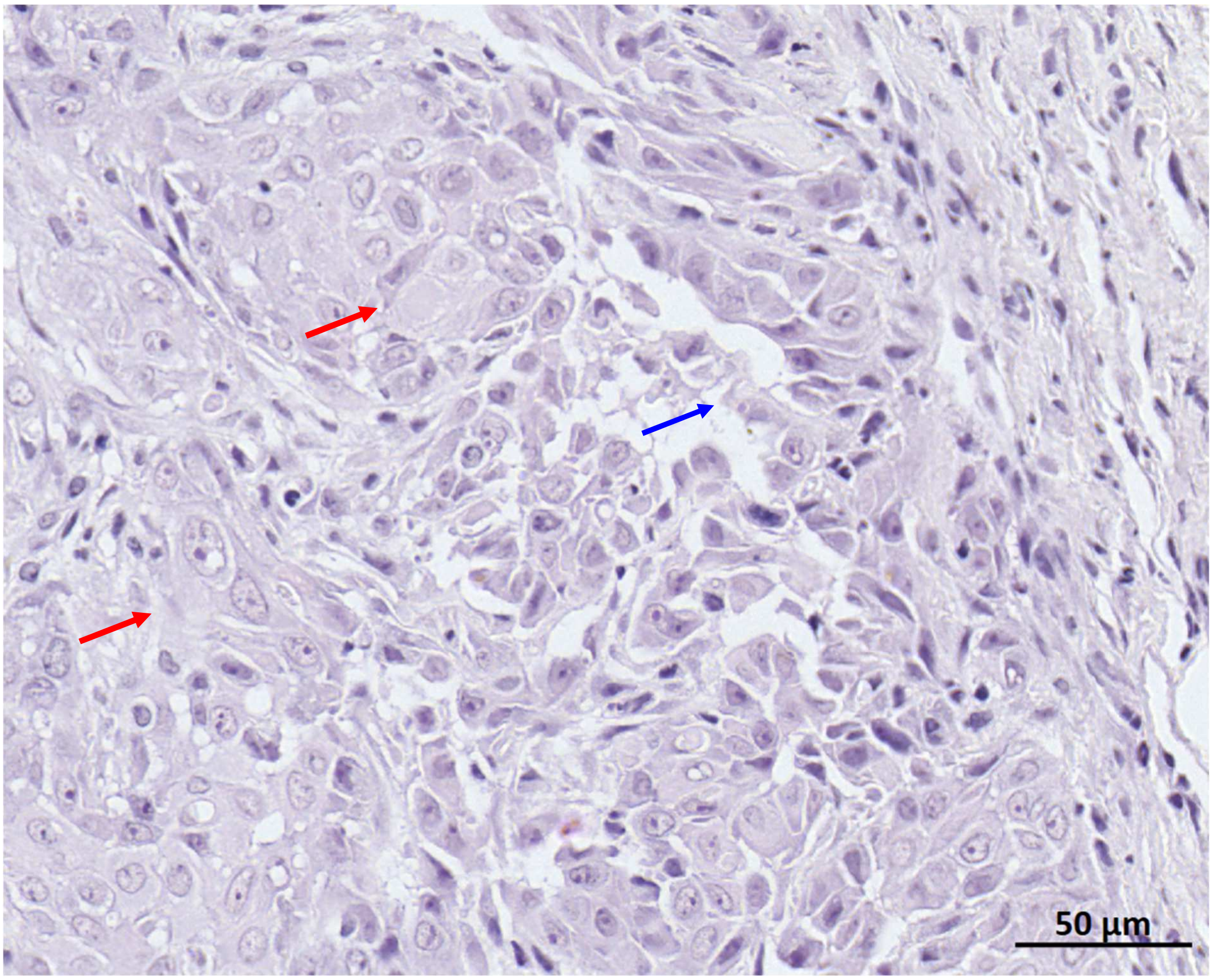

Figure 29: High magnification aspect of the transmitted tumor 1286/16

High magnification aspect of the 1286/16 with epidermoid differentiation and abundant pale eosinophilic cytoplasm (red arrows) and acantholysis with pseudo-glandular formation (**blue arrow**). The epidermoid differentiation is in this case the main component and therefore a differential diagnosis is **epidermoid carcinoma**

Source file: 2019-01-24 22_08_31-TPL_601_16_009_14_Tu_1286.czi - ZEN 2.3 lite.png

JA-0017 sMDI: Adenocarcinoma, complex type

Index case **JA-0017/ 0096-17** seemed at first analysis to exhibit some significant degree of phenotypic drifting. Tumors from the two daughter mice (1234-16 and 0096-17)

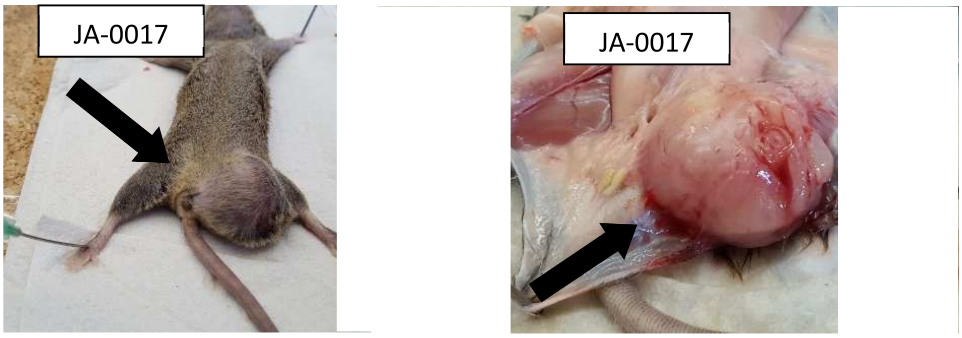

Figure 30 : Gross aspects of the index neoplasm 0017-14

Adenocarcinoma, complex type, or "mixed cell tumor",

The proliferation is composed of both myoepithelial and glandular elements with preserved ductular elements. These elements are well differentiated and benign looking.

This is an epithelial proliferation from an exocrine gland, of undetermined origin. The complex nature (see below) suggests it to be most likely of apocrine sweat gland or mammary gland origin.

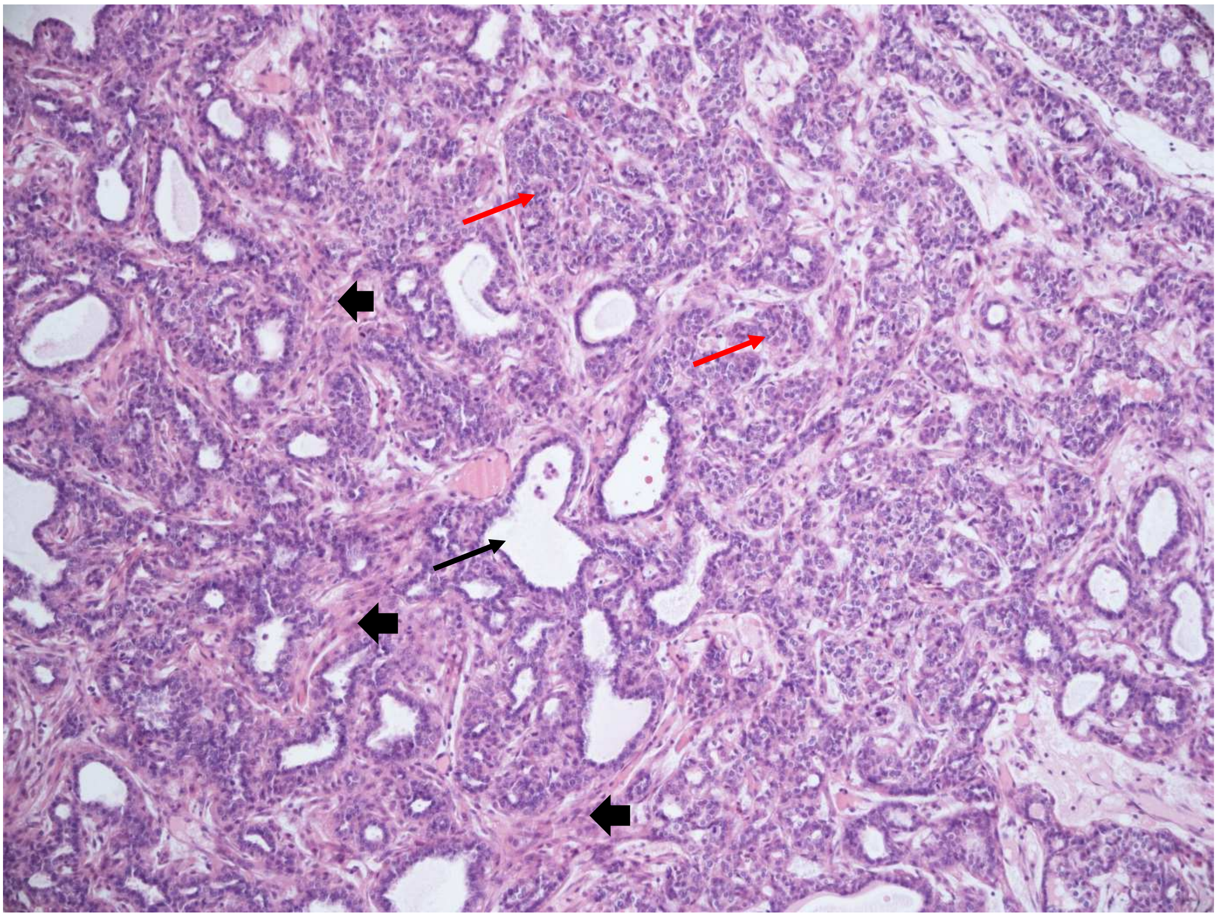

Figure 31: Complex adenocarcinoma (mixed tumor) -index-case, HE stain, original magnification: 100x

Index case: mouse ID number 017-14. Strain CBA/J ♀

This is an epithelial proliferation from an exocrine gland, of undetermined origin. The complex nature (see below) suggests it to be most likely of apocrine sweat gland or mammary gland origin.

The proliferation is composed of both myoepithelial (**arrowheads** ⬅) and glandular elements (**red arrows**) with preserved ductular elements (**black arrow**). These elements are well differentiated and benign looking. This neoplasm was however transmitted.

Morphological diagnosis: Complex adenocarcinoma/Mixed Tumor/Carcinosarcoma.

Abbreviation: c-ADK/MT/KS

Image ID: 780_#10445_tu01_0017-14_0017-14_HE_10x_1-AdK--mixed tumor-index-case, HE stain, original magnification: 100x

Figure 32: Complex adenocarcinoma (mixed tumor) – myoepithelial aspect, derived case mouse 1234-16, HE stain, original magnification: 200x

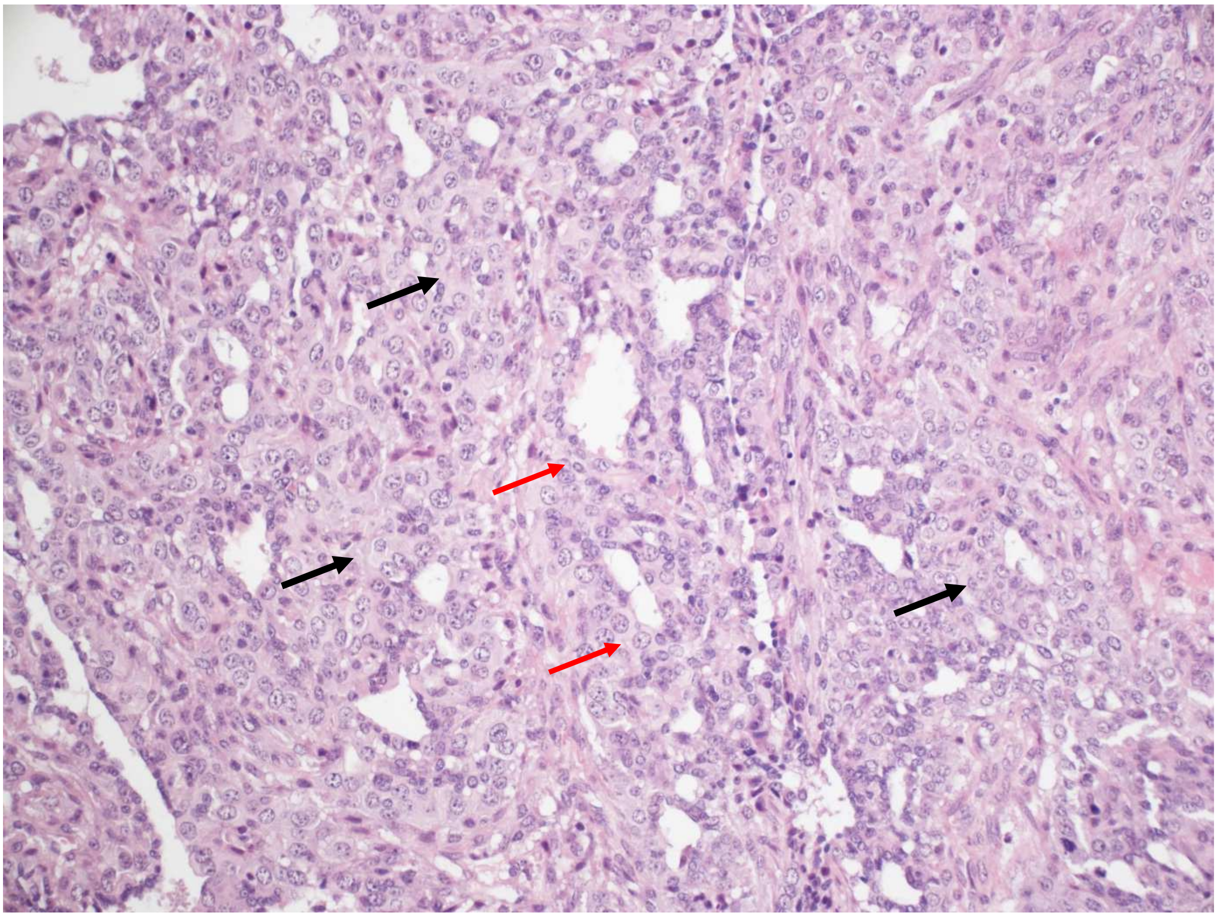

Mouse ID #1234-16; from index case: mouse ID number 017-14. Strain CBA/J ♀.

The proliferative component of this tumor is mostly myoepithelial (**black arrows**) with well differentiated glandular elements (**red arrows**).

Morphological diagnosis: Complex adenocarcinoma/Mixed Tumor/Carcinosarcoma.

Abbreviation: c-ADK/MT/KS

Image ID: 780_#10445_tu_0017-14_1234-16_HE_20x_2-AdK-wKeratinization-mixed tumor-complex-myoepiith-epiith2lmesenchT-wC,

HE stain, original magnification: 200x

Figure 33: Complex adenocarcinoma (mixed tumor) – trabecular clear cell aspect, derived case mouse 0096-17, HE stain, original magnification: 200x

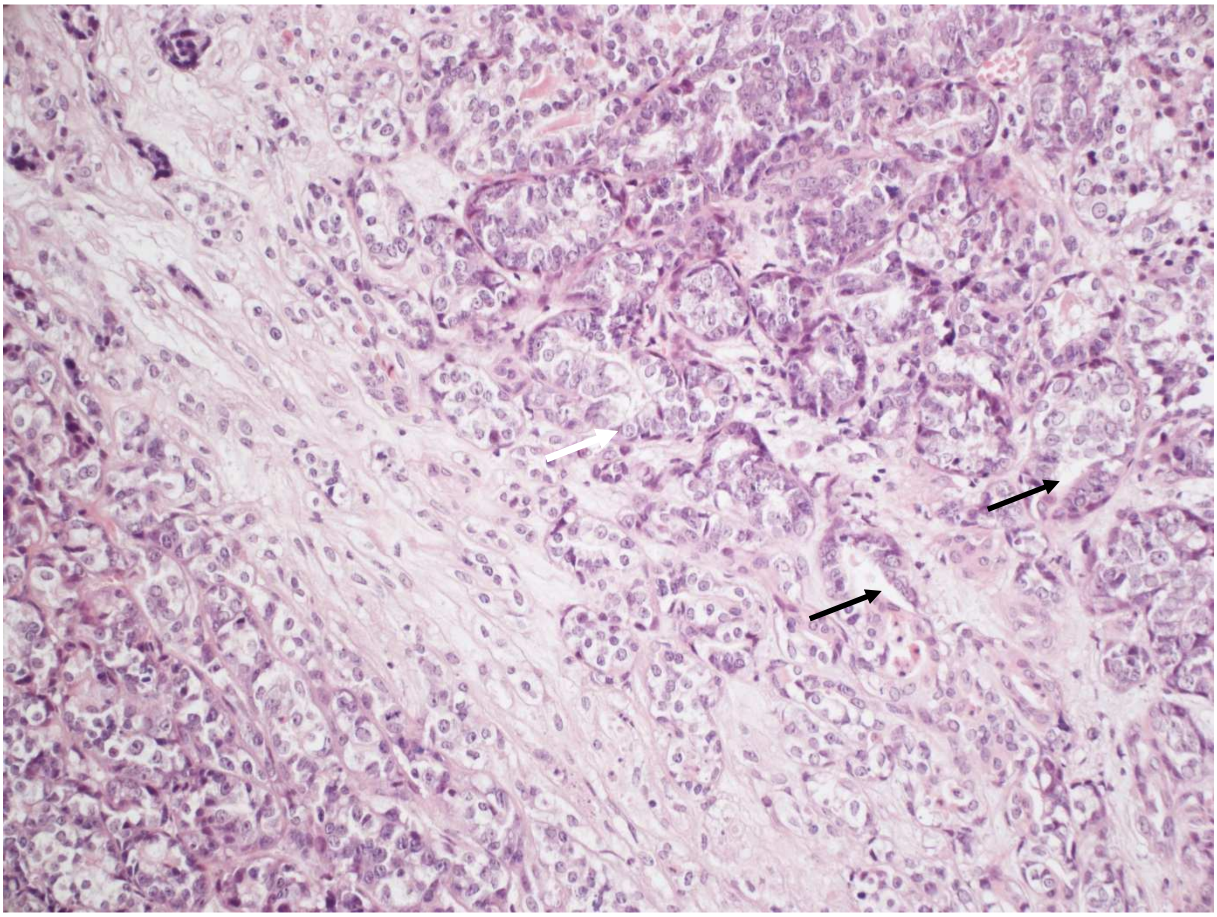

Mouse ID #0096-17; from index case: mouse ID number 017-14. Strain CBA/J ♀.

Trabecular carcinoma composed of eptithelial cells with clear cytoplasm, with occasional luminal differentiation (**black arrows**).

Morphological diagnosis: Trabecular carcinoma, possibly from the index complex adenocarcinoma/Mixed Tumor/Carcinosarcoma.

Abbreviation: c-ADK/MT/KS

Image ID: 780_#10445_tu01_0096-17_0017-14_HE_20x_2-AdK--mixed tumor- trab-K, HE stain, original magnification: 200x

JA-0023 sMDI: Adenocarcinoma, mammary gland

JA-0023 (Fig. 3) were obtained by growing spontaneous tumors either from a primary invasive ADK of mammary gland origin (back tumor – see Figure 36) or a clear cell carcinoma (CCC)-like tumors from the kidney of the single index mouse 0023-14 (Figure 34 and Figure 35). Morphologically, all mouse derived (sMDI) tumors, including a derived (daughter) tumor noted in the kidney of another mouse, e.g. **0001-17** (Figure 39), had morphological features identical or the closest to the mammary index tumor of the back JA-0023 in 0023-14. These 2 index neoplasms and all daughter sMDI, e.g. **0001-17, 0005-17, 1620-16, 0544-17 and 0547-17** (see below) are presented below.

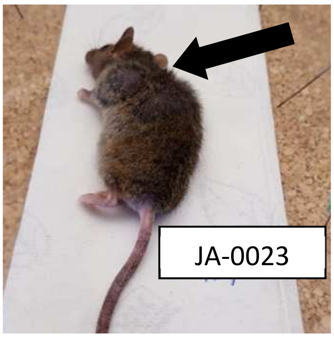

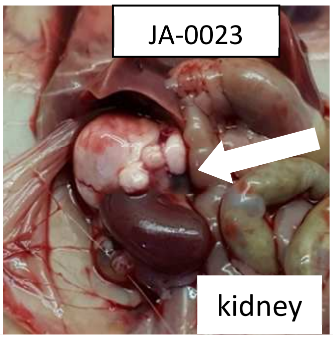

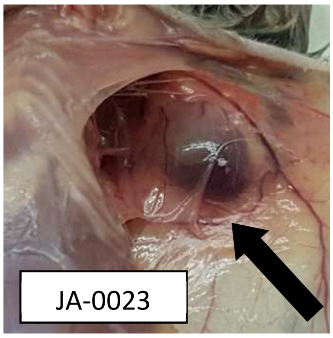

Renal tumor: index case: Carcinoma/adenocarcinoma with clear cell differentiation /Clear Cell Renal Carcinoma – see Figure 34 and Figure 35.

Mammary gland: Invasive ductal cystic-papillary adenocarcinoma with desmoplasia and lymphatic emboli – see index Figure 36 and lineate Figure 37 to Figure 42.

- peripheral perineoplastic lymphocytic infiltration, moderate
- hemorrhage and erythrophagocytosis by macrophages (not shown in photos below)

Given the aggressiveness of the primary tumor (i.e. neoplastic emboles) it is very likely the tumor origin of the tumor lineage s-MDI JA-.0023 is the mammary gland, and not the kidney.

The papillary cystic tumor of mammary gland origin has excellent phenotypic stability (see Figure 37 to Figure 42).

Figure 34: Low (left) and mid (right) magnification views of the index tumor from the kidney

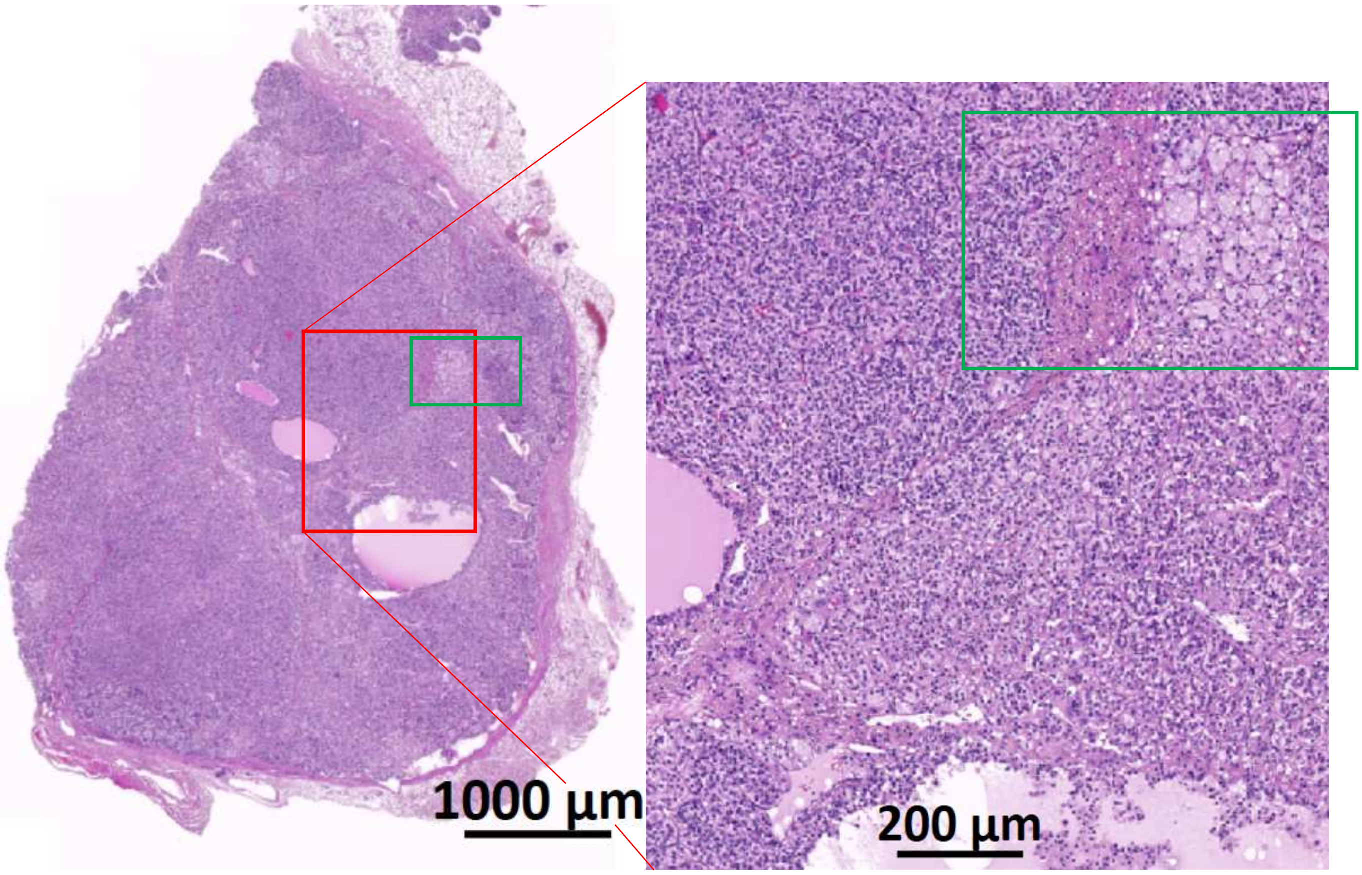

Figure 35: High magnification view of the index renal tumor

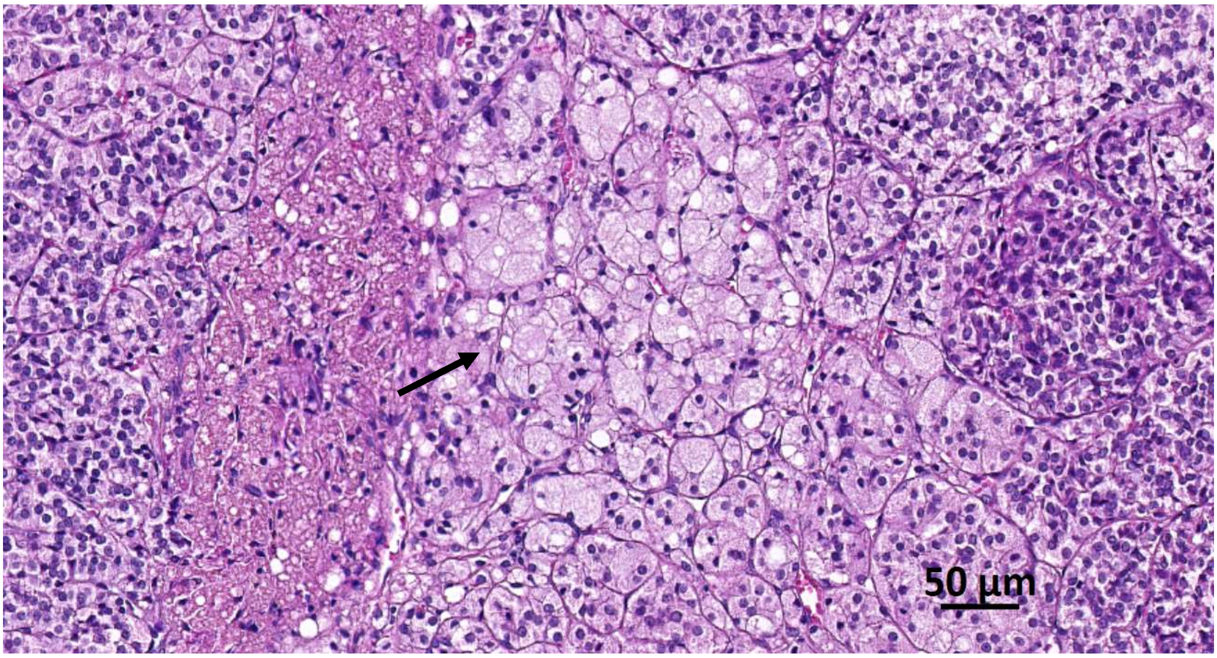

Green square inset: High magnification view of the clear cell differentiation in the index tumor (**black arrow**), which is focally reminiscent of a clear cell renal carcinoma, the most common renal neoplasm in humans. **Source file:** *2019-02-03 21_43_30*-10445_tu_nie_0023-14_0023-14.czi - ZEN 2.3 lite.png

Figure 36: Index tumor of the subcutaneous tumor: microscopic morphological aspects

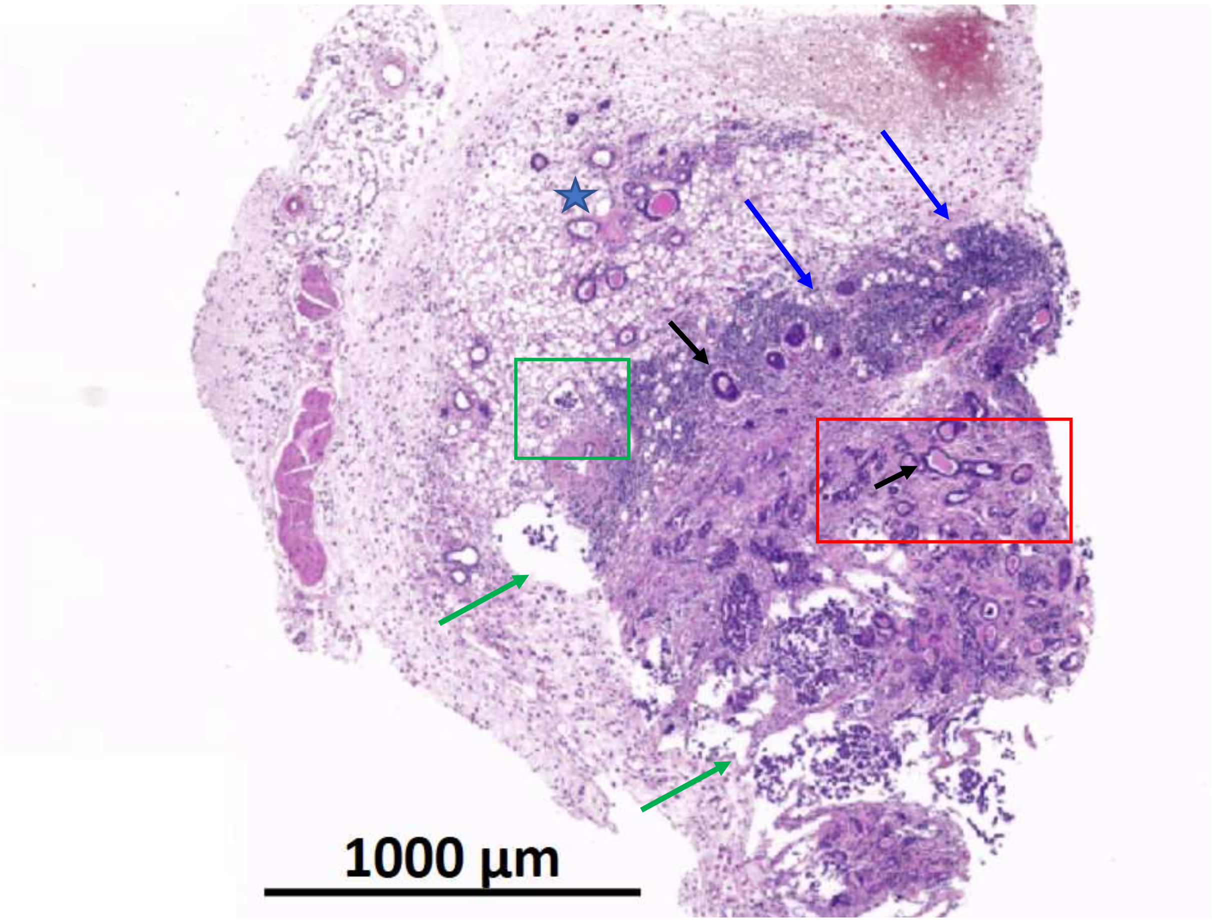

Low magnification aspect of the subcutaneous neoplasm: there is a mass developed from the mammary gland (★) and in the adipose tissue, of malignant glandular ductular profiles with varying glandular secretion (**black arrows**), invading into the adjacent subcutis and eliciting an abundant fibroplasia (desmoplastic change) and abundant lymphoid response (blue arrow) and dilated lymphatics and blood vessels (green arrows and green square).

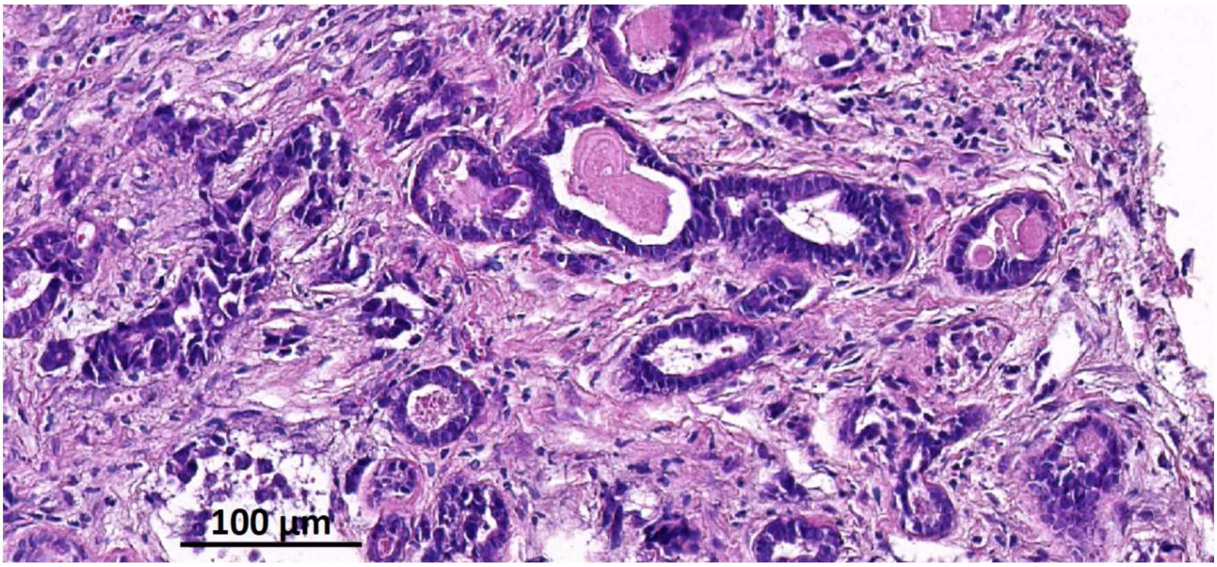

Red square: The tumor consists of ductular profiles developed in a dense fibroblastic tissue.

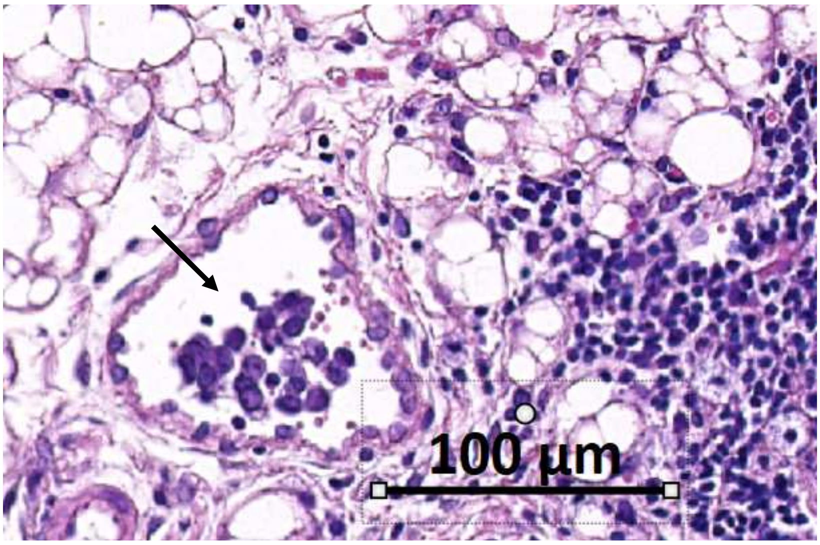

Green square: an example of individualized neoplastic cells in a vein (see **black arrow)** with a few red blood cells. On the right of the photo there is numerous small mononuclear cells, most compatible with small lymphocytes.

Figure 37: Lineage tumor aspect in subcutaneous tissue: example of **0005-17**

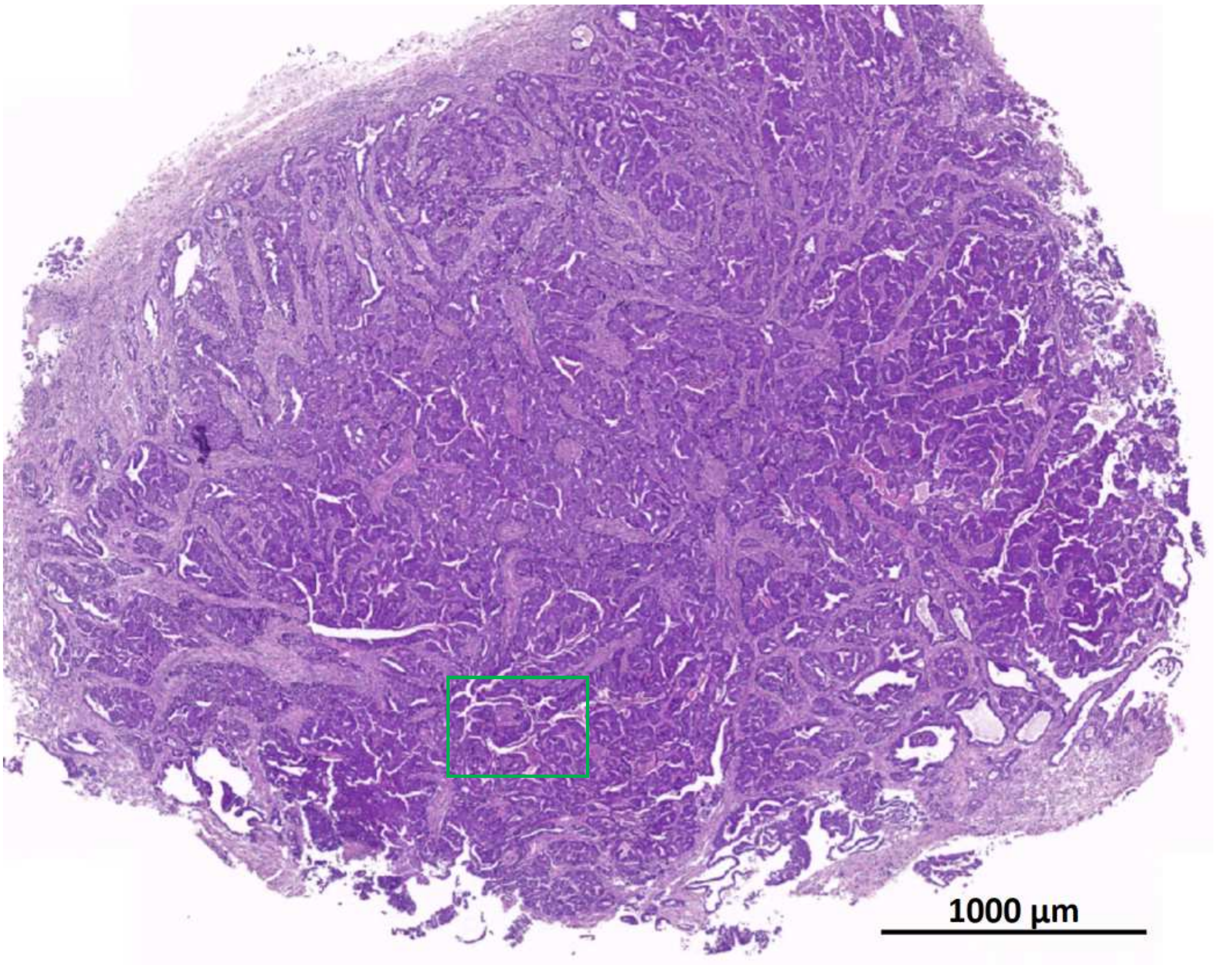

The neoplasm 005-17 is similar to neoplasm 0023-14 from the subcutis (see Figure 36), with a somewhat more papillo-tubular than tubular and less desmoplastic morphology **Source file**: 2019-02-04 15_42_24-10445_tu_rue___0023-14___0005-17.czi - ZEN 2.3 lite.png

Figure 38: Case 0005-17: High magnification reveals the tubulopapillary structure with necrotic cells in lumens and supportive stroma

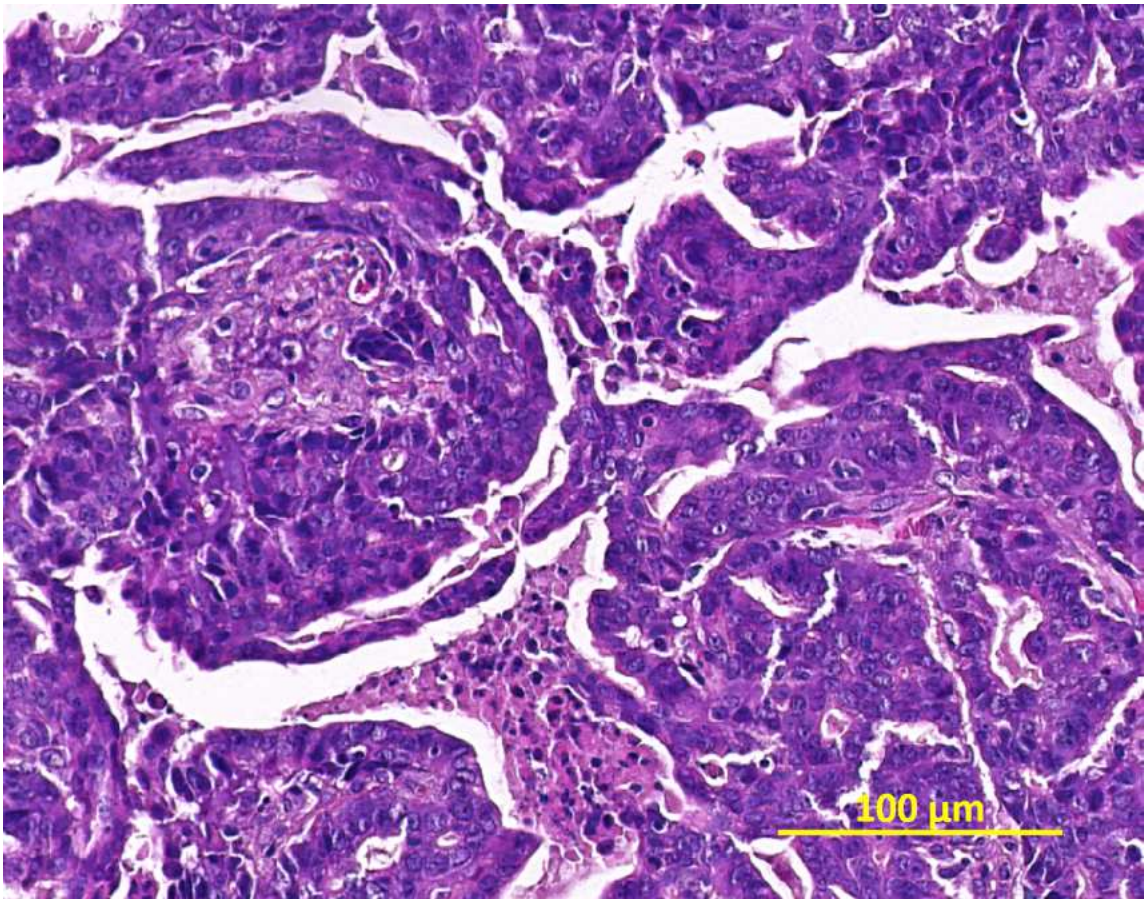

High magnification is more cellular than the index tumor from the subcutis (see Figure 36).

Figure 39: Lineage tumor aspect in kidney: example of **0001-17**

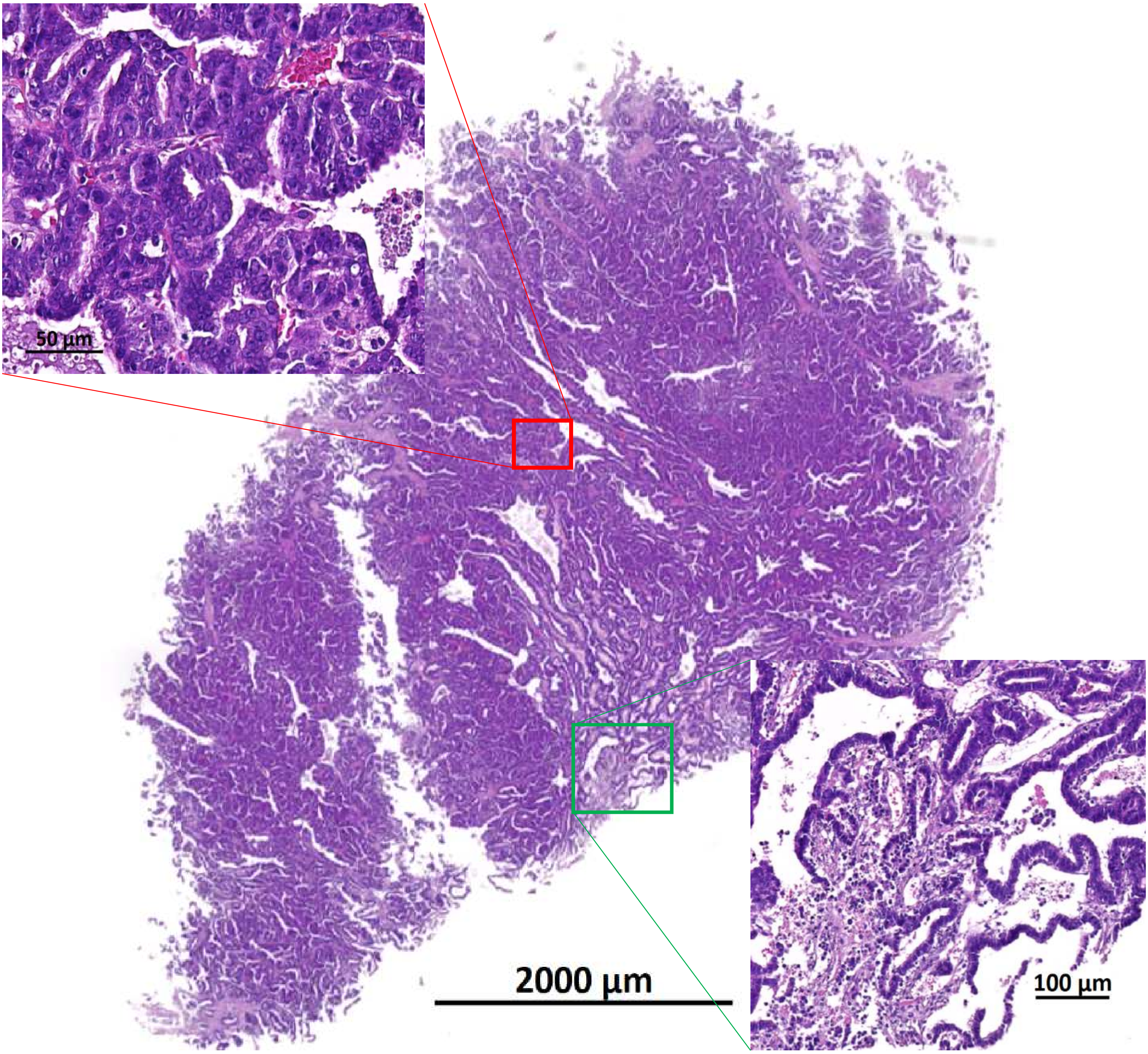

Notice the tumor aspect is very different from the renal tumor from the index case 0023-14 (see Figure 35) but similar morphologically to all other tumors from the lineage (see Figure 38, Figure 40, Figure 41 and Figure 42), including the subcutaneous index mammary cancer (Figure 36). High magnification is identical to the lineage tumor 0005-17 (Figure 38). There is no evidence of normal renal tissue on the section examined. The tubulopapillary pattern is present, as well as the host immune response (see lower inset of the green square)

Figure 40: Lineage tumor aspect in subcutaneous tissue: example of **1620-16**

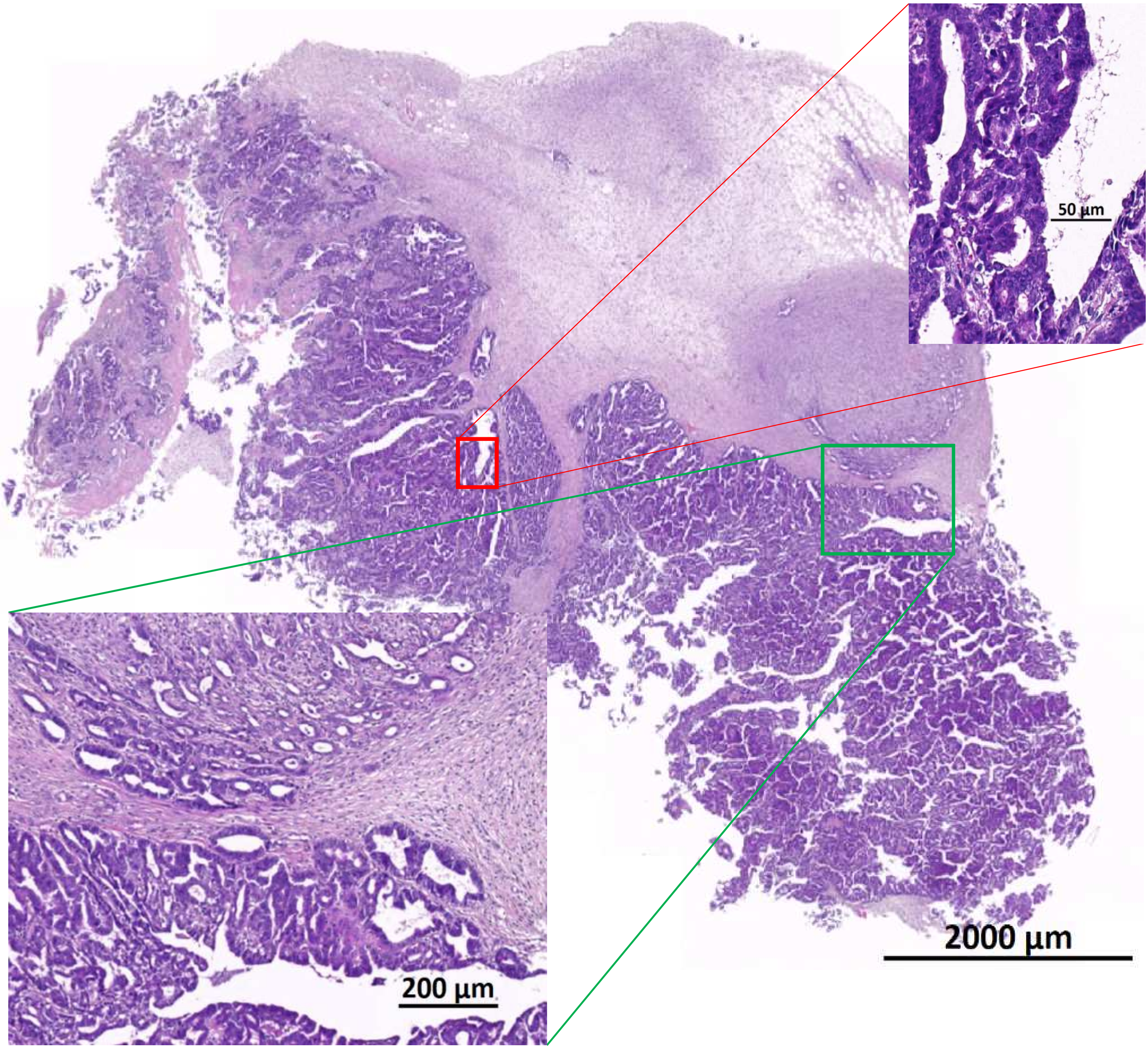

This daughter subcutaneous tumor is like all the tumors of the lineage, with however regions with more distinct desmoplasia and abundant lymphoid infiltration (see green square inset). Cytological aspect of the tubulopapillary proliferations are very similar in all the tumors from this lineage, indicating phenotypic stability.

Figure 41: Spontaneous neoplasms (study #10445): papillary cystic adenocarcinoma, female mouse 0547/17, hematoxylin and eosin stain, subcutis

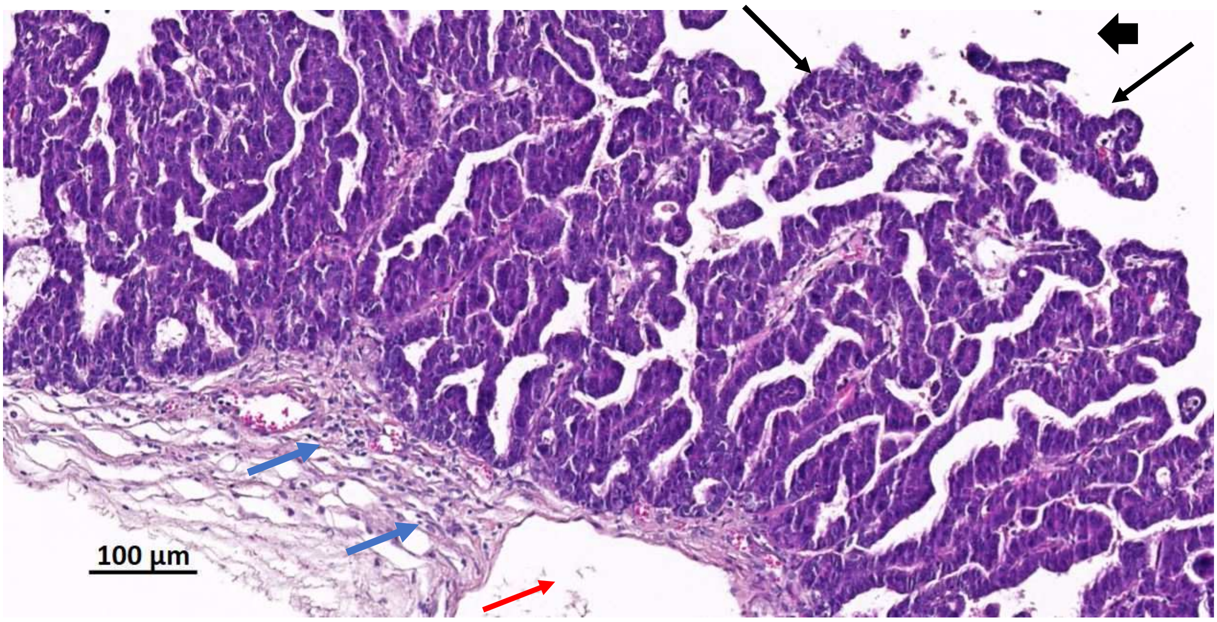

Image ID: 2017-10-25 15_23_04-TPL811_17_HE_10445_Tu_0547_17.czi - ZEN 2.3 lite

Index case: mouse ID number 023-14, strain C3H/HeJ ♀. Female C3H/HeJ 0547/17

The index case was previously studied in phase study TPL Path Labs TPL780-17.

The malignant proliferation is characterized by epithelial cell proliferation forming tubules and papillar (glandular) differentiation (**black arrows**), developed in a 6.8-millimeter long, multi-locular cystic cavity (arrowhead ⬅). Accompanying this epithelial malignant tumor of glandular origin are slight to moderate, stromal mononuclear leukocyte infiltrates, associating both small lymphocytes (**blue arrows**) and some macrophages. A lymphatic vessel is markedly dilated (**red arrow**).

Morphological diagnosis: Adenocarcinoma (Adk), papillary-cystic, mammary gland.

Low magnification of tumor aspect overview (thumbnail):

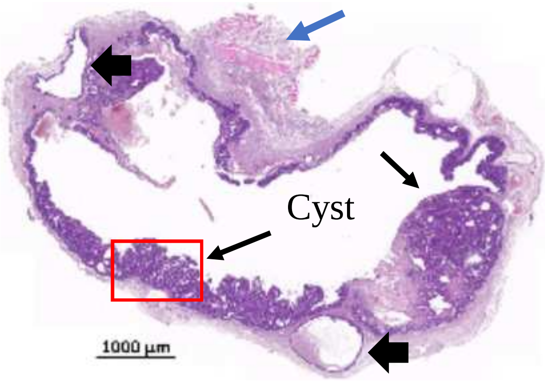

The red rectangle indicates the part of the cyst wall, which is depicted above at higher magnification.

Source photo file: 2017-10-25 14_55_01-TPL811_17_HE_10445_Tu_0547_17.czi - ZEN 2.3 lite

Low magnification view of tumor case ID # 0547. The neoplasia is developed in 7x4-mm cyst, with a multi-locular pattern (development of secondary cysts – see arrowheads ⬅).

Figure 42: Spontaneous neoplasms (study #10445): papillary cystic adenocarcinoma, female mouse 0544/17, skin

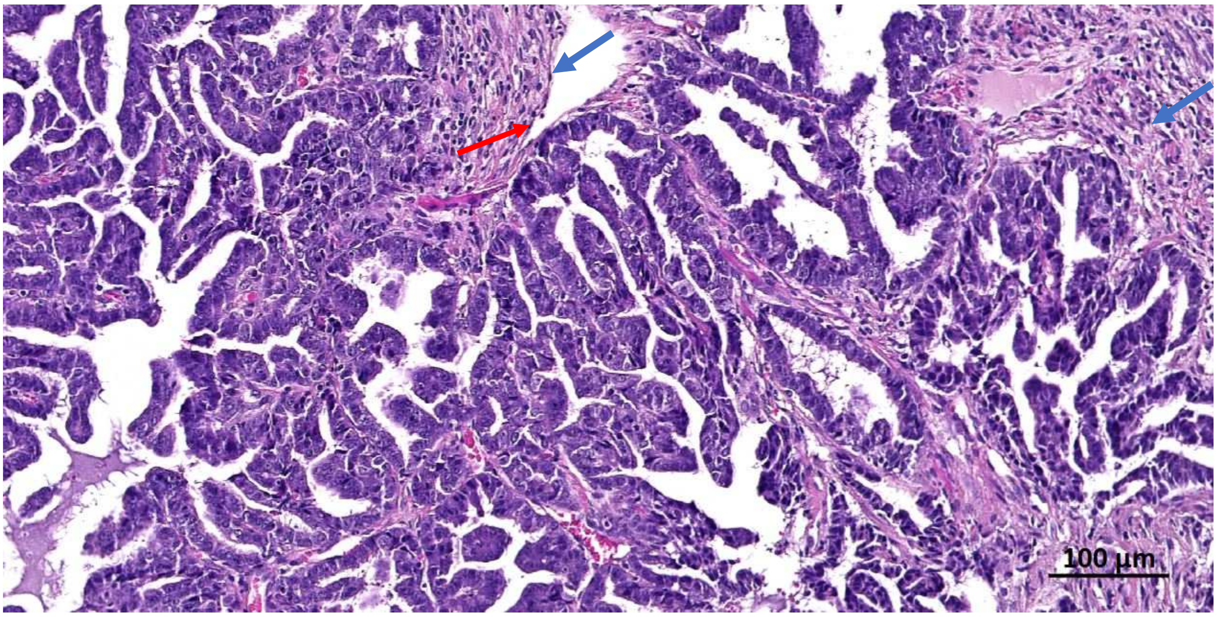

Image ID: 2017-10-25 15_20_49-TPL811_17_HE_10445_Tu_0544_17-Rotate-01 - ZEN 2.3 lite

Index case: mouse ID number 023-14, strain C3H/HeJ ♀. Female C3H/HeJ 0544/17

Hematoxylin and eosin stain

Notice the striking similarity in morphology when comparing with neoplasms from animals 0001-17 or 0005-17 (Figure 37, Figure 38, Figure 39) to 0547/17 (see Figure 41). Stromal infiltration with mononuclear cells is of similar nature and degree (**blue arrows**). Stroma also has similar levels of tumor-infiltrating lymphocytes (TIL). Although rather well differentiated, this neoplasm is clearly malignant, with good seeding potential, with also compression related dilated lymphatic vessels (red arrow). Cytological features of this tumor and the ones from all JA0023 derived tumors are very similar, near identical. The neoplasm is strikingly invasive in the adjacent stroma (not shown).

Morphological diagnosis: Adenocarcinoma (AdK), papillary-cystic, skin, mammary gland.

Low magnification of tumor aspect overview (thumbnail):

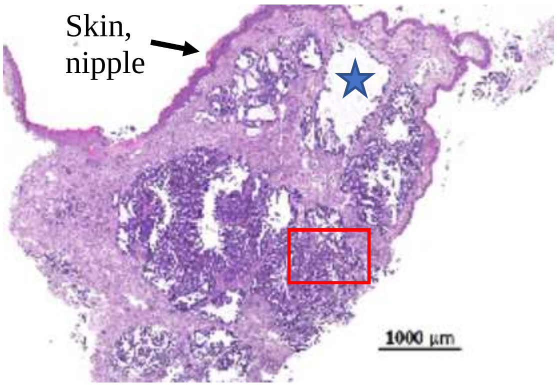

The red rectangle indicates the part of the tumor, which is depicted above at higher magnification.

Source photo file: 2017-10-25 15_08_41-TPL811_17_HE_10445_Tu_0544_17-Rotate-01 - ZEN 2.3 lite

At sub-gross examination, this ~ 3.6-mm thick tumor is developed in several multi-lobulated dilated or cystic (★) spaces, composed of tubule and papillary proliferations.

JA-0032 sMDI: Adenocarcinoma, Lung

JA-0032/ 0055-17 (Fig. 3). The original index sample was identified as a typical bronchiolo-alveolar papillary adenocarcinoma

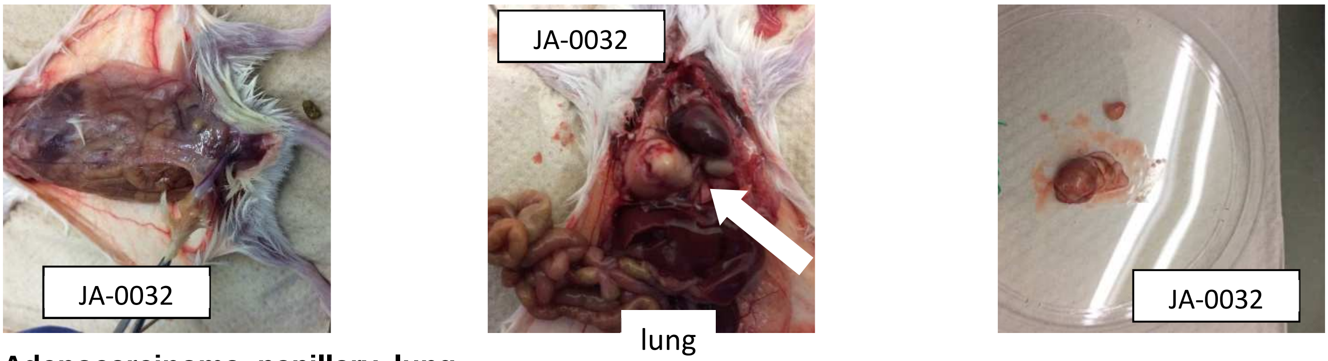

Adenocarcinoma, papillary, lung

Figure 43: Spontaneous neoplasms (study #10445): papillary adenocarcinoma, lung, male mouse 0032/14

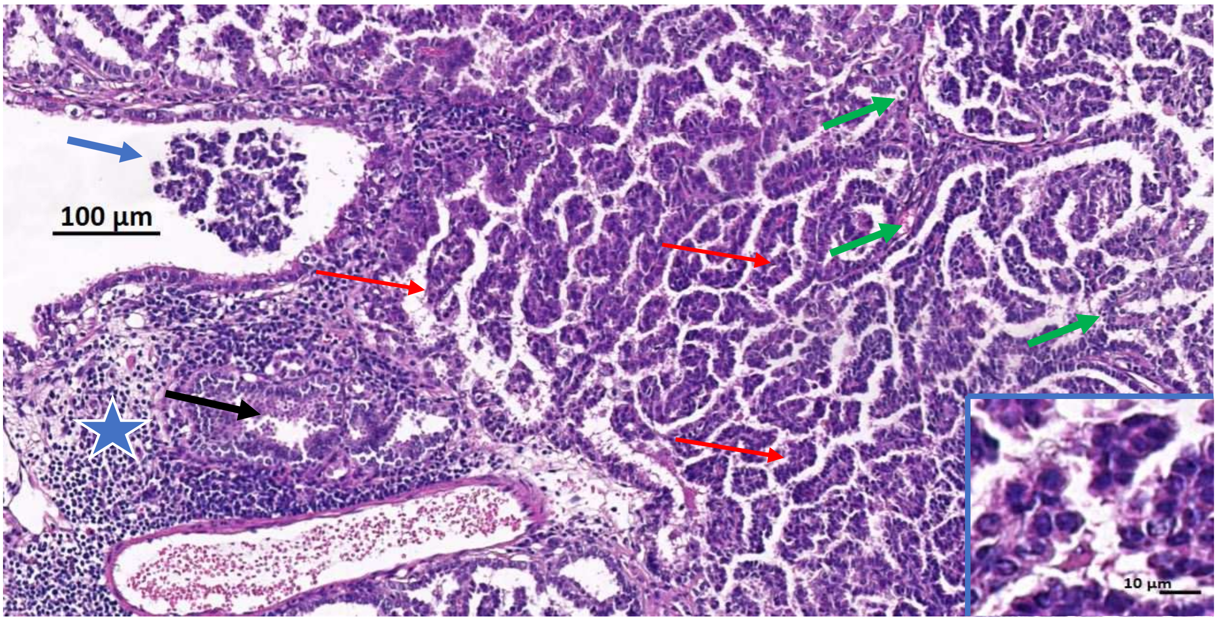

Index case: mouse ID number 0032-14, strain BALB/c, sex ♂.

Developed and invading the alveolar parenchyma are small cords, festoons and papillary structures supported by a delicate stroma, composed of mid-sized (~ 10 µm) cuboidal cells resembling type II pneumocytes (see **inset** and red arrows). This proliferative alveolar tissue invades the nearby main bronchus (**blue arrow**). The pre-existing alveolar walls, obscured by the proliferative tissue, as well as the delicate stroma induced by the neoplastic tissue is variably thickened by collagenous tissue and infiltrated by moderate numbers of small mononuclear cells (**green arrows**). The BALT has moderate lymphoid depletion (★) and focal invasion by neoplasm (**black arrow**).

Image ID: 2017-10-25 15_17_08-TPL811_17_HE_10445_Tu_32_15_3_4_17_Lu.czi - ZEN 2.3 lite

Morphological diagnosis: Adenocarcinoma (AdK), papillary-cystic

Low magnification of tumor aspect overview (thumbnail):

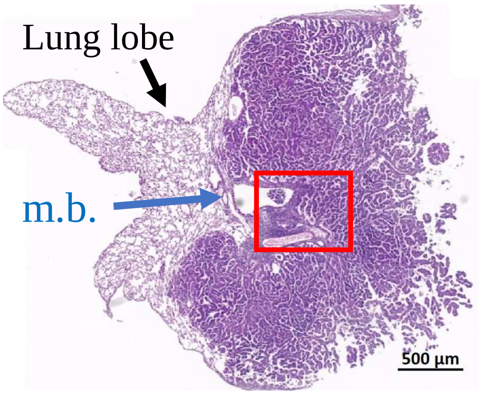

The red rectangle indicates the part of the neoplasm, which is depicted above

Blue arrow, m.b. = main bronchus

Source photo file: 2017-10-25 15_15_07-TPL811_17_HE_10445_Tu_32_14_3_4_17_Lu.czi - ZEN 2.3 lite

At sub-gross examination, this ~ 3.5-mm long neoplasm is developed in several multi-lobulated dilated or cystic ( ) spaces, composed of tubule and papillary proliferations.

Figure 44: Spontaneous neoplasms (study #10445): adenocarcinoma, solid, clear cells, subcutis, male mouse 0055/17

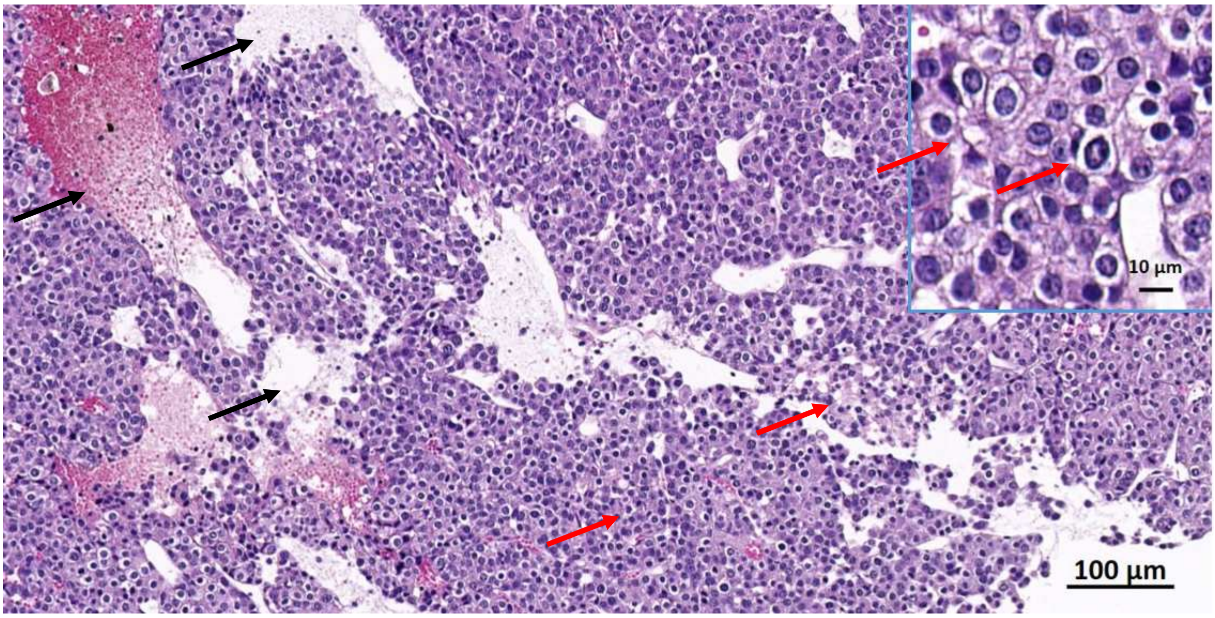

Source: 2017-10-25 15_19_23-TPL811_17_HE_10445_Tu_0055_17.czi - ZEN 2.3 lite

Index case: mouse ID number 0032-14, strain BALB/c, sex ♂

This case is not straightforward because the aspect of the source index case (see Figure 43) is morphologically very different from this derived one: in male 055/17, there is solid very fragile (due to nearly absent supportive stroma and secondary tearing and hemorrhage – see **black arrows**) solid proliferation of mid-sized ~ 10 µm polyhedral to cuboidal epithelial cells with characteristically clear ("rarefied") cytoplasm (see **red arrows**). Anisopoikilocytosis and nuclear variability are slight to moderate. This tumor is developed in the subcutis, locally when transplanted.

Morphological diagnosis: Adenocarcinoma (Adk), solid, clear cells, subcutis.

Figure 45: Low magnification of tumor aspect overview and high view detail (**thumbnails**)

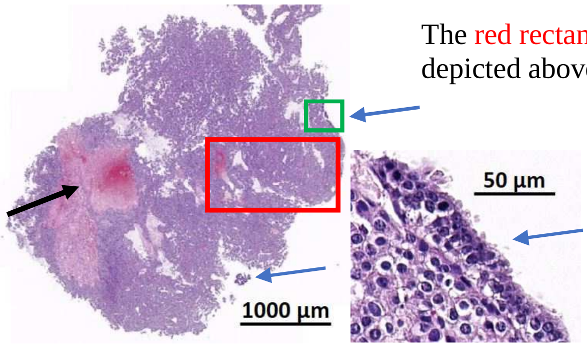

The red rectangle indicates the part of the tumor, which is depicted above, the green rectangle is depicted below:

Source photo files: 2018-01-22 16_07_12- and 2018-01-23 08_40_54-TPL811_17_HE_10445_Tu_0055_17.czi - ZEN 2.3 lite:
On the edge of the proliferation, small foci of epithelial cells similar to the case 0032-14 (blue arrows). The **black arrow** indicates a large area of necrosis and hemorrhages.

Comments on JA-0032 sMDI:

This tumor from 0055-17 is most probably derived from the index lung Adk. The clear cell aspect most probably is to be related to surfactant secreting type II pneumocytes - IHC would be useful to prove this hypothesis. Standard HE histology cannot prove it.

This situation is a frequent encounter, when the sites of grafting is different from the original organ of origin. An orthotopic (matching site in the lung) engraftment would be desirable if one is willing to recreate the original morphology (see the recent publication in Reference 2 for the pancreatic cancer situation.

In this particular case, if further used in research and development, orthotopic engraftments would be highly recommended, although heterotopic (ectopic) may be used, as long as the presence of key characteristics such as the surfactant and areas of alveolar morphology can be demonstrated

Literature cited:

Reference 1 James E. Talmadge, Marc E. Key and Ian R. Hart, 1981; "Characterization of murine ovarian reticulum cell sarcoma of histiocytic origin" May 1981 *Cancer Research* vol. 41(4):1271-80

Reference 2: Wanglong Qiu, and Gloria H Su. 2013; "Development of orthotopic pancreatic tumor mouse models" *Methods in molecular biology (Clifton, N.J.)* vol. 980 (): 215-23. https://www.ncbi.nlm.nih.gov/pmc/articles/PMC4049460/

1.4 Doc. S2-sMDI - Material, Methods & Results: Efficacy studies with anti-mPD-1 and anti-mCTLA-4 in seven syngeneic standard mouse tumor models

Implantation protocol for efficacy studies using immune checkpoint antibodies for cell line-based syngeneic tumor models: Cells from tissue culture were washed twice and suspended in PBS. On day 0 between $1x10^5$ and $1x10^6$ colon carcinoma cells MC38-CEA (CPQ 449*) in female C57BL/6N, colon carcinoma cells CT26.WT (CPQ 238) in female BALB/c, lung carcinoma cells LL/2 (CPQ 423) in female C57BL/6N, melanoma Clone M3 cells (CPQ 422) in female DBA/2N, mamma carcinoma 4T1 cells (CPQ 272) in female BALB/c, renal carcinoma RENCA cells (CPQ 307) in female BALB/c, and melanoma B16.F10 cells (CPQ 365**) in female C57BL/6N were injected subcutaneously according the respective tumor model.

*Cell lines are stock of ProQinase GmbH, In Vivo Pharmacology (CPQ-numbered), and derived from ATCC, LGC Standards GmbH, Germany. Carcinoembryonic Antigen (CEA) transfected MC38-CEA were kindly provided from Joanna Bereta, PhD , Jagiellonian University, Krakow, Poland. **B16.F10 cells were regularly selected for high metastatic capacity by i.v. application to induce *in vivo* metastases, followed by *in vitro* re-culturing of lung metastasis [1].

Study design for efficacy studies using immune checkpoint antibodies for cell line-based syngeneic standard tumor models: Mice were implanted with above mentioned cell lines according implantation protocol. Tumor sizes were calculated according to the formula $W^2xL/2$ (L= length and W= the perpendicular width of the tumor, L>W). When mean tumor size achieved a volume of about 100 - 200 mm^3, tumor-bearing animals were randomized (between days 4 – 20 – according respective tumor model) into 3 groups of 8 - 12 animals. For randomization, a robust automated random number generation within individual blocks was used (MS-Excel 2016).

Results are shown in Figure S1. Both colon carcinoma models, MC38-CEA and CT26.WT were significantly sensitive to anti-ICPI treatment: MC38-CEA growth was significantly reduced both, by anti-mCTLA-4 and anti-mPD-1 treatment. Growth of CT26.WT was significantly reduced by anti-mCTLA-4 treatment but showed only a non-significant reduction by anti-mPD-1 treatment. The other cell line-based syngeneic tumor standard models displayed no or no significant inhibition. A reduction of tumor growth that was not significant was observed in Clone M3 for treatment with anti-mCTLA4 antibodies and in B16.F10 for treatment with anti-mPD-1 treatment.

The strong inhibition of MC38-CEA contrasts with results of others [2], not seeing any effects by anti-ICPI antibodies in the MC38 model which probably results from transfection with CEA which acts as potential neoantigen in the cells (see also Doc. S2 and Fig. S2). As demonstrated by flow cytometry, the number and subpopulations of tumor-infiltrating immune cells does already differ in the two model subvariants (Fig. 4b) and [2]. Probability (P) was tested with parametric unpaired t test (GraphPad Prism 5.04) compared to PBS vehicle control. Differences were determined as not significant with ns > 0.050 and significant * with $p < 0.050$, ** with $p < 0.010$, or *** with $p < 0.001$.

1.5 *Fig. S2– sMD - Efficacy studies with anti-mPD-1 and anti-mCTLA-4 in seven syngeneic standard mouse tumor models*

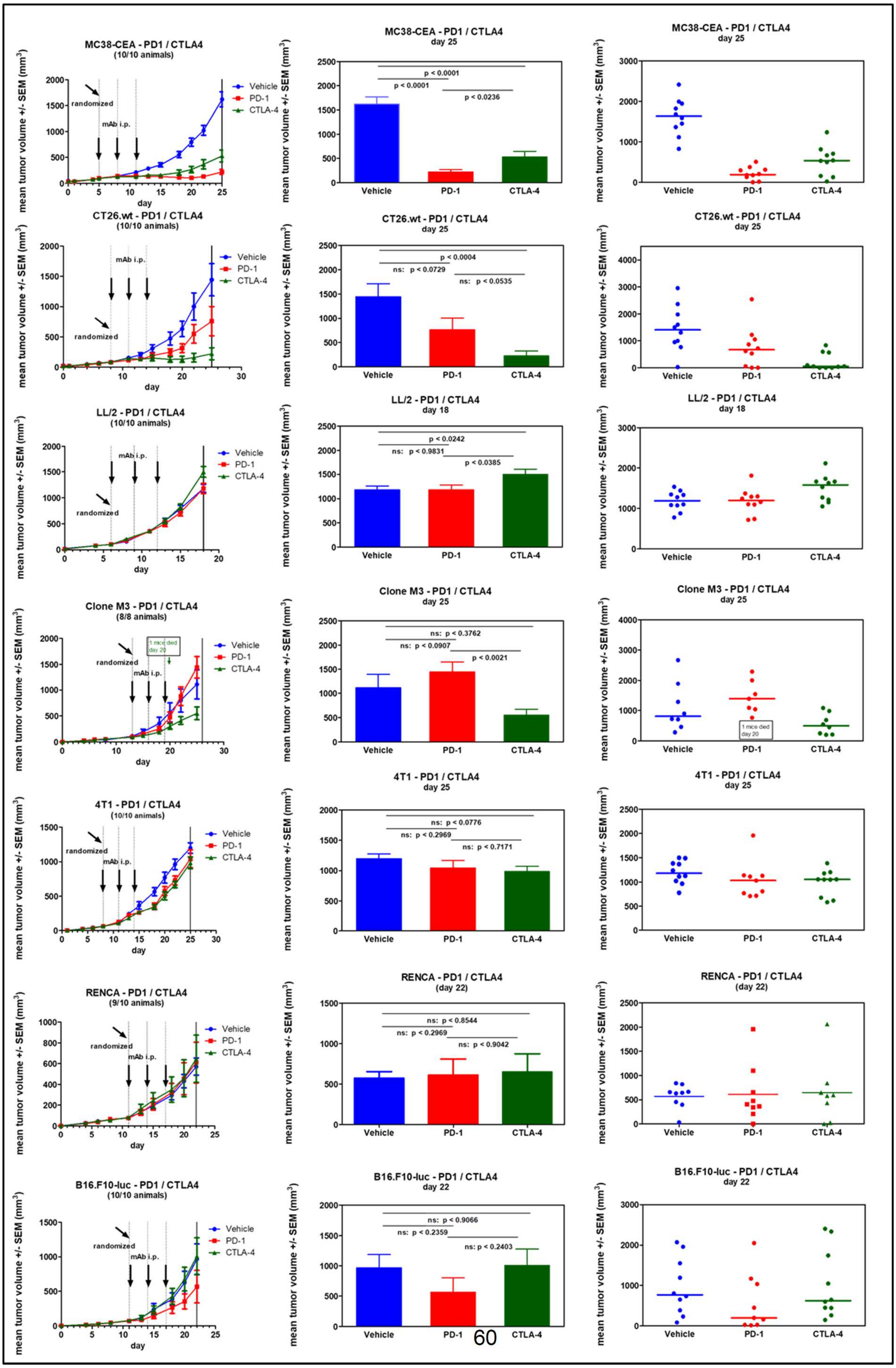

Fig. S1-sMDI - Efficacy of anti-ICPI antibodies in seven established syngeneic standard mouse tumor models

The tumors cells of colon carcinoma MC38-CEA, colon carcinoma CT26.WT, lung carcinoma LL/2, melanoma Clone M3, mamma carcinoma 4T1, renal carcinoma RENCA, and melanoma B16.F10 were each implanted subcutaneously into mice. Animals were randomized at tumor volumes of 100 - 200 mm^3 between days 5 - 13 according to the respective model into 8 mice per group. Mice were treated three times (dotted lines) i.p. with anti-ICPI antibodies and PBS as vehicle control: vehicle – blue (10 ml/kg), anti-mPD-1 – green (10 mg/kg), and anti-mCTLA-4 – red (10 mg/kg). Tumor volumes are shown as growth curve (curve chart), mean of groups (bar graphs), and individual values of single mouse per group (dot plots). Probability (P) was tested with parametric unpaired t test (GraphPad Prism 5.04) compared to PBS vehicle control. Differences were determined as not significant with ns > 0.050 and significant * with $p < 0.050$, ** with $p < 0.010$, or *** with $p < 0.001$.

1.6 *Doc. S3-sMDI - Material, Methods & Results: Establishment, efficacy and characterization of mPD-1-resistant MC38-CEA relapsed subline*

Establishment of MC38-CEA-PD1-res: A PD-1-resistent subline of MC38-CEA was established (Fig. S2A) from mouse 0007-15 which was part of the comparative study of seven standard syngeneic *in vivo* mouse tumor models (Fig. S1). The MC38-CEA tumor was completely regressed by 3-fold anti-mPD-1 treatment after day 25. Extended monitoring of this mouse showed weak, but again spontaneously regressing tumor growth between days 30 - 40. A striking relapse started with day 50. Re-treatment with mPD-1 starting on day 60 did no longer inhibit, in contrast to primary treatment (Fig. S1), tumor growth during further monitoring (Fig. S2A). On day 88 the mouse was sacrificed, tumor was removed sterile, cut into small pieces and cultured with RPMI 1640 medium in petri dishes. From outgrowing cells, a stable cell line was established by *in vitro* culture, frozen stored and designated as mPD-1-resistant MC38-CEA (MC38-CEA-PD1-res).

Implantation protocol and study design: Implantation and study design for MC38-CEA-PD1-res cell line was performed according the MC38-CEA protocols as described in Doc.S1-sMDI

Efficacy testing of MC38-CEA-PD1-res tumors: mPD1 resistant MC38-CEA cell line was implanted day 0. Since growth of the resistant cells was delayed compared to parental cells, randomization at tumor volumes of 40 - 80 mm^3 in 8 animals per group was performed only on day 13 (versus day 5 in parental cells – Fig. S2B). Starting on randomization day, mice with relapse-derived, MC38-CEA-PD-1 resistant tumors (Fig. S2C 1-3) were treated three times (dotted lines) i.p. with anti-ICPI antibodies and PBS as vehicle control: vehicle – blue (10 ml/kg), anti-mPD-1 – green (10 mg/kg), and anti-mCTLA-4 – red (10 mg/kg). As shown in Figs. S2C growth of the PD1-resistant tumors was insensitive to renewed mPD-1 treatment till day 25. Although anti-mCTLA-4 antibodies showed an about 50 % reduction of growth, also this difference was not significant. In contrast, parental colon carcinoma MC38-CEA was sensitive to mPD-1 as well as mCTLA-4 treatment (Fig. S2B**)**. Probability (P) was tested with parametric unpaired t test (GraphPad Prism 5.04) compared to PBS vehicle control. Differences were determined as not significant with ns > 0.050 and significant * with $p < 0.050$, ** with $p < 0.010$, or *** with $p < 0.001$.

Flow cytometric analysis: Expression of Carcinoembryonic Antigen (CEA) in transfected MC38-CEA and PD1-resistent MC38-CEA-PD1-res cells was compared using a panel of CEA-specific or CEA-family specific (13H10, HD11, CC11, AH119 or control (anti-PD-L1) antibodies [3,4] according flow cytometric analysis performed in the main paper. FACS buffer was used as vehicle control. The data clearly show that mPD-1-res MC38-CEA cells do actually express lower amount or no CEA anymore, whereas the parental cells express. Since ATCC MC38 was not anti-mPD-1 sensitive [2], we assume that probably CEA was the neoantigen on MC38 tumors, which was targeted by anti-mPD-1 treatment in MC38-CEA parental cells. Loss of this antigen made the cells insensitive to anti-mPD-1 immunotherapy. If the insignificant effect of mCTLA-4 also results from reduced CEA expression or might result from the fact that tumor volume in the vehicle group of mPD-1 resistant cells was only a half of tumor volume of parental cells at study end on day 25, remains to be verified.

Conclusion: Long-term observation of e.g. immuno- or chemotherapeutic treatment-dependent tumor regression as well as relapse later on, opens possibilities to establish actually therapy resistant sublines or -models, e.g. potential therapy-resistant MDI-models. This provides new possibilities to test clinically relevant concepts against tumors getting therapy resistant.

1. Jantscheff, P.; Schlesinger, M.; Fritzsche, J.; Taylor, L.A.; Graeser, R.; Kirfel, G.; Furst, D.O.; Massing, U.; Bendas, G. Lysophosphatidylcholine pretreatment reduces VLA-4 and P-selectin-mediated B16.F10 melanoma cell adhesion in vitro and inhibits metastasis-like lung invasion in vivo. Mol Cancer Ther 2011, 10, 186-197.
2. Mosely, S.I.; Prime, J.E.; Sainson, R.C.; Koopmann, J.O.; Wang, D.Y.; Greenawalt, D.M.; Ahdesmaki, M.J.; Leyland, R.; Mullins, S.; Pacelli, L., et al. Rational selection of syngeneic preclinical tumor models for immunotherapeutic drug discovery. Cancer Immunol Res 2017, 5, 29-41.
3. Jantscheff, P.; Bottger, V.; Price, M.; Micheel, B.; Kaiser, G.; Zotter, S.; Kotzsch, M.; Grossmann, H.; Karsten, U. Production and characterization of monoclonal antibodies against carcinoembryonic antigen (cea). Biomed Biochim Acta 1991, 50, 1261-1267.
4. Jantscheff, P.; Terracciano, L.; Lowy, A.; Glatz-Krieger, K.; Grunert, F.; Micheel, B.; Brummer, J.; Laffer, U.; Metzger, U.; Herrmann, R., et al. Expression of ceacam6 in resectable colorectal cancer: A factor of independent prognostic significance. J Clin Oncol 2003, 21, 3638-3646.

1.7 Fig. S3– sMDI - Establishment, efficacy and characterization of mPD-1-resistant MC38-CEA subline with anti-ICPI antibodies in syngeneic standard mouse tumor models

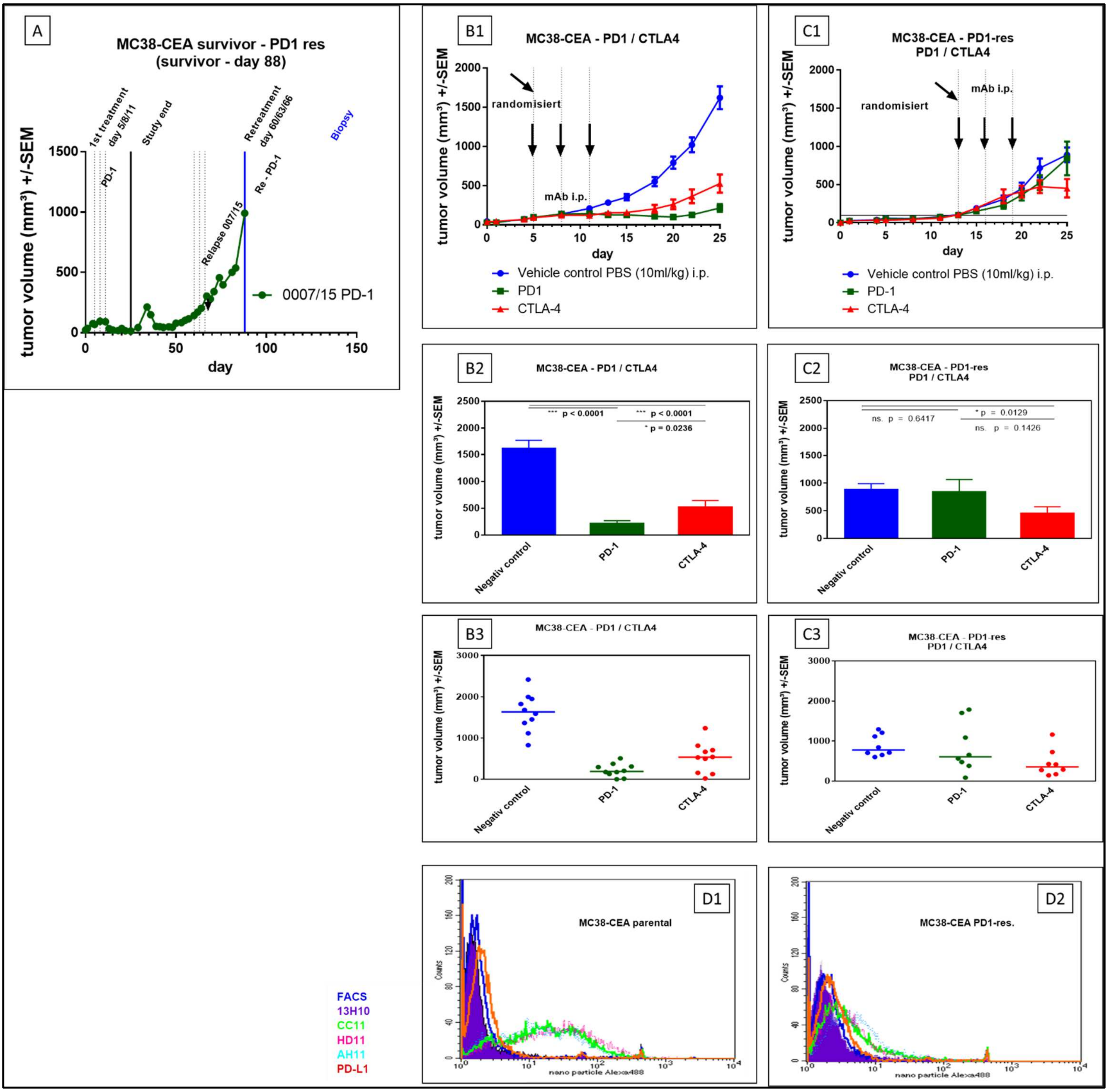

Fig. S2-sMDI - Establishment, efficacy and characterization of mPD-1-resistant MC38-CEA subline with anti ICPI antibodies in syngeneic standard mouse tumor models

Long-term observation of anti-mPD1 treatment caused regression of subcutaneous MC38-CEA colon carcinoma tumor growth in C57BL/6N female mouse 0007-15*, showed striking relapse with day 50 (A). Whereas MC38-CEA parental cells were sensitive to mPD1 as well as CTLA-4 treatment (B), newly established cell line MC38-CEA-PD1-res was insensitive (C) to three-fold (dotted lines) i.p. treatment with anti-ICPI antibodies, compared with PBS as vehicle control [vehicle (10 ml/kg), anti-mPD-1 (10

mg/kg), and anti-mCTLA-4 (10 mg/kg)]. Parental MC38-CEA (D1) and resistant subline (D2) also displayed striking different surface CEA expression as determined by flow cytometry analysis.

*Mouse 007/15 was part of the comparative study of seven standard syngeneic *in vivo* mouse tumor models which is shown in Fig. S1
